# Grignard Reagent Utilization Enables a Practical and Scalable Construction of 3-Substituted 5-Chloro-1,6-naphthyridin-4-one Derivatives

**DOI:** 10.3390/molecules25235667

**Published:** 2020-12-01

**Authors:** Ming-Shu Wang, Yi Gong, Zhi-Cheng Yu, Yan-Guang Tian, Lin-Sheng Zhuo, Wei Huang, Neng-Fang She

**Affiliations:** Key Laboratory of Pesticide & Chemical Biology of Ministry of Education, International Joint Research Center for Intelligent Biosensor Technology and Health, College of Chemistry, Central China Normal University, Wuhan 430079, China; wms@mails.ccnu.edu.cn (M.-S.W.); gy@mails.ccnu.edu.cn (Y.G.); yzc@mails.ccnu.edu.cn (Z.-C.Y.); tyguang1996@mails.ccnu.edu.cn (Y.-G.T.)

**Keywords:** 1,6-naphthyridine, Grignard reagent, kinase inhibitor, antitumor drug candidate

## Abstract

A robust, practical, and scalable approach for the construction of 3-substituted 5-chloro-1,6-naphthyridin-4-one derivatives **13** via the addition of Grignard reagents to 4-amino-2-chloronicotinonitrile (**15**) was developed. Starting with various Grignard reagents, a wide range of 3-substituted 5-chloro-1,6-naphthyridin-4-one derivatives **13** were conveniently synthesized in moderate-to-good yields through addition–acidolysis–cyclocondensation. In addition, the robustness and applicability of this synthetic route was proven on a 100 g scale, which would enable convenient sample preparation in the preclinical development of 1,6-naphthyridin-4-one-based MET-targeting antitumor drug candidates.

## 1. Introduction

Naphthyridine scaffolds represent attractive building blocks widely used in pharmaceuticals [1,2], agrochemicals [3], and fluorescent probes [4,5,6]. Recently, it has been reported that numerous naphthyridine-containing molecules have promising medicinal properties for the prevention and treatment of angiogenic disorders [7], viral infections [8,9], bacterial infections [10,11,12], parasitic infections [13], heart failure [14], Alzheimer’s disease (AD) [15], and cancers [16,17,18,19,20,21,22,23,24]. Therefore, the construction of naphthyridines has received increasing attention recently [25].

**Figure 1:**
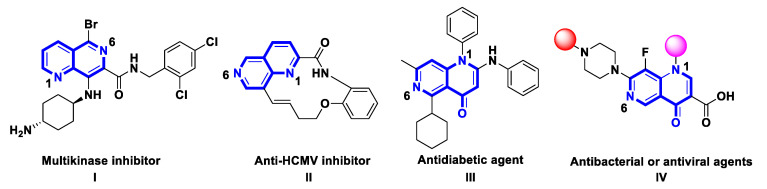
Representative 1,6-naphthyride- or 1,6-naphthyridin-4-one-based pharmacologically active compounds.

There are six naphthyridine isomers (1,5-, 1,6-, 1,7-, 1,8- 2,6-, and 2,7-pyridopyridines) [2]. Nowadays, the synthesis of 1,6-naphthyridine, especially for 1,6-naphthyridin-4-one derivatives, has attracted continuous interest in the pharmaceutical industry for its wide-ranging pharmacological activity [26,27], and it has been used as a multi-kinase inhibitor (**I**) [28], an anti-HCMV inhibitor (**II**) [29], an antidiabetic agent (**III**) [30], and antibacterial [31] and antiviral agents (**IV**) [1] (Figure 1).

**Figure 2:**
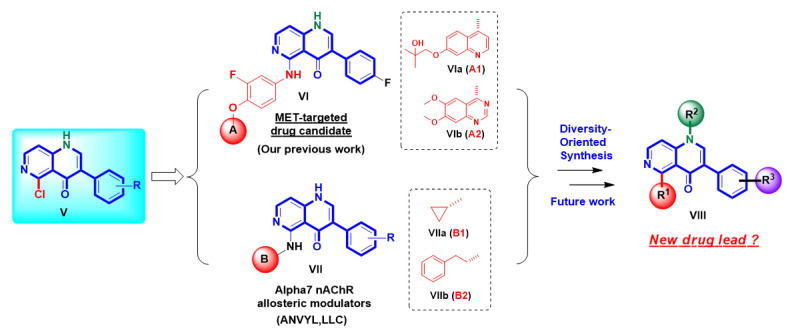
Discovery of bioactive 5-amino-3-phenyl-1,6-naphthyridin-4-one derivatives **VI**, **VII** and proposed future work.

In our previous report, 3-phenyl-1,6-naphthyridin-4-one was developed as a potent privileged skeleton for the discovery of a MET kinase inhibitor [20,21,22,23] from the key building block of 5-chloro-3-phenyl-1,6-naphthyridin-4-one (**V**). Extensive structure–activity relationship (SAR) studies and biological evaluations resulted in the discovery of two MET-targeting antitumor drug candidates, namely, **VIa [20]** and **VIb [23]**, which are near the clinical stage (Figure 2). In addition to being used as MET kinase inhibitors, 3-phenyl-1,6-naphthyridin-4-one derivatives have also been reported as α7 nAChR allosteric modulators **VII** by ANVYL, LLC and its copartner (Figure 2) [32,33]. Thus, we propose that diversity-oriented synthesis (**DOS**), starting from 5-chloro-3-phenyl-1,6-naphthyridin-4-one (**V**), would resulted in more new drug lead compounds **VIII** based on the highly reactive 5-halo group and the functionalizable *NH* group.

**Scheme 1:**
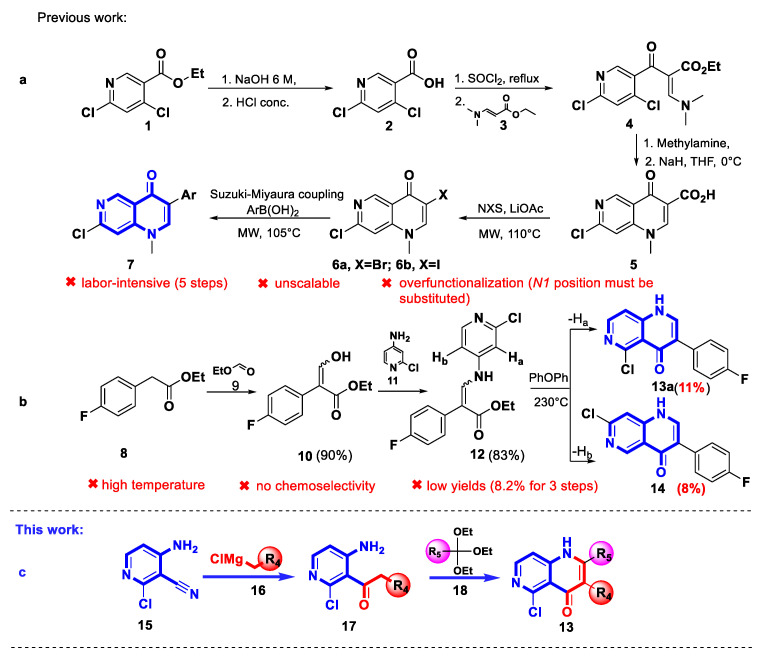
Common construction strategies of the 3-phenyl-1,6-naphthyridin-4-one scaffold: (**a**) Suzuki–Miyaura coupling-based construction of 7-functionalized 1,6-naphthyridin-4-one **7**; (**b**) Conrad–Limpach cyclizations-based construction of 5-chloro-3- substituted-1,6-naphthyridin-4-one derivatives **13a**; (**c**) Grignard reagent-based strategy for the construction of diversified 5-chloro-3- substituted-1,6-naphthyridin-4-one derivatives **13**.

However, the construction of the key 3-phenyl-1,6-naphthyridin-4-one skeleton has proven to be difficult. Currently, the reported procedures for its synthesis can be mainly divided into two types (Scheme 1a,b). The first method involves the preparation of an ethyl-2-(4,6-dichloro-nicotinoyl)-3-(dimethylamino)acrylate intermediate (**4**), followed by strong base-catalyzed cyclization to generate the desired naphthyridine scaffold **5**. Then, the halodecarboxylation of intermediate **5** followed by Suzuki–Miyaura coupling with an arylboronic acid results in a 7-functionalized 1,6-naphthyridin-4-one **7** (Scheme 1a) [34]. Obviously, the synthetic route described above has inherent issues, including: (i) it is a labor-intensive process (five-step sequence); (ii) non-applicability on a large scale (due to the physical limitation of microwave heating, the penetration depth of only a few centimeters, and the limited dimensions of standing wave cavities) [35]; (iii) overfunctionalization of the *N*-position restricts its application in the drug discovery process for which *N*-substituents need to be finely regulated (the synthesis of the 1,6-naphthyridin-4-one scaffold, keeping a free *NH*, using this synthetic route has not been reported and the introduction of *N*-substituents in the initial step of the synthetic route makes the substituent regulation process very cumbersome) [36]. The second method was reported by ANVYL,LLC in their α7 nAChR allosteric modulators study [33], as well as our team in our MET kinase inhibitor study (Scheme 1b) [22]; knowledge of the mechanism of Conrad–Limpach cyclizations led to the introduction of a thermal cyclization to generate the desired naphthyridine scaffold. However, thermal cyclization is typically carried out in diphenyl ether at 230 °C, and the lost control of chemoselectivity results in the generation of **13a** and **14** in almost the same proportions. The high reaction temperature and the lack of chemoselectivity have resulted in the complex synthesis of product compositions and low yields (8.2% for three steps), consequently leading to a labor-intensive enrichment and purification process.

Altogether, the construction of 3-phenyl-1,6-naphthyridin-4-one derivatives is still a challenging subject. With our ongoing research interests in the discovery and development of 1,6-naphthyridin-4-one derivatives as drug candidates, we propose a facile Grignard reagent-based strategy through addition–acidolysis–cyclocondensation to provide diversified 5-chloro-3-substituted-1,6-naphthyridin-4-one derivatives **13** (Scheme 1c).

## 2. Results and Discussion

**Scheme 2:**
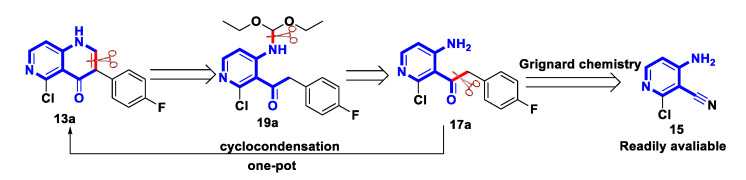
Retrosynthetic analysis of 5-chloro-3-(4-fluorophenyl)-1,6-naphthyridin-4-one.

First, 5-chloro-3-(4-fluorophenyl)-1,6-naphthyridin-4-one (**13a**), which is the key common intermediate of our reported MET-targeting drug candidates, was chosen as a target molecule for the preliminary exploration of new synthetic routes. By retrosynthetic analysis, we reasoned that the cyclocondensation of 1-(4-amino-2-chloropyridin-3-yl)-2-(4-fluorophenyl)ethan-1-one (**17a**) and triethyl orthoformate would provide the desired molecule **13a** (Scheme 2). Moreover, our way to intermediate (**17a**) is based on the inclusion of a nitrile substituent at the 3-position of 4-amino-2-chloronicotinonitrile to enable the generation of an α-keto group via Grignard chemistry.

**Scheme 3:**
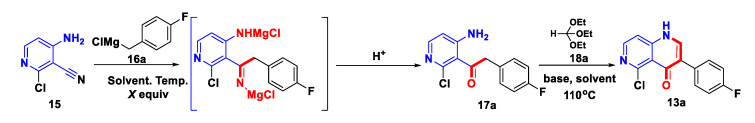
An overall synthetic route of 5-chloro-3-(4-fluorophenyl)-1,6-naphthyridin-4-one.

**Scheme 4:**
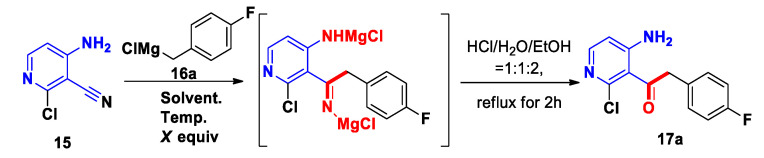
Our planned route to key intermediate **17a**.

The overall synthetic route of 5-chloro-3-(4-fluorophenyl)-1,6-naphthyridin-4-one (**13a**) is shown in Scheme 3. The formation of 1-(4-amino-2-chloropyridin-3-yl)-2- (4-fluorophenyl)ethan-1-one (**17a**) was investigated first using 4-amino-2-chloronicotinonitrile (**15**) and 4-fluorobenzylmagnesium chloride (**16a**) (Scheme 4 and Table 1).

We initially subjected the two reactants to reaction at 25 °C for 16 h in two different solvents commonly used in Grignard chemistry, and it is obvious that diethyl ether exhibited a better effect on the reaction to provide product **17a** than THF (Table 1, entries 1 and 2). It is also worth noting that the incomplete consumption of 4-amino-2-chloronicotinonitrile (**15**) was observed. Taking into consideration that lower temperatures can result in incomplete consumption and require longer reaction times, the reaction was warmed to 30 °C. After 12 h of reaction, the consumption of 4-amino-2-chloronicotinonitrile (**15**) was no longer detected by HPLC dynamic detection, and the desired product **17a** was obtained at a 35% yield (Table 1, entry 3). Due to the low boiling point of ether, the temperature could not be increased further. Thus, different inert solvents with higher boiling points were evaluated as latent solvents in view of their versatility demonstrated in the Grignard reaction. Regrettably, lower yields were obtained if methyl *tert*-butyl ether (MTBE) and isopropyl ether (IPE) were utilized (Table 1, entries 4 and 5), and toluene did not favor the transformation either (Table 1, entry 6). Finally, we optimized the reaction by changing the concentration of the Grignard reagent. As highlighted in Table 1 (entries 7–9), increasing the amount of Grignard reagent to 4.0 eqs afforded total consumption of 4-amino-2-chloronicotinonitrile (**15**) and resulted in a 74% yield of the desired product (Table 1, entries 7 and 8). However, a further increase did not result in a further increase in yield (Table 1, entry 9), suggesting that 4.0 equiv. is the best amount. In summary, 1.0 equiv. of 4-amino-2-chloronicotinonitrile (**15**) and 4.0 equiv. of 4-fluorobenzylmagnesium chloride (**16a**) in ether at 30 °C for 12 h were determined to be the optimum reaction conditions. 

**Scheme 5:**
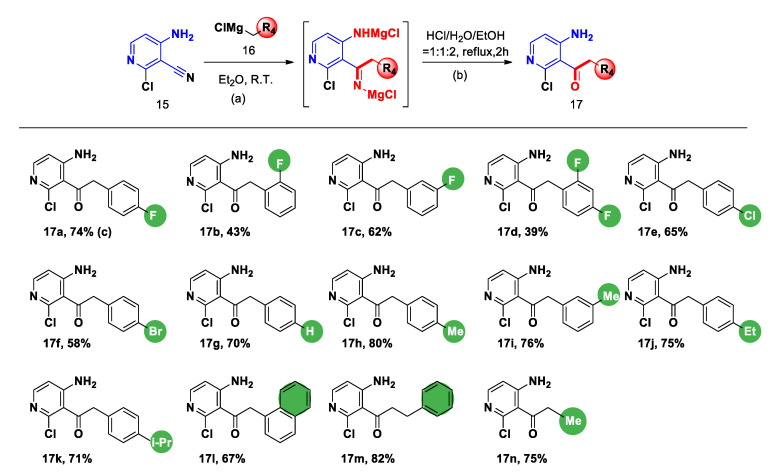
Grignard reagent scope for the construction of 1, 6-naphthyridin-4-one scaffold. (**a**) 1.5 mmol of 4-amino-2-chloronicotinonitrile (**15**) and 6.0 mmol of Grignard reagent (**16**) in 3.0 mL of ether at 30 °C for 12 h under an N_2_ atmosphere; (**b**) 6 mL of the HCl/H_2_O/EtOH (1:1:2) solvent was added for acidolysis; (**c**) Isolated yield.

With the optimized conditions established, the substrate scope with respect to the Grignard reagent was then evaluated (Scheme 5). It was found that the various Grignard reagents exposed to the optimized conditions smoothly provided the corresponding products **17a**–**n** in moderate to good yields (43–82%). Typically, electron-withdrawing halogens on the benzyl Grignard reagent were well tolerated to provide the corresponding products in moderate-to-good yields (**17a**, **17c**, **17e**, and **17f**; 58–74%); only the fluorine atom at the C-2 position of the benzyl group had a negative impact on the yield of the final products, which resulted in a relative lower yield of **17b** and **17d** with yields of 43% and 39%, respectively. Notably, an electron-donating group (EDG) might be slightly better than an electron-withdrawing group (EWG), as exemplified by some typical EDGs, the alkyl-substituted benzyl Grignard reagents **17h**–**j** (75–80%), including the bulky isopropyl group (**17k**; 71%). Moreover, a naphthyl Grignard reagent and chain-growing alkyl Grignard reagents, such as phenethyl and ethyl Grignard reagents, could also provide good yields (**17ln**; 67–82%).

**Scheme 6:**
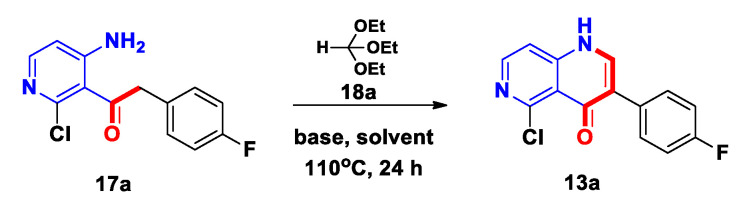
Our planned route to target molecule **13a**.

With these positive results in hand, the optimal reaction conditions of the cyclocondensation of 1-(4-amino-2-chloropyridin-3-yl)-2-(4-fluorophenyl)ethan-1-one (**17a**) were investigated next (Scheme 6 and Table 2). Initially, we investigated cyclocondensation by employing K_2_CO_3_ as a base and DMF as a solvent at a temperature of 110 °C. However, only a trace amount of the desired product was formed (Table 2, entry 1). Moreover, the combination of NaH and THF did not work (Table 2, entry 2). This was improved when we switched the inorganic base to an organic base. A 15% yield of the desired product was observed when Et_3_N was used (Table 2, entry 3), and a consistent improvement in the yield was observed by replacing Et_3_N with DIPEA or DMAP (Table 2, entries 4 and 5), suggesting that DMAP is the best base. Then, by utilizing DMAP as the base, the solvent effects were also investigated. When triethoxymethane was used as both the reactant and solvent, the yield of the desired product was slightly increased (Table 2, entry 6). Finally, we turned our attention to base loading. By gradually reducing the amount of base, we found that only a catalytic amount of the base was needed (Table 2, entries 6–9).

**Scheme 7:**
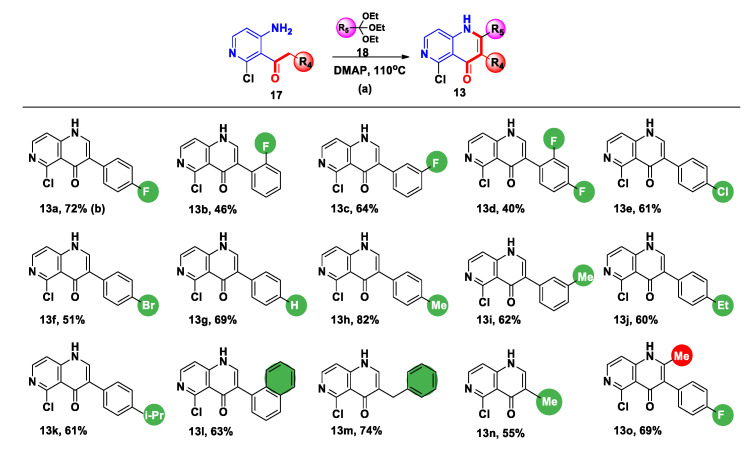
Synthesis of 1,6-naphthyridin-4-one derivatives. (**a**) 0.5 mmol of 1-(4-amino-2-chloropyridin-3-yl)-2-substituted-ethan-1-one (**17**) and 0.05 mmol of DMAP in 1.0 mL triethyl orthoformate (**13a**–**n**) or triethyl orthoacetate (**13o**) at 110 °C for 24 h; (**b**) Isolated yield.

Next, all of the intermediates synthesized above were subjected to the optimized reaction conditions. To our delight, the reaction demonstrated good compatibility, and a series of 3-substituted 1,6-naphthyridin-4-one derivatives were obtained smoothly with moderate-to-good yields (Scheme 7, **13a**–**o**; 40–82%). Generally, both 3-aryl-1,6-naphthyridin-4-one derivatives (**13al**; 40–82%) and 3-alkyl-1,6-naphthyridin-4-one derivatives (**13m** and **13n**; 74% and 55%) could be readily constructed, and the substituents on the benzene ring had less influence on the yields, except for an *ortho*-fluorine (**13b** and **13d**; 46% and 40%). Moreover, through changing the reactant triethyl orthoformate into triethyl orthoacetate, a methyl could be smoothly introduced to the C-2 position of the scaffold (**13o**, 69%), which is of great benefit to the study of SARs in drug discovery. Taken together, this approach is a general method for building various 3-substituted 5-chloro-1,6-naphthyridin-4-one derivatives.

**Scheme 8:**
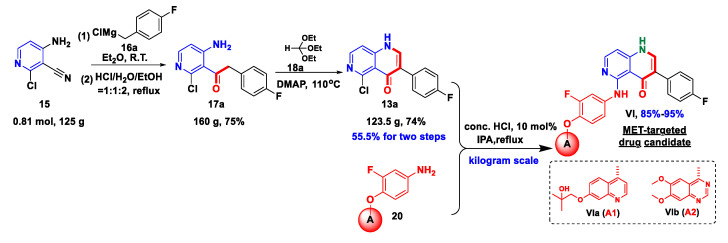
The 100 g scale synthesis of 5-chloro-3-(4-fluorophenyl)-1,6-naphthyridin-4-one and its application in preclinical drug candidate enrichment.

More importantly, the synthesis of compound **13a** was performed on a 100 g scale to demonstrate the robustness and applicability of this synthetic route (Scheme 8). By starting from 153.0 g (1.0 mol) of 4-amino-2-chloronicotinonitrile (**15**), we obtained the desired product at a yield of 53.3% (total yield for two steps). After completing the enrichment of compound **13a**, a kilogram- scale protocol for the creation of our 1,6-naphthyridin-4-one-based MET-targeting antitumor drug candidate **VI** was applied through acid-catalyzed nucleophilic substitution of 5-Cl atoms with high yields (90%) and simple operation (insensitive to moisture and oxygen; filtration to obtain desired pure product). This would enable convenient sample preparation in the preclinical development of this drug candidate.

## 3. Conclusions

In conclusion, we have developed a robust, practical, and scalable approach for the construction of 3-substituted 5-chloro-1,6-naphthyridin-4-one derivatives **13** via the addition of Grignard reagents to 4-amino-2-chloronicotinonitrile (**15**). Starting with various Grignard reagents through addition–acidolysis–cyclocondensation, a wide range of 5-chloro-3-substituted-1,6-naphthyridin-4-one derivatives **13** were synthesized in moderate-to-good yields. In addition, the robustness and applicability of this synthetic route was proven on a 100 g scale, which would enable convenient sample preparation in the preclinical development of 1,6-naphthyridin-4-one-based drug candidates. Further extensions of these naphthyridine derivatives in drug discovery are currently underway in our laboratories. 

## 4. Materials and Methods

### 4.1. General Information

Unless otherwise noted, all chemical reagents were commercially available and treated with standard methods. Silica gel column chromatography (CC). silica gel (200–400 Mesh; Qingdao Makall Group Co., Ltd; Qingdao; China). Solvents were dried in a routine way and redistilled. All reactions involving air- or moisture-sensitive reagents were performed under a nitrogen or argon atmosphere. Melting points of compounds were measured on a Melt-Temp II apparatus (BUCHI Labortechnik AG, Uster, Switzerland) and are uncorrected. ^1^H-NMR spectra (400 MHz) and ^13^C-NMR (100 MHz) spectra were recorded on an Ultrashield Plus AV 400 spectrometer (Bruker BioSpin AG, Fällanden, Switzerland) as dimethyl sulfoxide-*d_6_* (DMSO-*d_6_*) solutions using tetramethylsilane (TMS) as an internal standard (δ = 0) unless noted otherwise. ^1^H-NMR spectra (600 MHz) and ^13^C-NMR (150 MHz) spectra were recorded on a Varian Mercury-Plus 600 spectrometer (Varian Inc., Palo Alto, CA, USA) as dimethyl sulfoxide-*d_6_* (DMSO-*d_6_*) solutions using tetramethylsilane (TMS) as an internal standard (δ = 0) unless noted otherwise, and all the ^1^H and ^13^C NMR Spectra are summarized in the Supplementary Materials. MS spectra were obtained on a 6120 quadrupole LC/MS (ESI, Agilent Technologies, Santa Clara, CA, USA). All reactions were monitored using thin-layer chromatography (TLC, Yantai Jiangyou Silica gel Development Co., Ltd.; Yantai; China) on silica gel plates. Yields were of purified compounds and were not optimized.

### 4.2. General Procedure I for the Synthesis of 2-Substituted 1-(4-amino-2-chloropyridin-3-yl)-ethan-1-ones 17a–n

The appropriate Grignard reagent **16** (6.0 mmol, 4.0 equivs.) in ether (3.3 mL) was added at room temperature to 4-amino-2-chloronicotinonitrile (**15**, 230.0 mg, 1.5 mmol) in ether (3.0 mL). The reaction mixture was warmed to 30 °C and stirred for 12 h under ether atmosphere. Then the reaction mixture was cooled to 0 °C and 6.0 mL of mixed solvent (HCl/H_2_O/EtOH = 1:1:2) was added dropwise, the reaction mixture was refluxed for a further 2 h. After the completion of the reaction, the mixture was neutralized with sodium carbonate solution and extracted with EtOAc (3 × 15 mL). The organic layer was dried with anhydrous sodium sulfate, filtered, and concentrated. The residue was purified by chromatography (hexane/EA = 5:1) to yield the corresponding compound **17** in good yield.

*1-(4-Amino-2-chloropyridin-3-yl)-2-(4-fluorophenyl)ethan-1-one* (**17a**) was prepared from **15** and (4-fluorobenzyl)magnesium chloride (**16a**) by general procedure I as a white solid (294 mg, 74%), M.p. 130–133 °C, ^1^H-NMR (400 MHz, DMSO-*d*_6_) *δ* 7.81 (d, *J* = 6.0 Hz, 1H), 7.3 3–7.30 (m, 2H), 7.17–7.12 (m, 2H), 6.61 (d, *J* = 6.0 Hz, 1H), 6.59 (s, 2H), 4.18 (s, 2H). ^13^C-NMR (100 MHz, DMSO-*d*_6_) *δ* 201.5, 161.5 (d, *J_C_*_–*F*_ = 241.0 Hz), 153.7, 149.2, 146.7, 132.3(d, *J_C_*_–*F*_ = 8.0 Hz), 130.5(d, *J_C_*_–*F*_ = 3.0 Hz), 118.8, 115.3(d, *J_C_*_–*F*_ = 24.0 Hz), 110.5, 49.1. MS (ESI): 265.05 [M + H]^+^.

*1-(4-Amino-2-chloropyridin-3-yl)-2-(2-fluorophenyl)ethan-1-one* (**17b**) was prepared from **15** and (2-fluorobenzyl)magnesium chloride (**16b**) by general procedure I as a white solid (171 mg, 43%), M.p. 127–131 °C, ^1^H-NMR (400 MHz, DMSO-*d*_6_) *δ* 7.82 (d, *J* = 6.0 Hz, 1H), 7.47–7.25 (m, 2H), 7.24–7.07 (m, 2H), 6.62 (s, 2H), 6.60 (d, *J* = 6.0 Hz, 1H), 4.26 (s, 2H). ^13^C-NMR (100 MHz, DMSO-*d*_6_) *δ* 200.2, 162.2 (d, *J_C_*_–*F*_ = 243.0 Hz), 154.1, 149.3, 147.1, 133.1 (d, *J_C_*_–*F*_ = 5.0 Hz), 129.6 (d, *J_C_*_–*F*_ = 8.0 Hz), 124.6 (d, *J_C_*_–*F*_ = 3.0 Hz), 121.7 (d, *J_C_*_–*F*_ = 16.0 Hz), 118.3, 115.5 (d, *J_C_*_–*F*_ = 21.0 Hz), 110.7, 43.8. MS (ESI): 265.05 [M + H]^+^.

*1-(4-Amino-2-chloropyridin-3-yl)-2-(3-fluorophenyl)ethan-1-one* (**17c**) was prepared from **15** and (3-fluorobenzyl)magnesium chloride (**16c**) by general procedure I as a white solid (246.3 mg, 62%), M.p. 128–132 °C, ^1^H-NMR (400 MHz, DMSO-*d*_6_) *δ* 7.81 (d, *J* = 6.0 Hz, 1H), 7.43–7.29 (m, 1H), 7.18–7.02 (m, 3H), 6.62 (s, 2H), 6.60 (d, *J* = 6.0 Hz, 1H), 4.23 (s, 2H). ^13^C-NMR (100 MHz, DMSO-*d*_6_) *δ* 201.0, 162.4 (d, *J_C_*_–*F*_ = 241.0 Hz), 153.8, 149.2, 146.7, 137.1 (d, *J_C_*_–*F*_ = 8.0 Hz), 130.4 (d, *J_C_*_–*F*_ = 8.0 Hz), 126.6 (d, *J_C_*_–*F*_ = 2.0 Hz), 118.8, 117.2 (d, *J_C_*_–*F*_ = 21.0 Hz), 114.1 (d, *J_C_*_–*F*_ = 21.0 Hz), 110.6, 49.5. MS (ESI): 265.05 [M + H]^+^.

*1-(4-Amino-2-chloropyridin-3-yl)-2-(2,4-difluorophenyl)ethan-1-one* (**17d**) was prepared from **15** and (2,4-*d*ifluorobenzyl)magnesium chloride (**16d**) by general procedure I as a white solid (165.3 mg, 39%), M.p. 180–183 °C, ^1^H-NMR (400 MHz, DMSO-*d*_6_) *δ* 7.83 (d, *J* = 6.0 Hz, 1H), 7.50–7.38 (m, 1H), 7.26–7.15 (m, 1H), 7.11–7.01 (m, 1H), 6.62 (s, 2H), 6.60 (d, *J* = 6.0 Hz, 1H), 4.25 (s, 2H). ^13^C-NMR (100 MHz, DMSO-*d*_6_) *δ* 200.1, 161.9 (dd, *J_C_*_–*F*_ = 241.0 Hz, 12.0 Hz), 161.2 (dd, *J_C_*_–*F*_ = 241.0 Hz, 12.0 Hz), 154.1, 149.3, 147.1, 134.0 (dd, *J_C_*_–*F*_ = 9.0 Hz, 6.0 Hz), 118.2, 118.1 (dd, *J_C_*_–*F*_ = 15.0 Hz, 3.0 Hz), 111.6 (dd, *J_C_*_–*F*_ = 21.0 Hz, 5.0Hz), 110.7, 103.9 (t, *J_C_*_–*F*_ = 26.0 Hz), 43.2. MS (ESI): 283.16 [M + H]^+^.

*1-(4-Amino-2-chloropyridin-3-yl)-2-(4-chlorophenyl)ethan-1-one* (**17e**) was prepared from **15** and (4-chlorobenzyl)magnesium chloride (**16e**) by general procedure I as a white solid (273 mg, 65%), M.p.147–150 °C, ^1^H-NMR (400 MHz, DMSO-*d*_6_) *δ* 7.82 (d, *J* = 6.0 Hz, 1H), 7.38 (d, *J* = 8.4 Hz, 2H), 7.31 (d, *J* = 8.4 Hz, 2H), 6.62 (s, 2H), 6.60 (d, *J* = 6.0 Hz, 1H), 4.19 (s, 2H). ^13^C-NMR (100 MHz, DMSO-*d*_6_) *δ* 201.2, 153.7, 149.2, 146.7, 133.3, 132.2, 132.0, 128.5, 118.7, 110.5, 49.2. MS (ESI): 282.01 [M + H]^+^.

*1-(4-Amino-2-chloropyridin-3-yl)-2-(4-bromophenyl)ethan-1-one* (**17f**) was prepared from **15** and (4-bromobenzyl)magnesium chloride (**16f**) by general procedure I as a white solid (283.2 mg, 58%), M.p. 132–133 °C, ^1^H-NMR (400 MHz, DMSO-*d*_6_) *δ* 7.82 (d, *J* = 6.0 Hz, 1H), 7.52 (d, *J* = 8.4 Hz, 2H), 7.25 (d, *J* = 8.4 Hz, 2H), 6.62 (s, 2H), 6.60 (d, *J* = 6.0 Hz, 1H), 4.18 (s, 2H). ^13^C-NMR (100 MHz, DMSO-*d*_6_) *δ* 201.1, 153.7, 149.2, 146.7, 133.8, 132.6, 131.4, 120.5, 118.7, 110.5, 49.3. MS (ESI): 326.96 [M + H]^+^.

*1-(4-Amino-2-chloropyridin-3-yl)-2-phenylethan-1-one* (**17g**) was prepared from **15** and benzyl-magnesium chloride (**16g**) by general procedure I as a white solid (258.9 mg, 70%), M.p.85–87 °C, ^1^H-NMR (400 MHz, DMSO-*d*_6_) *δ* 7.81 (d, *J* = 6.0 Hz, 1H), 7.33–7.26 (m, 5H), 6.62–6.59 (m, 3H), 4.20 (s, 2H). ^13^C-NMR (100 MHz, DMSO-*d*_6_) *δ* 201.6, 153.8, 149.1, 146.7, 134.3, 130.3, 128.6, 127.2, 118.8, 110.5, 50.0. MS (ESI): 247.06 [M + H]^+^.

*1-(4-Amino-2-chloropyridin-3-yl)-2-(p-tolyl)ethan-1-one* (**17h**) was prepared from **15** and (4-methyl-benzyl)magnesium chloride (**16h**) by general procedure I as a white solid (312 mg, 80%), M.p. 100–103 °C, ^1^H-NMR (400 MHz, DMSO-*d*_6_) *δ* 7.80 (d, *J* = 6.0 Hz, 1H), 7.15 (d, *J* = 8.0 Hz, 2H), 7.11 (d, *J* = 8.0 Hz, 2H), 6.60 (d, *J* = 6.0 Hz, 3H), 4.13 (s, 2H), 2.28 (s, 3H). ^13^C-NMR (100 MHz, DMSO-*d*_6_) *δ* 201.7, 153.8, 148.9, 146.6, 136.2, 131.2, 129.2, 118.8, 110.5, 49.7, 21.1. MS (ESI): 261.32 [M + H]^+^.

*1-(4-Amino-2-chloropyridin-3-yl)-2-(m-tolyl)ethan-1-one* (**17i**) was prepared from **15** and (3-methyl-benzyl)magnesium chloride (**16i**) by general procedure I as a white solid (297 mg, 76%), M.p. 91–95 °C, ^1^H-NMR (600 MHz, DMSO-*d*_6_) *δ* 7.80 (d, *J* = 6.0 Hz, 1H), 7.19 (t, *J* = 7.2 Hz, 1H), 7.12–7.01 (m, 3H), 6.65–6.52 (m, 3H), 4.14 (s, 2H), 2.28 (s, 3H). ^13^C-NMR (150 MHz, DMSO-*d*_6_) *δ* 201.7,153.8, 149.3, 146.8, 137.7, 134.3, 131.1, 128.6, 127.9, 127.5, 119.0, 110.6, 50.1, 21.5. MS (ESI): 261.08 [M + H]^+^.

*1-(4-Amino-2-chloropyridin-3-yl)-2-(4-ethylphenyl)ethan-1-one* (**17j**) was prepared from **15** and (4-ethylbenzyl)magnesium chloride (**16*J***) by general procedure I as a white solid (309 mg, 75%), M.p. 89–102 °C, ^1^H-NMR (600 MHz, DMSO-*d*_6_) *δ* 7.80 (d, *J* = 6.0 Hz, 1H), 7.20–7.11 (m, 4H), 6.60 (d, *J* = 6.0 Hz, 3H), 4.14 (s, 2H), 2.57 (q, *J* = 7.2 Hz, 2H), 1.16 (t, *J* = 7.2 Hz, 3H). ^13^C-NMR (150 MHz, DMSO-*d*_6_) *δ* 201.8, 153.88, 149.1, 146.7, 142.7, 131.6, 130.3, 128.1, 119.0, 110.6, 49.7, 28.4, 16.2. MS (ESI): 275.09 [M + H]^+^.

*1-(4-Amino-2-chloropyridin-3-yl)-2-(4-isopropylphenyl)ethan-1-one* (**17k**) was prepared from **15** and (4-isopropylbenzyl)magnesium chloride (**16k**) by general procedure I as a white solid (307.5 mg, 71%), M.p. 71–75 °C, ^1^H-NMR (600 MHz, DMSO-*d*_6_) *δ* 7.81 (t, *J* = 5.2 Hz, 1H), 7.22–7.15 (m, 4H), 6.62 (t, *J* = 4.4 Hz, 3H), 4.14 (s, 1H), 2.85 (p, *J* = 6.0 Hz, 1H), 1.27–1.14 (m, 6H). ^13^C-NMR (150 MHz, DMSO-*d*_6_) *δ* 201.8, 153.9, 149.1, 147.3, 146.7, 131.7, 130.4, 126.6, 119.1, 110.6, 49.7, 33.6, 24.5. MS (ESI): 289.11 [M + H]^+^.

*1-(4-Amino-2-chloropyridin-3-yl)-2-(naphthalen-1-yl)ethan-1-one* (**17l**) was prepared from **15** and (naphthalen-1-ylmethyl)magnesium chloride (**16l**) by general procedure I as a white solid (297.6 mg, 67%), M.p. 78–80 °C, ^1^H-NMR (400 MHz, DMSO-*d*_6_) *δ* 7.87–7.86 (m, 1H), 7.85–7.84 (m, 1H), 7.83–7.81 (m, 2H), 7.53–7.50 (m, 2H), 7.48–7.46 (m, 2H), 6.66 (s, 2H), 6.65 (d, *J* = 6.0 Hz, 1H), 4.70 (s, 2H). ^13^C-NMR (100 MHz, DMSO-*d*_6_) *δ* 201.3, 154.2, 149.0, 147.1, 133.7, 132.6, 131.1, 129.1, 128.8, 128.0, 126.5, 126.1, 125.9, 124.8, 118.6, 110.8, 47.7. MS (ESI): 297.07 [M + H]^+^.

*1-(4-Amino-2-chloropyridin-3-yl)-3-phenylpropan-1-one* (**17m**) was prepared from **15** and phenethyl- magnesium chloride (**16m**) by general procedure I as a white solid (320.7 mg, 82%), M.p. 81–83 °C, ^1^H-NMR (400 MHz, DMSO-*d*_6_) *δ* 7.80 (d, *J* = 6.0 Hz, 1H), 7.41–7.08 (m, 5H), 6.61 (d, *J* = 6.0 Hz, 1H), 6.58 (s, 2H), 3.15 (t, *J* = 7.6 Hz, 2H), 2.94 (t, *J* = 7.6 Hz, 2H). ^13^C-NMR (100 MHz, DMSO-*d*_6_) *δ* 203.2, 153.8, 149.1, 146.8, 141.1, 128.8, 128.7, 126.4, 118.8, 110.5, 45.3, 29.8.MS (ESI): 261.91 [M + H]^+^.

*1-(4-Amino-2-chloropyridin-3-yl)propan-1-one* (**17n**) was prepared from **15** and ethylmagnesium chloride (**16n**) by general procedure I as a white solid (210 mg, 75%), M.p. 97–100 °C, ^1^H-NMR (400 MHz, DMSO-*d*_6_) *δ* 7.80 (d, *J* = 6.0 Hz, 1H), 6.61 (d, *J* = 6.0 Hz, 1H), 6.50 (s, 2H), 2.82 (q, *J* = 7.2 Hz, 2H), 1.09 (t, *J* = 7.2 Hz, 3H). ^13^C-NMR (100 MHz, DMSO-*d*_6_) *δ* 204.7, 153.5, 149.0, 146.5, 119.3, 110.4, 37.1, 8.3. MS (ESI): 185.04 [M + H]^+^.

### 4.3. General Procedure II for the Synthesis of 3-Substituted 1,6-naphthyridin-4-ones ***13a–o***

A solution of 2-substituted 1-(4-amino-2-chloropyridin-3-yl)-ethan-1-one **17** (0.5 mmol) and DMAP (0.5 mmol) in triethyl orthoformate (**18a**, for **13a**–**n**) or triethyl orthoacetate (**18b**, for **13o**) (1.0 mL) was heated to 110 °C and stirred for 24 h, then cooled to room temperature, the resulted solid was filtered and the filter residue was added to a 5 mL round-bottomed flask charged with 2.0 mL isopropanol, refluxed for 2 h and filtered immediately to afford the corresponding compound **13** in good yield.

*5-Chloro-3-(4-fluorophenyl)-1,6-naphthyridin-4-one* (**13a**) was prepared from **17a** and triethyl orthoformate (**18a**) by general procedure II as a light yellow solid (98 mg, 72%), M.p. 296–299 °C, ^1^H-NMR (400 MHz, DMSO-*d*_6_) *δ* 12.3 (s, 1H), 8.31 (d, *J* = 5.6 Hz, 1H), 8.15 (s, 1H), 7.69–7.65 (m, 2H), 7.45 (d, *J* = 5.6 Hz, 1H), 7.25–7.20 (m, 2H). ^13^C-NMR (100 MHz, DMSO-*d*_6_) *δ* 173.8, 161.8 (d, *J_C_*_–*F*_ = 243.0 Hz), 150.6, 148.2, 147.7, 138.2, 131.8 (d, *J_C_*_–*F*_ = 3.0 Hz), 131.1 (d, *J_C_*_–*F*_ = 7.0 Hz), 124.5, 118.0, 115.1 (d, *J_C_*_–*F*_ = 21.0 Hz), 113.1. MS (ESI): 275.21 [M + H]^+^.

*5-Chloro-3-(2-fluorophenyl)-1,6-naphthyridin-4(1H)-one* (**13b**) was prepared from **17b** and triethyl orthoformate (**18a**) by general procedure II as a light yellow solid (63 mg, 46%), M.p. 264–268 °C, ^1^H-NMR (400 MHz, DMSO-*d*_6_) *δ* 12.38 (s, 1H), 8.33 (d, *J* = 6.0 Hz, 1H), 8.11 (s, 1H), 7.54–7.34 (m, 3H), 7.32–7.14 (m, 2H). ^13^C-NMR (150 MHz, DMSO-*d*_6_) *δ* 173.2, 160.7 (d, *J_C_*_–*F*_ = 244.5 Hz), 150.4, 148.4, 147.9, 139.2, 132.6, 129.9 (d, *J_C_*_–*F*_ = 7.5 Hz), 124.4, 123.3 (d, *J_C_*_–*F*_ = 13.5 Hz), 121.3, 117.6, 115.7 (d, *J_C_*_–*F*_ = 22.5 Hz), 113.1. MS (ESI): 275.23 [M + H]^+^.

*5-Chloro-3-(3-fluorophenyl)-1,6-naphthyridin-4(1H)-one* (**13c**) was prepared from **17c** and triethyl orthoformate (**18a**) by general procedure II as a light yellow solid (87.9 mg, 64%), M.p. 273–275 °C, ^1^H-NMR (400 MHz, DMSO-*d*_6_) *δ* 12.41 (s, 1H), 8.33 (d, *J* = 6.0 Hz, 1H), 8.25 (s, 1H), 7.59–7.38 (m, 4H), 7.20–7.10 (m, 1H). ^13^C-NMR (150 MHz, DMSO-*d*_6_) *δ* 173.7, 162.3 (d, *J_C_*_–*F*_ = 244.5 Hz), 150.7, 148.4, 147.7 (d, *J_C_*_–*F*_ = 4.5 Hz), 138.9, 137.9, 130.2, 125.0, 123.9 (d, *J_C_*_–*F*_ = 4.5 Hz), 118.1 (d, *J_C_*_–*F*_ = 4.5 Hz), 115.8 (d, *J_C_*_–*F*_ = 22.5 Hz), 114.2 (d, *J_C_*_–*F*_ = 13.5 Hz), 113.15, MS (ESI): 275.03 [M + H]^+^.

*5-Chloro-3-(2,4-difluorophenyl)-1,6-naphthyridin-4(1H)-one* (**13d**) was prepared from **17d** and triethyl orthoformate (**18a**) by general procedure II as a light yellow solid (58.5 mg, 40%), M.p. 238–241 °C, ^1^H-NMR (400 MHz, DMSO-*d*_6_) *δ* 12.58 (s, 1H), 8.89 (s, 1H), 7.93 (d, *J* = 6.0 Hz, 1H), 7.68–7.62 (m, 1H), 7.46 (td, *J*_1_ = 9.6 Hz, *J*_2_ = 2.4 Hz, 1H), 7.28 (td, *J_1_* = 8.4 Hz, *J*_2_ = 2.4 Hz, 1H), 7.05 (d, *J* = 6.0 Hz, 1H). ^13^C-NMR (100 MHz, DMSO-*d*_6_) *δ* 171.6, 165.9 (dd, *J_C_*_–*F*_ = 298.0 Hz, 12.0 Hz), 163.0 (dd, *J_C_*_–*F*_ = 298.0 Hz, 12.0 Hz), 160.6, 149.3, 148.2, 139.0, 133.8 (dd, *J_C_*_–*F*_ = 10.0 Hz, 4.0 Hz), 116.5, 116.1 (dd, *J_C_*_–*F*_ = 15.0 Hz, 4.0 Hz), 112.4, (dd, *J_C_*_–*F*_ = 21.0 Hz, 4.0 Hz), 109.1, 104.9 (t, *J_C_*_–*F*_ = 21.0 Hz), 101.7. MS (ESI): 293.42 [M + H]^+^.

*5-Chloro-3-(4-chlorophenyl)-1,6-naphthyridin-4(1H)-one* (**13e**) was prepared from **17e** and triethyl orthoformate (**18a**) by general procedure II as a light yellow solid (88.5 mg, 61%), M.p. 196–200 °C, ^1^H-NMR (400 MHz, DMSO-*d*_6_) *δ* 12.60 (s, 1H), 8.32 (d, *J* = 6.0 Hz, 1H), 8.19 (s, 1H), 7.68 (d, *J* = 8.4 Hz, 2H), 7.49 (d, *J* = 6.0 Hz, 1H), 7.45 (d, *J* = 8.4 Hz, 2H). ^13^C-NMR (100 MHz, DMSO-*d*_6_) *δ* 173.7, 150.6, 148.2, 147.6, 138.4, 134.3, 132.0, 130.8, 128.3, 124.0, 118.0, 113.1. MS (ESI): 292.11 [M + H]^+^.

*3-(4-Bromophenyl)-5-chloro-1,6-naphthyridin-4(1H)-one* (**13f**) was prepared from **17f** and triethyl ortho- formate (**18a**) by general procedure II as a light yellow solid (85.6 mg, 51%), M.p. 239–241 °C, ^1^H-NMR (400 MHz, DMSO-*d*_6_) *δ* 12.36 (s, 1H), 8.32 (d, *J* = 6.0 Hz, 1H), 8.18 (s, 1H), 7.64–7.58 (m, 4H), 7.45 (d, *J* = 6.0 Hz, 1H). ^13^C-NMR (100 M, DMSO-*d*_6_) *δ* 173.6, 150.6, 148.2, 147.6, 138.3, 134.7, 131.2, 131.1, 124.1, 120.6, 118.0, 113.0. MS (ESI): 336.95 [M + H]^+^.

*5-Chloro-3-phenyl-1,6-naphthyridin-4(1H)-one* (**13g**) was prepared from **17g** and triethyl orthoformate (**18a**) by general procedure II as a light yellow solid (89.8 mg, 69%), M.p. 198–200 °C, ^1^H-NMR (400 MHz, DMSO-*d*_6_) *δ*12.22 (s, 1H), 8.21 (d, *J* = 6.0 Hz, 1H), 8.03 (d, *J* = 5.6 Hz, 1H), 7.53 (d, *J* = 1.6 Hz, 1H), 7.52 (s, 1H), 7.38–7.18 (m, 4H). ^13^C-NMR (100 MHz, DMSO-*d*_6_) *δ* 173.9, 150.7, 148.1, 147.7, 138.1, 135.5, 129.2, 128.3, 127.5, 125.6, 118.0, 113.0. MS (ESI): 292.11 [M + H]^+^.

*5-Chloro-3-(p-tolyl)-1,6-naphthyridin-4(1H)-one* (**13h**) was prepared from **17h** and triethyl ortho- formate (**18a**) by general procedure II as a light yellow solid (110.9 mg, 82%), M.p. 222–225 °C, ^1^H-NMR (400 MHz, DMSO-*d*_6_) *δ* 12.28 (s, 1H), 8.30 (d, *J* = 6.0 Hz, 1H), 8.09 (s, 1H), 7.53 (d, *J* = 8.4 Hz, 2H), 7.46 (d, *J* = 6.0 Hz, 1H), 7.20 (d, *J* = 8.4 Hz, 2H), 2.33 (s, 3H). ^13^C-NMR (100 MHz, DMSO-*d*_6_) *δ*173.9, 150.6, 148.0, 147.5, 137.7, 136.6, 132.5, 128.9, 128.8, 125.4, 117.9, 113.0, 21.2. MS (ESI): 271.85[M + H]^+^.

*5-Chloro-3-(m-tolyl)-1,6-naphthyridin-4(1H)-one* (**13i**) was prepared from **17i** and triethyl orthoformate (**18a**) by general procedure II as a light yellow solid (83.9 mg, 62%), M.p. 257–261 °C, ^1^H-NMR (600 MHz, DMSO-*d*_6_) *δ* 12.32 (s, 1H), 8.31 (d, *J* = 6.0 Hz, 1H), 8.12 (s, 1H), 7.46 –7.44 (m, 2H), 7.40 (d, *J* = 6.0 Hz, 1H), 7.30 (t, *J* = 7.6 Hz, 1H), 7.14 (d, *J* = 7.6 Hz, 1H), 2.35 (s, 3H). ^13^C-NMR (150 MHz, DMSO-*d*_6_) *δ* 173.9, 150.7, 148.2, 147.7, 138.1, 137.3, 135.5, 129.8, 128.3, 128.2, 126.3, 125.7, 118.0, 113.1, 21.7. MS (ESI): 271.85[M + H]^+^.

*5-Chloro-3-(4-ethylphenyl)-1,6-naphthyridin-4(1H)-one* (**13j**) was prepared from **17*J*** and triethyl ortho- formate (**18a**) by general procedure II as a light yellow solid (85.4 mg, 60%), M.p. 270–273 °C, ^1^H-NMR (600 MHz, DMSO-*d*_6_) *δ* 12.30 (s, 1H), 8.30 (d, *J* = 6.0 Hz, 1H), 8.11 (s, 1H), 7.57–7.49 (m, 2H), 7.45 (d, *J* = 6.0 Hz, 1H), 7.23 (d, *J* = 7.2 Hz, 2H), 2.62 (q, *J* = 7.2 Hz, 2H), 1.20 (t, *J* = 7.2 Hz, 3H). ^13^C-NMR (150 MHz, DMSO-*d*_6_) *δ* 174.0, 150.7, 148.2, 147.7, 143.2, 137.9, 132.9, 129.2, 127.9, 125.6, 118.0, 113.1, 28.5, 16.3. MS (ESI): 285.65[M + H]^+^.

*5-Chloro-3-(4-isopropylphenyl)-1,6-naphthyridin-4(1H)-one* (**13k**) was prepared from **17k** and triethyl orthoformate (**18a**) by general procedure II as a light yellow solid (91.1 mg, 61%), M.p. 303–304 °C, ^1^H-NMR (600 MHz, DMSO-*d*_6_) 12.39 (s, 1H), 8.42 (d, *J* = 6.0 Hz, 1H), 8.24 (s, 1H), 7.60 (d, *J* = 6.0 Hz, 1H), 7.55 (d, *J* = 8.4 Hz, 2H), 7.28 (d, *J* = 8.4 Hz, 2H), 2.99–2.86 (m, 1H), 1.23 (d, *J* = 7.2 Hz, 6H). ^13^C-NMR (150 MHz, DMSO-*d*_6_) *δ* 174.0, 150.7, 148.1, 147.8, 147.7, 137.9, 133.1, 129.2, 126.3, 125.7, 118.0, 113.1, 33.8, 24.5. MS (ESI): 299.63[M + H]^+^.

*5-Chloro-3-(naphthalen-1-yl)-1,6-naphthyridin-4(1H)-one* (**13l**) was prepared from **17l** and triethyl orthoformate (**18a**) by general procedure II as a light yellow solid (96.6 mg, 63%), M.p. 281–283 °C, ^1^H-NMR (400 MHz, DMSO-*d*_6_) *δ* 12.36 (s, 1H), 8.37 (d, *J* = 5.6 Hz, 1H), 8.12–8.07 (m, 1H), 8.01–7.91 (m, 2H), 7.71–7.63 (m, 1H), 7.62–7.49 (m, 3H), 7.49–7.37 (m, 2H). ^13^C-NMR (100 MHz, DMSO-*d*_6_) *δ* 174.3, 150.6, 148.3, 148.2, 139.2, 134.2, 133.6, 132.5, 128.5, 128.4, 128.3, 126.6, 126.3, 126.2, 125.9, 117.8, 113.2. MS (ESI): 307.91[M + H]^+^.

*3-Benzyl-5-chloro-1,6-naphthyridin-4(1H)-one* (**13m**) was prepared from **17m** and triethyl orthoformate (**18a**) by general procedure II as a light yellow solid (100 mg, 74%), M.p. 212–215 °C, ^1^H-NMR (400 MHz, DMSO-*d*_6_) *δ* 12.00 (s, 1H), 8.26 (d, *J* = 6.0 Hz, 1H), 7.89 (s, 1H), 7.39 (d, *J* = 6.0 Hz, 1H), 7.34–7.21 (m, 4H), 7.21–7.09 (m, 1H), 3.73 (s, 2H). ^13^C-NMR (100 MHz, DMSO-*d*_6_) *δ* 174.7, 150.3, 147.8, 140.9, 137.3, 129.0, 128.6, 126.3, 126.2, 116.9, 113.4, 33.5. MS (ESI): 271.77 [M + H]^+^.

*5-Chloro-3-methyl-1,6-naphthyridin-4(1H)-one* (**13n**) was prepared from **17n** and triethyl orthoformate (**18a**) by general procedure II as a light yellow solid (53.3 mg, 55%), M.p. 181–185 °C, ^1^H-NMR (400 MHz, DMSO-*d*_6_) *δ* 11.94 (s, 1H), 8.23 (d, *J* = 6.0 Hz, 1H), 7.89 (s, 1H), 7.36 (d, *J* = 6.0 Hz, 1H), 1.93 (s, 3H). ^13^C-NMR (100 MHz, DMSO-*d*_6_) *δ* 175.6, 150.2, 147.8, 147.5, 136.4, 122.7, 116.3, 112.9, 14.1. MS (ESI): 285.69 [M + H]^+^.

*5-Chloro-3-(4-fluorophenyl)-2-methyl-1,6-naphthyridin-4(1H)-one* (**13o**) was prepared from **17o** and triethyl orthoacetate (**18b**) by general procedure II as a light yellow solid (99.4 mg, 69%), M.p. 309–311 °C, ^1^H-NMR (600 MHz, DMSO-*d*_6_) *δ* 12.02 (s, 1H), 8.28 (d, *J* = 6.0 Hz, 1H), 7.40 (d, *J* = 6.0 Hz, 1H), 7.30–6.98 (m, 4H), 2.16(s, 3H). ^13^C-NMR (150 MHz, DMSO-*d*_6_) *δ* 173.9, 161.8 (d, *J_C_*_–*F*_ = 241.0 Hz), 150.5, 148.3, 147.6, 147.1, 133.2 (d, *J_C_*_–*F*_ = 7.5 Hz), 131.8 (d, *J_C_*_–*F*_ = 1.5 Hz), 124.9, 116.7, 115.3 (d, *J_C_*_–*F*_ = 21.0 Hz), 112.6, 19.1. MS (ESI): 289.51 [M + H]^+^.

### 4.4. Scale-Up Experiment

#### 4.4.1. Mass Preparation of 1-(4-Amino-2-chloropyridin-3-yl)-2-(4-fluorophenyl)ethan-1-one (**17a**)

To a 5 L four-neck flask 4-amino-2-chloronicotinonitrile **15** (125.0 g, 0.81 mol) and ether (1500 mL) were added, followed by (4-fluorobenzyl)magnesium chloride in ether (**16a**) (1.8 M, 3.24 mol, 1800 mL, 4.0 equivs.) at room temperature. The mixture was warmed to 30 °C and stirred for 12 h under ether atmosphere. Then the reaction was cooled in an ice-water bath with the reaction flask equipped with a distillation unit, and 3000 mL of mixed solvent (HCl/H_2_O/EtOH = 1:1:2) was added dropwise, the volatilized ether was recovered with a distillation unit, and the reaction mixture was refluxed for a further 2 h. After the completion of the reaction, ethanol was removed under vacuum, and the residue was stirred for 12 h and filtered to afford 199 g of crude hydrochloride of **17a** as a yellow solid. Next, the yellow solid was added to a 2.0 L flask charged with ethanol (720 mL) and warmed up to reflux followed by addition of trimethylamine (0.66 mol, 66.86 g) dropwise. The resulted mixture was refluxed for further 1 h, then cooled to room temperature and stirred overnight. Finally, filtered and the filter cake was washed with 50% ethanol in water to afford **17a** as a light yellow solid, (160 g, 75%).

#### 4.4.2. Mass Preparation of 5-Chloro-3-(4-fluorophenyl)-1,6-naphthyridin-4-one (**13a**)

To a 2.0 L of four-neck flask equipped with a distillation unit was added 1-(4-amino-2-chloropyridin-3-yl)-2-(4-fluorophenyl)ethan-1-one (**17a**, 0.604 mol, 160.0 g) and triethyl orthoformate (**18a**, 1600 mL), the reaction mixture was warmed up to 100 °C and stirred for 1.5 h, then DMAP (0.1 eq, 0.6 mol, 7.3 g) was added and the stirring was continued for 24 h at 100 °C with the produced ethanol collected by distillation unit. After the completion of the reaction, the mixture was cooled to room temperature and filtered, the filter cake was added to a 2.0 L of four-neck flask charged with 1500 mL isopropanol, refluxed for 5 h and filtered immediately to afford **13a** as light yellow solid (123.5 g, 74).

## Figures and Tables

**Table 1:** Optimization of the reaction conditions ^a,b^ for the synthesis of 1-(4-amino-2-chloropyridin-3-yl)-2-(4-fluorophenyl)ethan-1-one (**17a**).

Entry	2–5 Equiv.	Solvent	Temp. (°C)	Reaction Time (h)	Yield ^c^ (%)
**1**	2	THF	25	16	5
**2**	2	Et_2_O	25	16	20
**3**	2	Et_2_O	30	12	35
**4**	2	Methyl tert-butyl ether	30	12	10
**5**	2	Isopropyl ether	30	12	7
**6**	2	Toluene	30	12	Trace
**7**	3	Et_2_O	30	12	60
**8**	4	Et_2_O	30	12	74
**9**	5	Et_2_O	30	12	73

^a^ Reactions were performed using 0.5 mmol of 4-amino-2-chloronicotinonitrile (**15**) in 1.0 mL solvent under N_2_ atmosphere. ^b^ 2 mL HCl/H_2_O/EtOH (1:1:2) solvent was added for acidolysis. *^c^* Isolated yield.

**Table 2:** Optimization of the reaction conditions ^a^ for the cyclocondensation.

Entry	Base (equiv.)	Solvent	Temp. (°C)	Reaction Time (h)	Yield ^b^ (%)
1	K_2_CO_3_ (2eq)	DMF	110	24	<1
2	NaH (2eq)	THF	60	24	<1
3	Et_3_N (2eq)	DMF	110	24	15
4	DIPEA (2eq)	DMF	110	24	30
5	DMAP (2eq)	DMF	110	24	65
6	DMAP (2eq)	HC(OEt)_3_	110	24	70
7	DMAP (1eq)	HC(OEt)_3_	110	24	70
8	DMAP (0.1 eq)	HC(OEt)_3_	110	24	72
9	No catalyst	HC(OEt)_3_	110	24	0

^a^ Reactions were performed using 0.5 mmol of 1-(4-amino-2-chloropyridin-3-yl)-2-(4-fluorophenyl)ethan-1-one (**13a**) in 1.0 mL of solvent under an air atmosphere. ^b^ Isolated yields.

## Supplementary Materials

The supplementary materials are available online.
