# Supplementary material for: Grignard Reagent Utilization Enables a Practical and Scalable Construction of 3-Substituted 5-Chloro-1,6-naphthyridin-4-one Derivatives
Source: Molecules. 2020 Dec 1;25(23):5667. doi: 10.3390/molecules25235667 (PMC7730554; doi:10.3390/molecules25235667)
Supplement: Supplementary file 1 [file molecules-25-05667-s001.pdf]

Supporting Information

Utilization of Grignard Reagent Enable the Practical and Scalable Construction of 3- Substituted 5-Chloro-1,6-naphthyridin-4-one Derivatives

Ming-Shu Wang[1,†], Yi Gong[1,†], Zhi-Cheng Yu[1], Yan-Guang Tian[1], Lin-Sheng Zhuo[1,*], Wei Huang[1,*],and Neng-Fang She[1,*]

[1]Key Laboratory of Pesticide & Chemical Biology of Ministry of Education, International Joint Research Center for Intelligent Biosensor Technology and Health, College of Chemistry, Central China Normal University, Wuhan 430079, P.R. China

Table of Contents

I. Preparation of Grignard reagents.. S2

II. ^{1}H and ^{13}C NMR Spectra of synthesized compounds...S3

I. Preparation of Grignard reagents

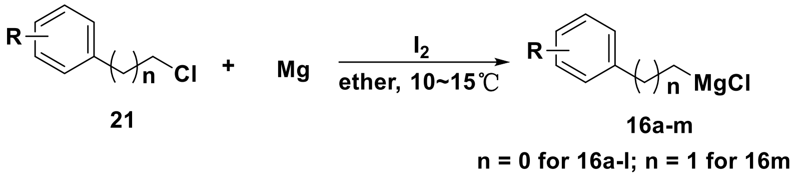

To a 500 mL three-neck flask added metallic magnesium in the form of chips (457.0 mmol, 1.1 equiv), I_2 (2.0 mg) and 150.0 mL ether, then the reaction mixture was warmed to 40°C followed by addition of 7.0 mmol **21**. Next, the reaction mixture was stirred until the color of iodine disappeared, the remaining 388.0 mmol **21** was diluted with 120.0 mL ether and dropped into the reaction solution with the reaction cooled with ice water bath. After the addition was completed, the gray-black mixture was warmed to room temperature and stirred for further 2 h to give desired Grignard reagents with yields of 75-80% (Determined by external standard method using HPLC). Finally, the Grignard reagents was formulated into 1.8 M through adding ether or evaporating ether under reduced pressure (Determined by external standard method using HPLC). **16a** in other solvents were prepared using the same procedure with the ether replaced by corresponding solvents.

II. ^{1}H and ^{13}C NMR Spectra of synthesized compounds

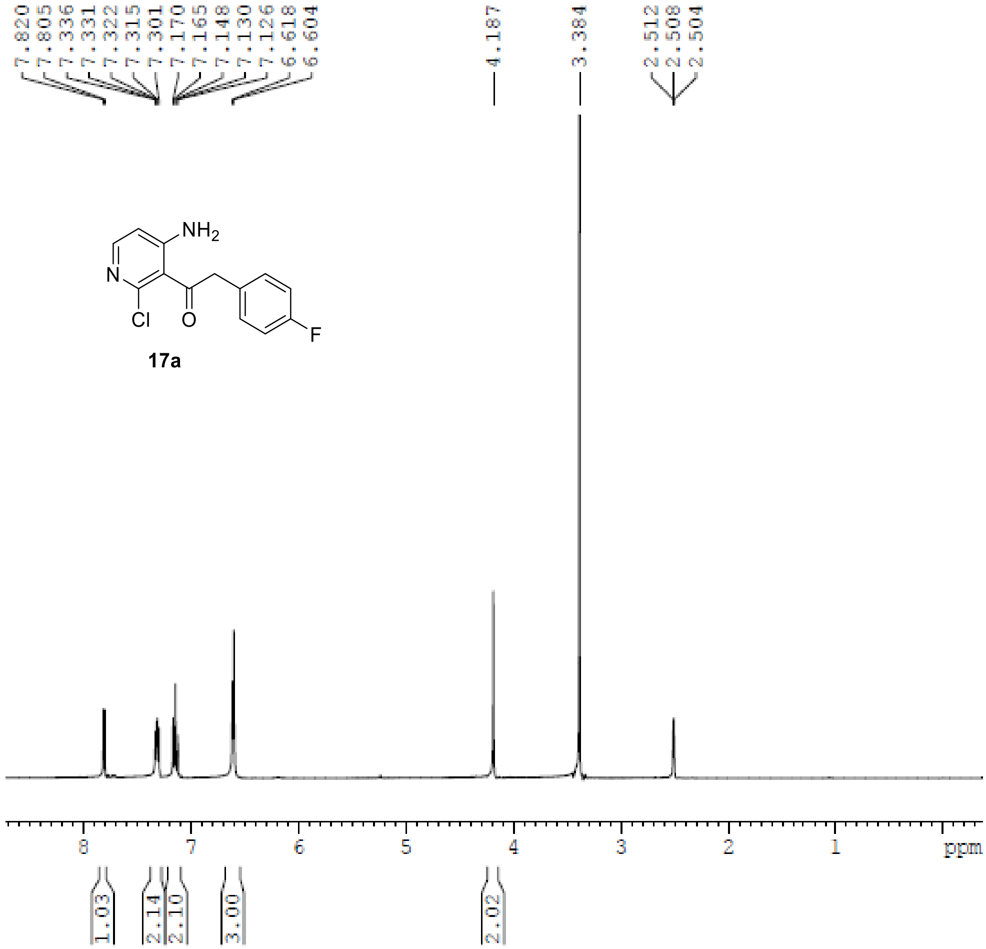

Figure S1: 400 MHz spectrum of ^{1}H-NMR of compound **17a** (DMSO-d_6)

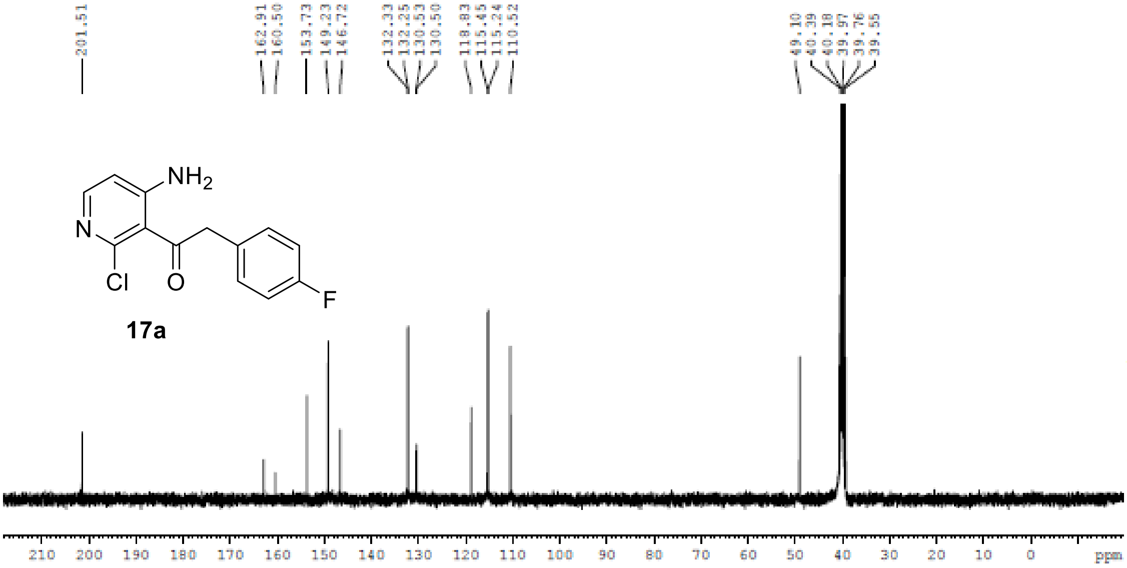

Figure S2: 100 MHz spectrum of ^{13}C-NMR of compound **17a** (DMSO-d_6)

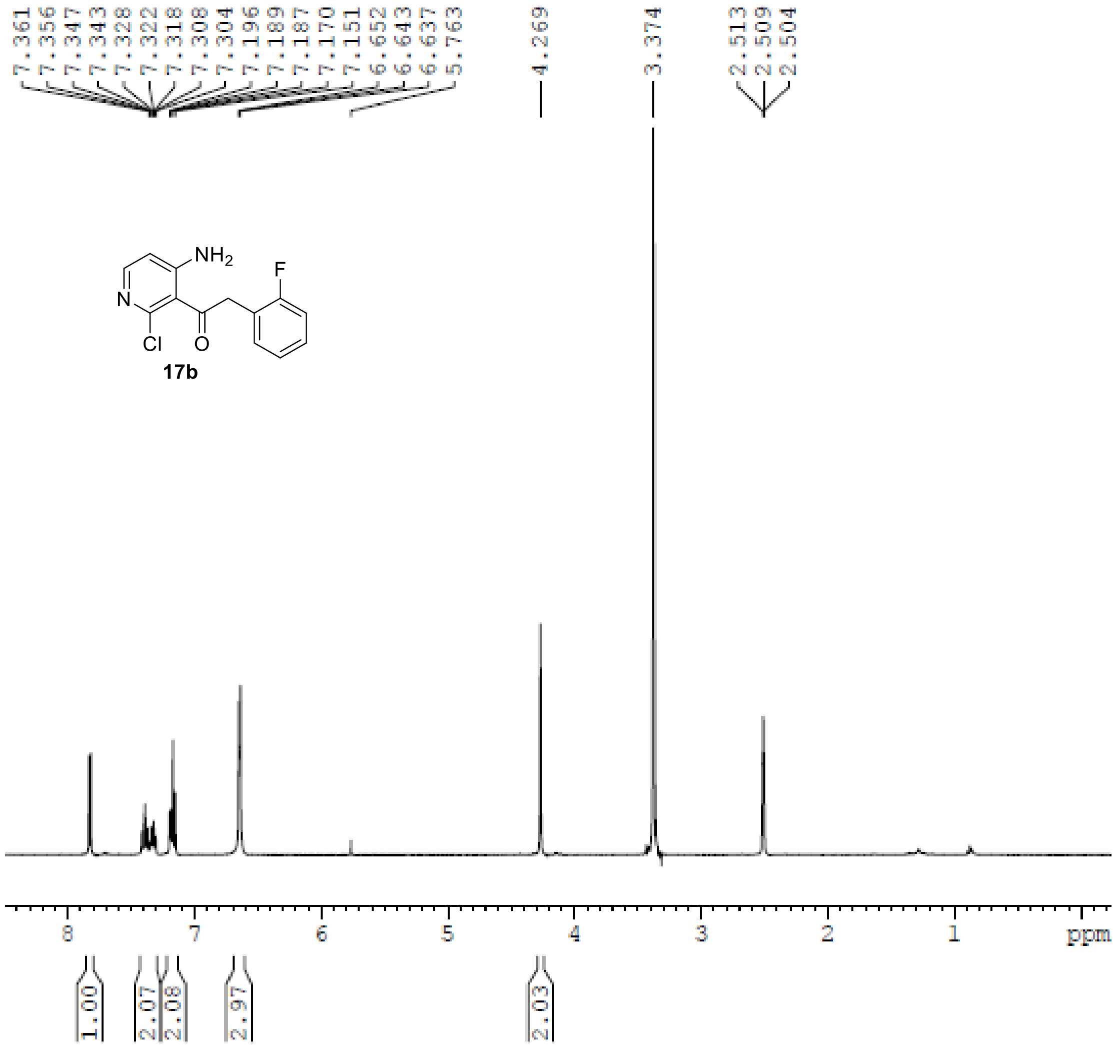

Figure S3: 400 MHz spectrum of ^{1}H-NMR of compound **17b** (DMSO-d_6)

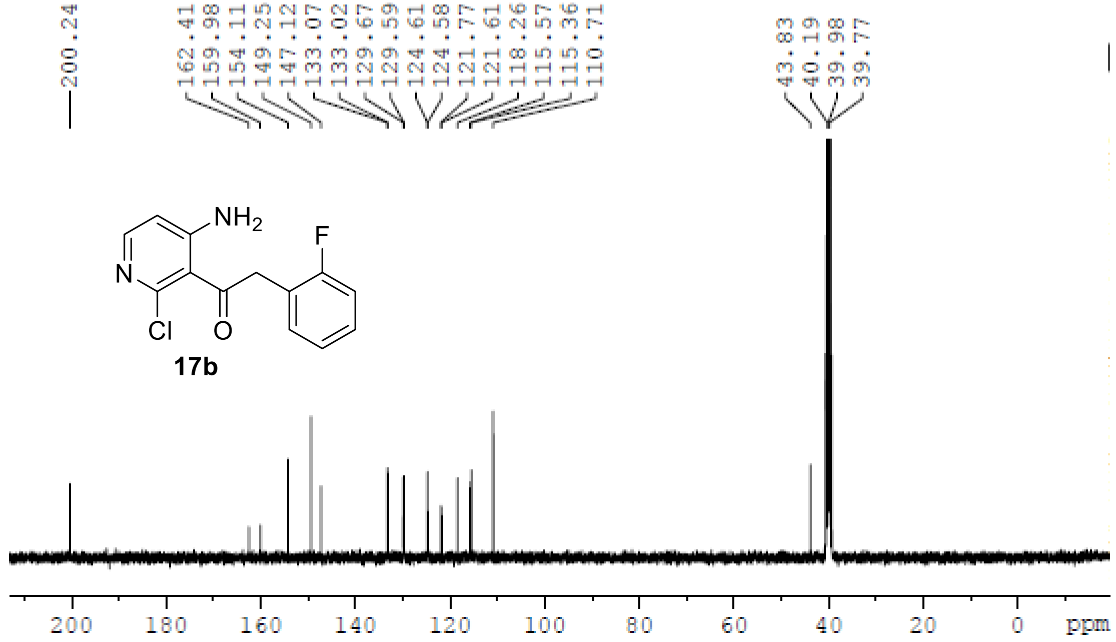

Figure S4: 100 MHz spectrum of ^{13}C-NMR of compound **17b** (DMSO-d_6)

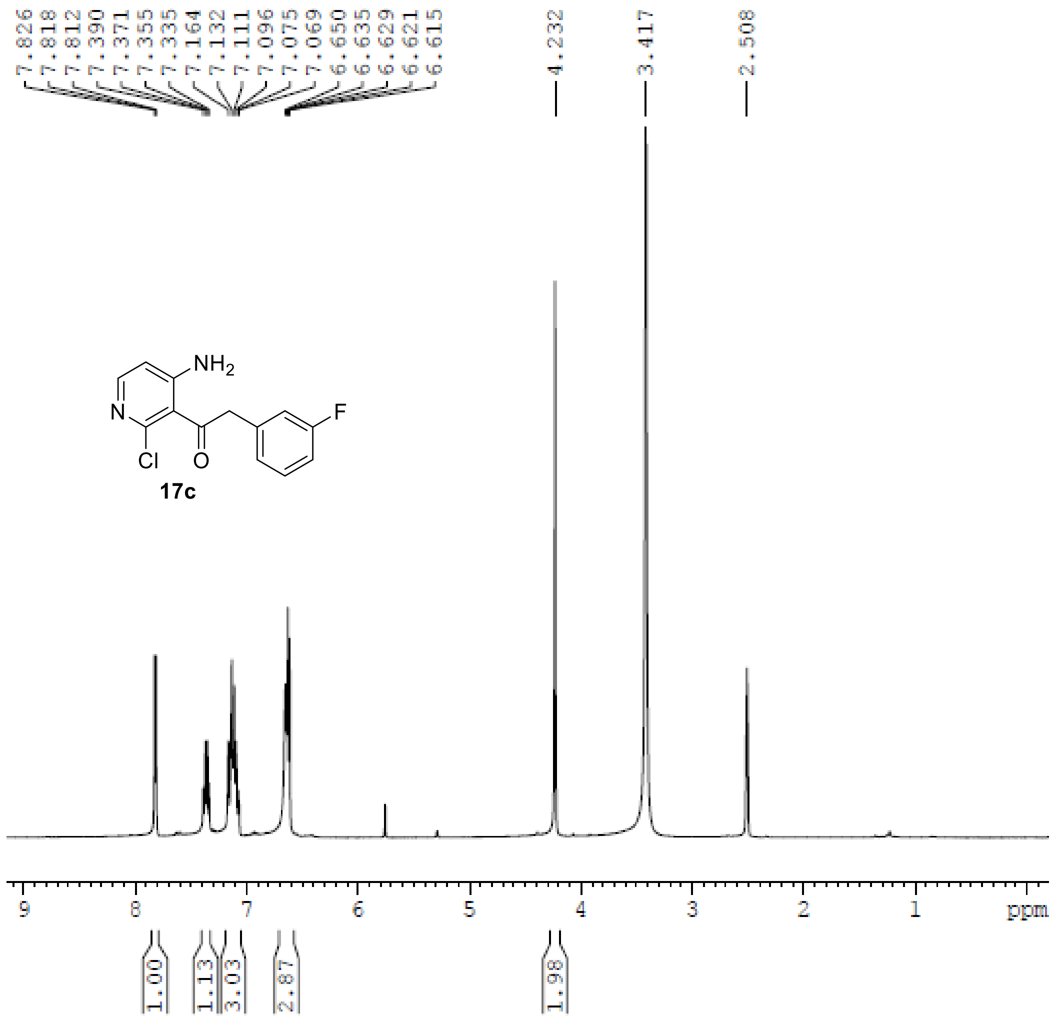

Figure S5: 400 MHz spectrum of ^{1}H-NMR of compound **17c** (DMSO-d_6)

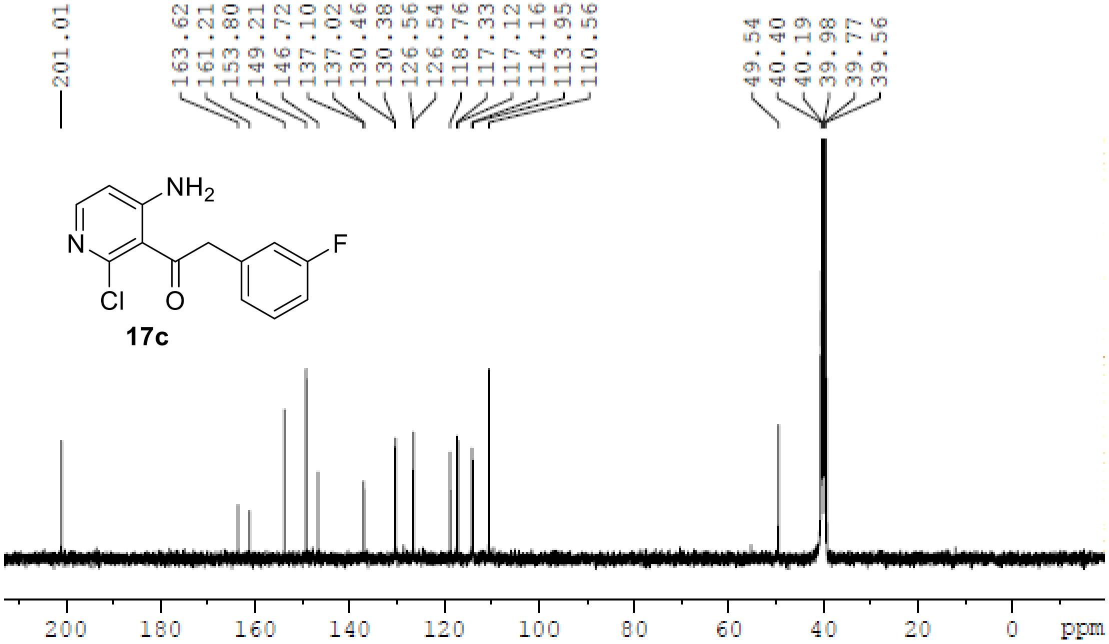

Figure S6: 100 MHz spectrum of ^{13}C-NMR of compound **17c** (DMSO-d_6)

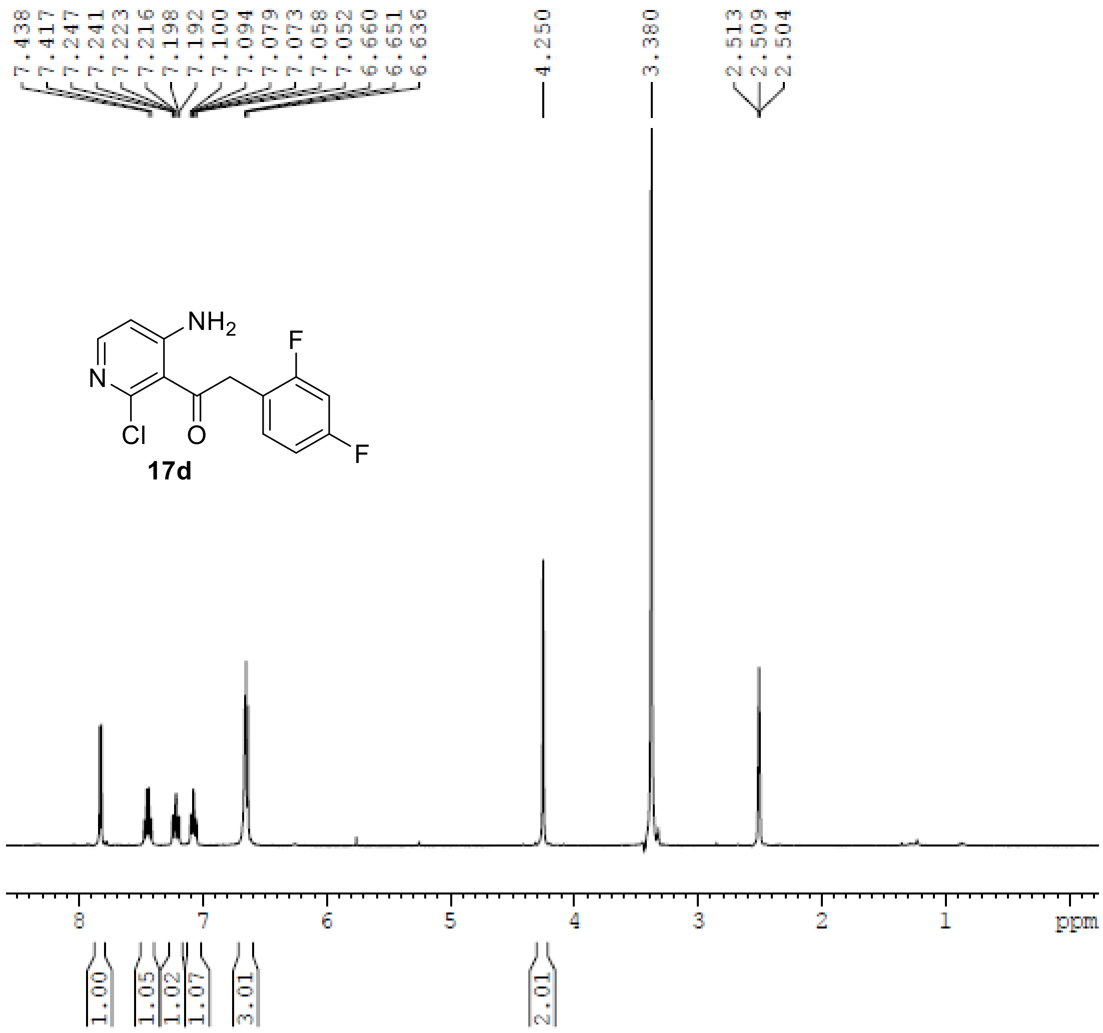

Figure S7: 400 MHz spectrum of ^{1}H-NMR of compound **17d** (DMSO-d_6)

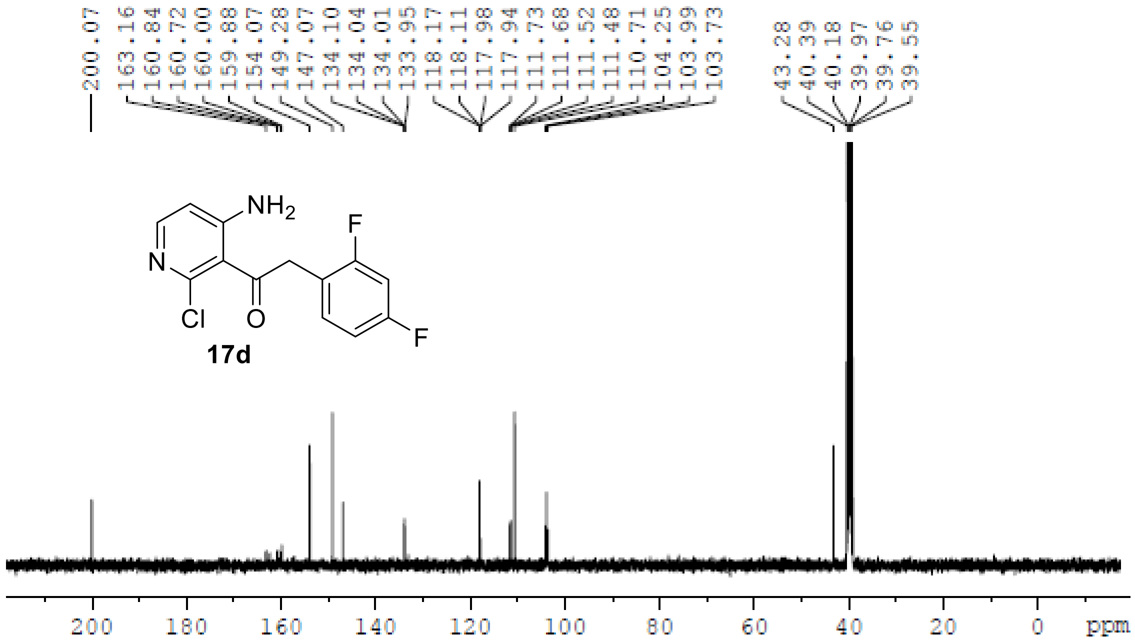

Figure S8: 100 MHz spectrum of ^{13}C-NMR of compound **17d** (DMSO-d_6)

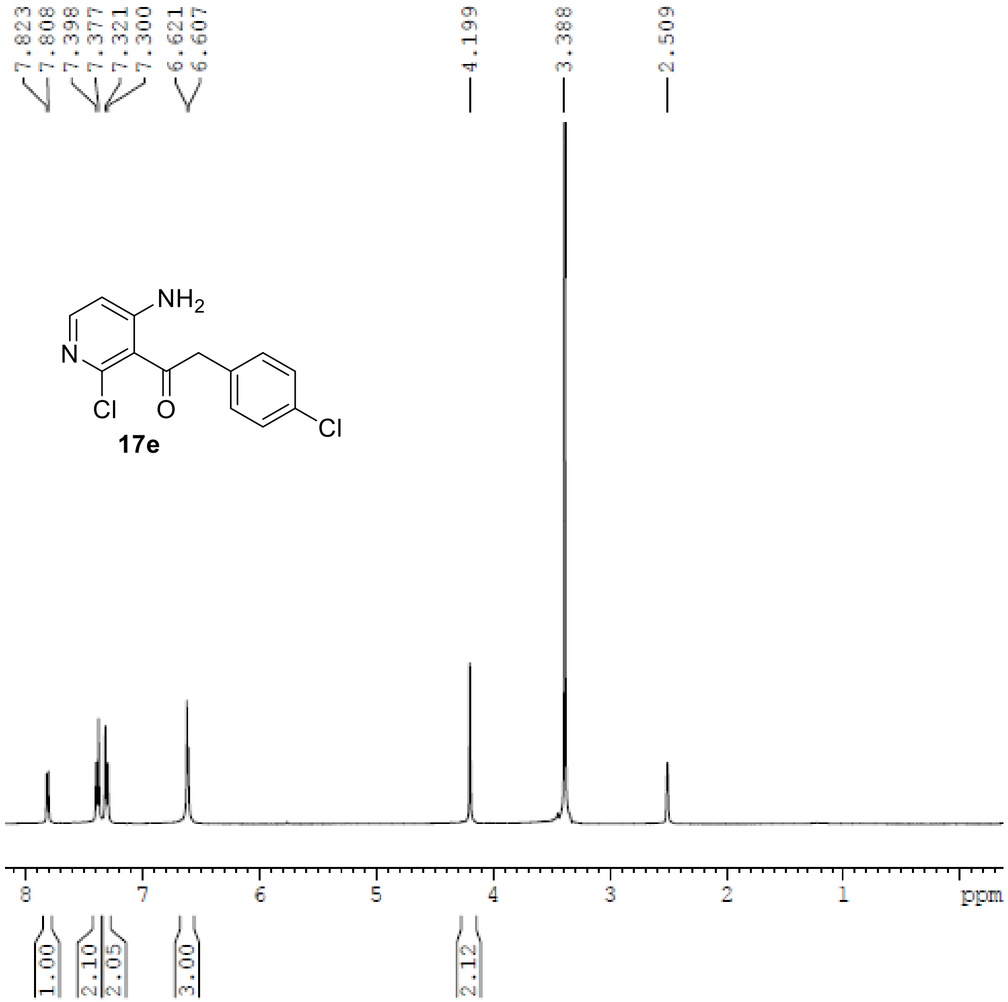

Figure S9: 400 MHz spectrum of ^{1}H-NMR of compound **17e** (DMSO-d_6)

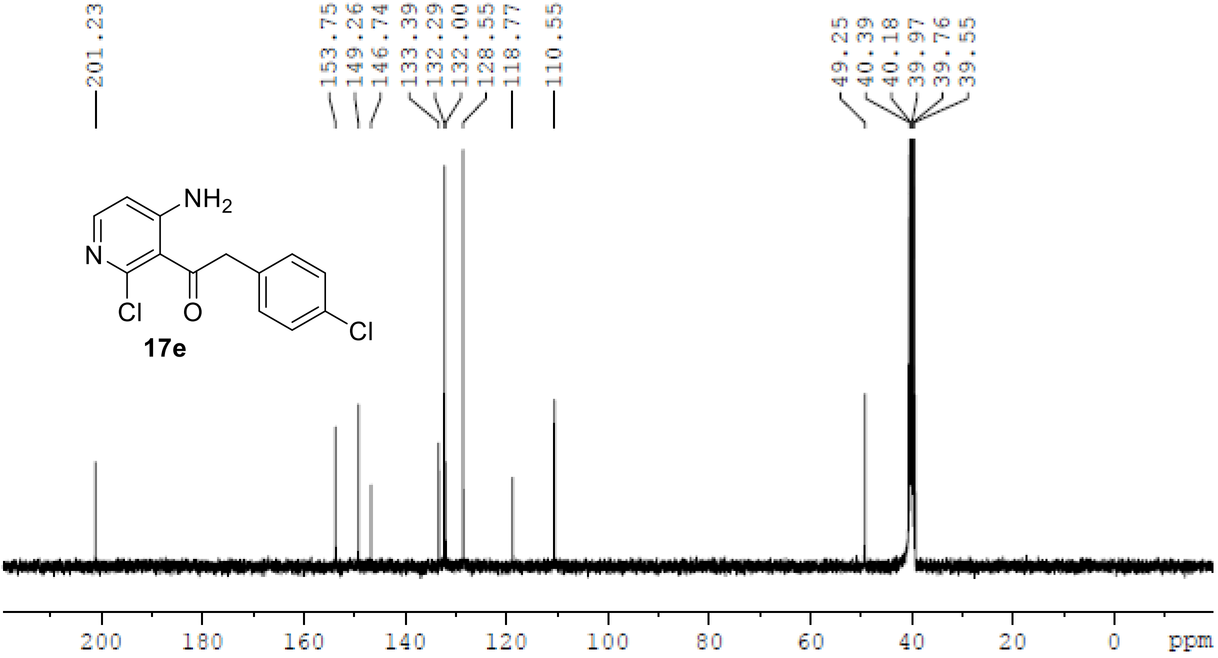

Figure S10: 100 MHz spectrum of ^{13}C-NMR of compound **17e** (DMSO-d_6)

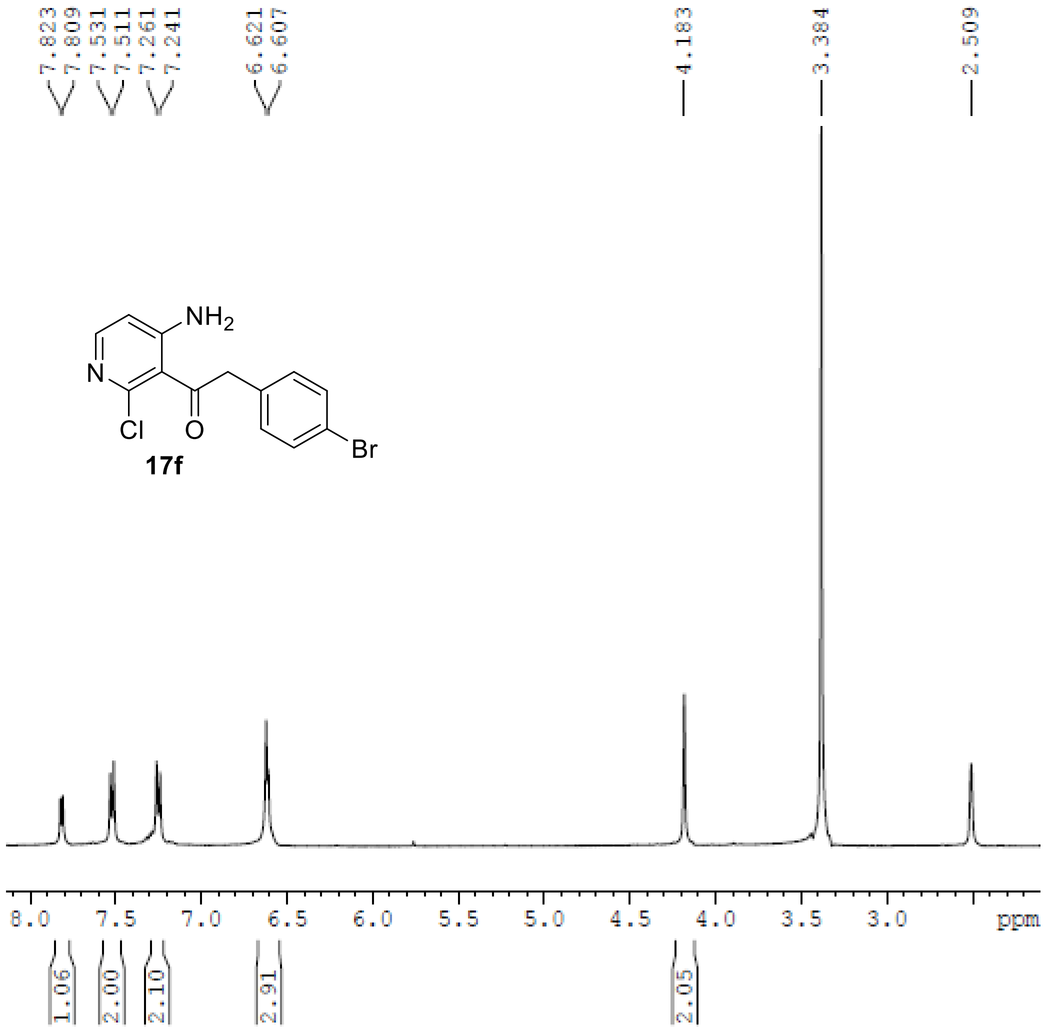

Figure S11: 400 MHz spectrum of ^{1}H-NMR of compound **17f** (DMSO-d_6)

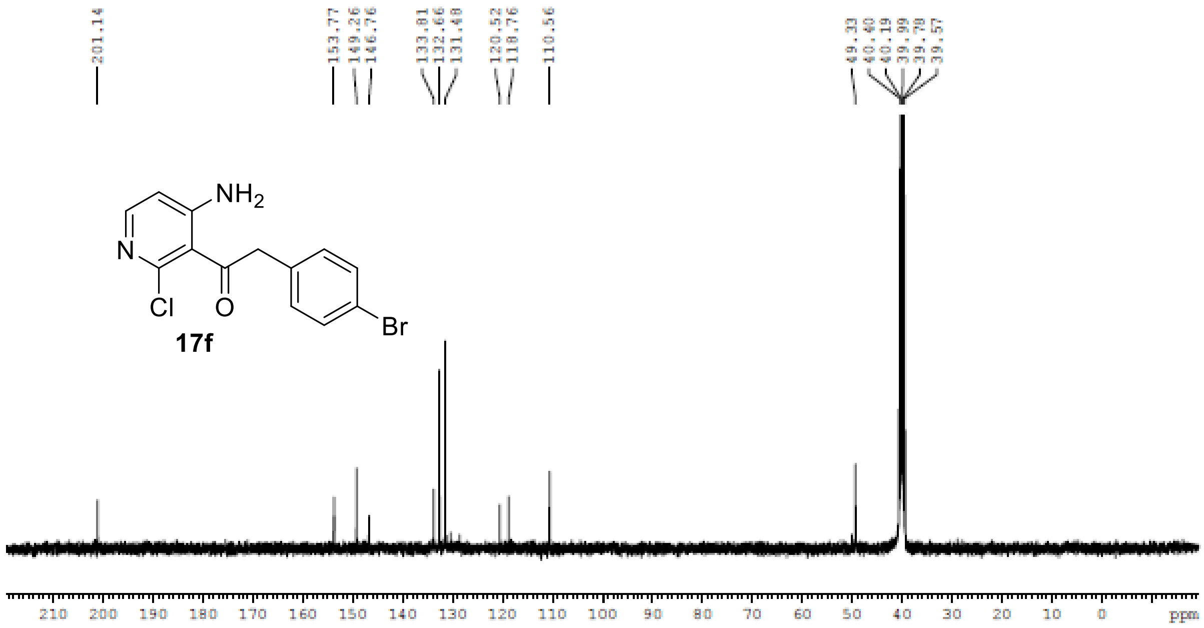

Figure S12: 100 MHz spectrum of ^{13}C-NMR of compound **17f** (DMSO-d_6)

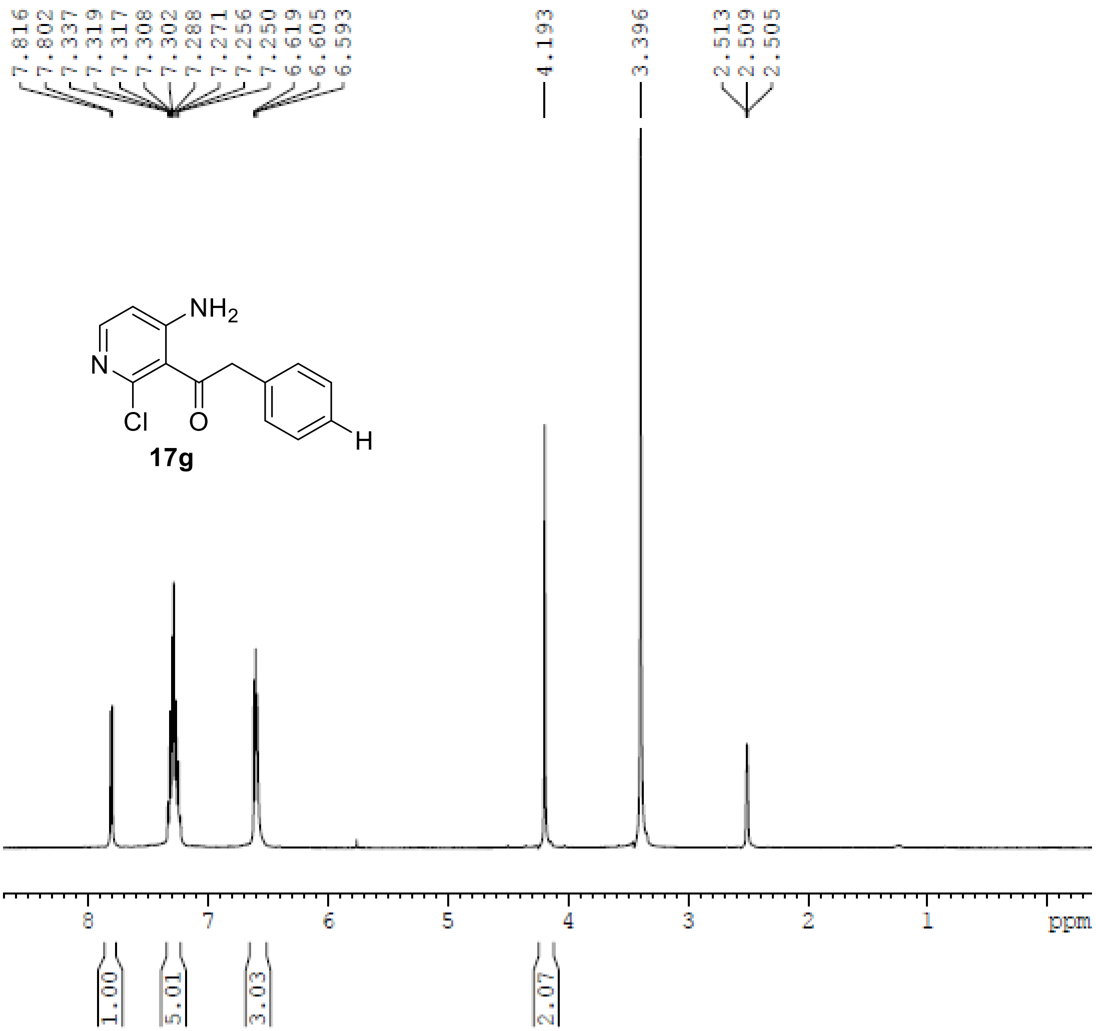

Figure S13: 400 MHz spectrum of ^{1}H-NMR of compound **17g** (DMSO-d_6)

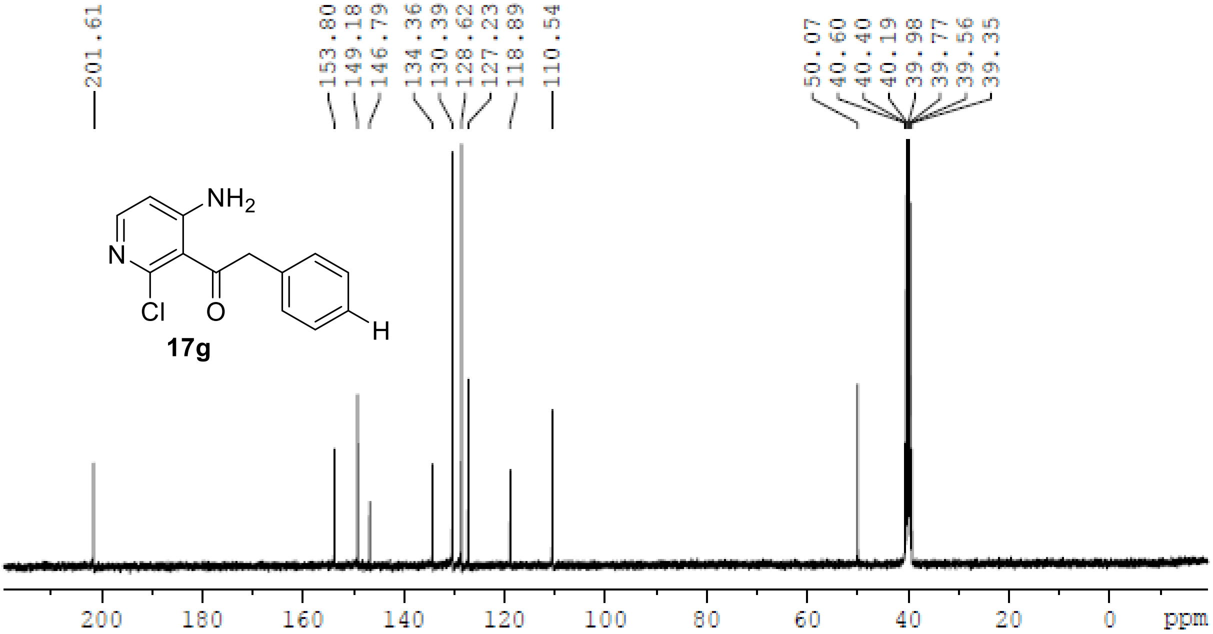

Figure S14: 100 MHz spectrum of ^{13}C-NMR of compound **17g** (DMSO-d_6)

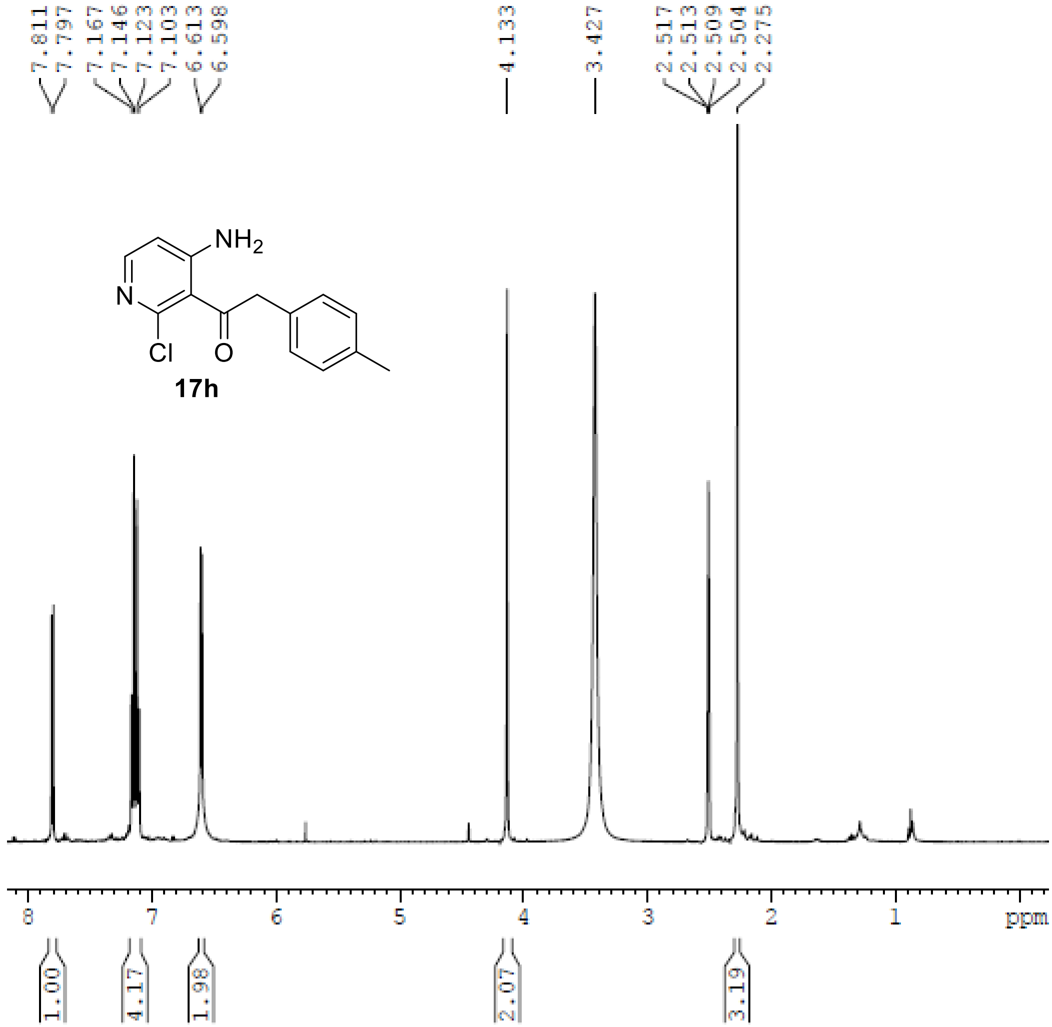

Figure S15: 400 MHz spectrum of ^{1}H-NMR of compound **17h** (DMSO-d_6)

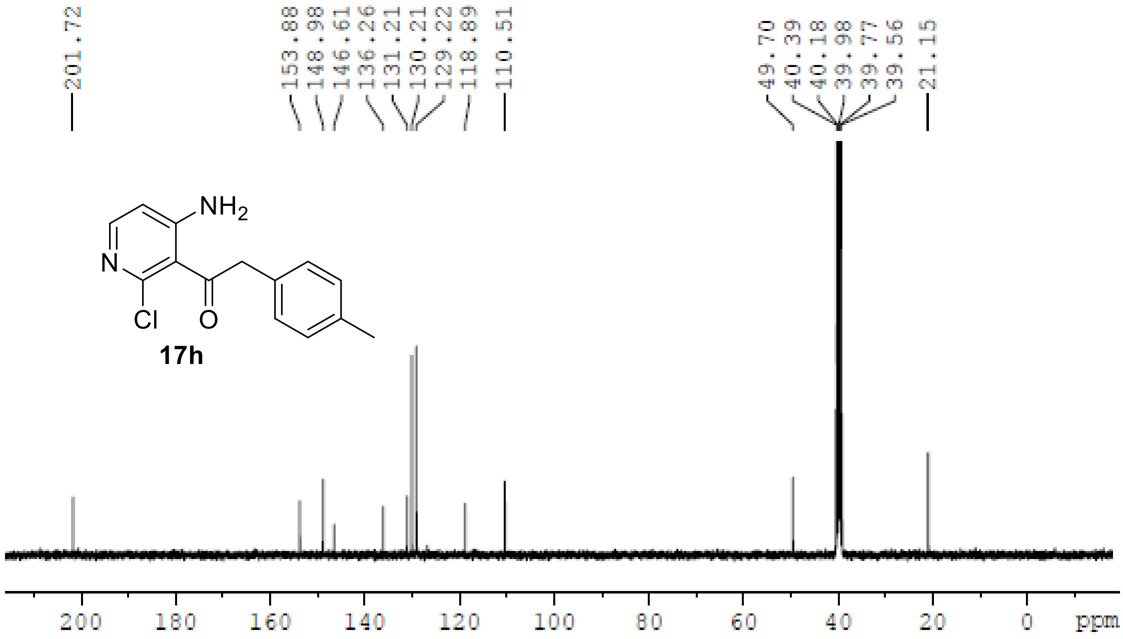

Figure S16: 100 MHz spectrum of ^{13}C-NMR of compound **17h** (DMSO-d_6)

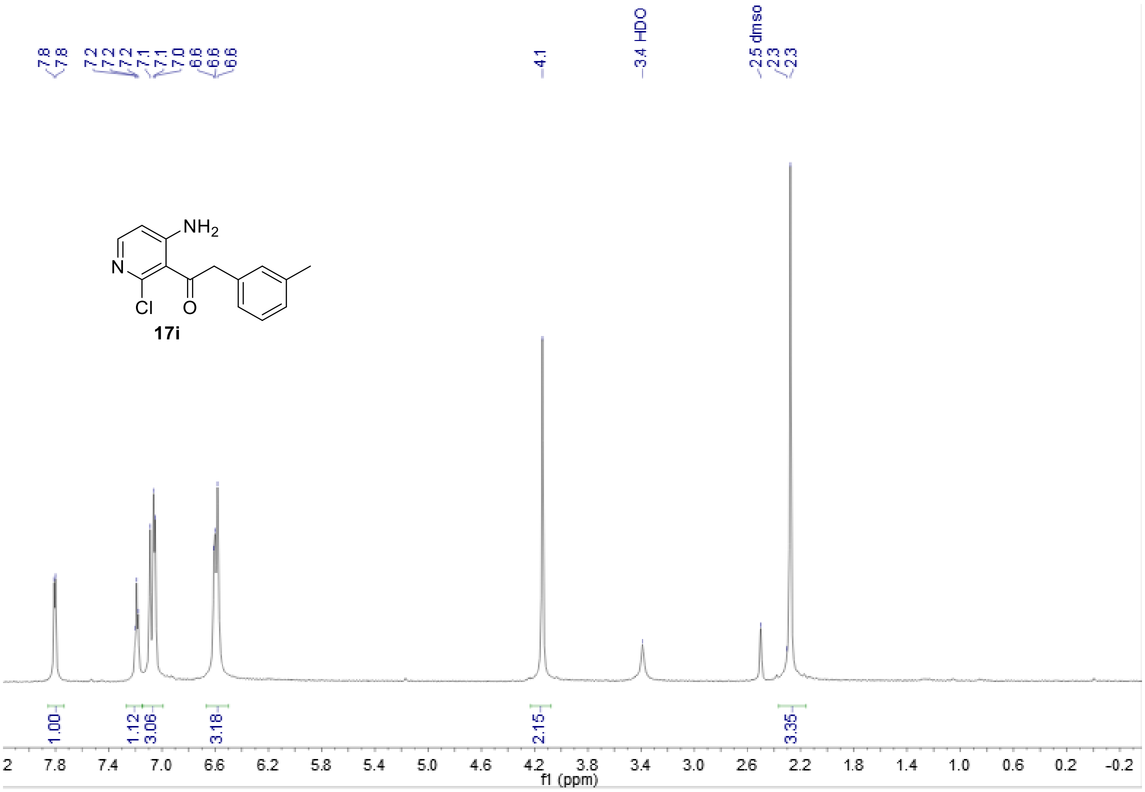

Figure S17: 600 MHz spectrum of ^{1}H-NMR of compound **17i**(DMSO-*d$_6$*)

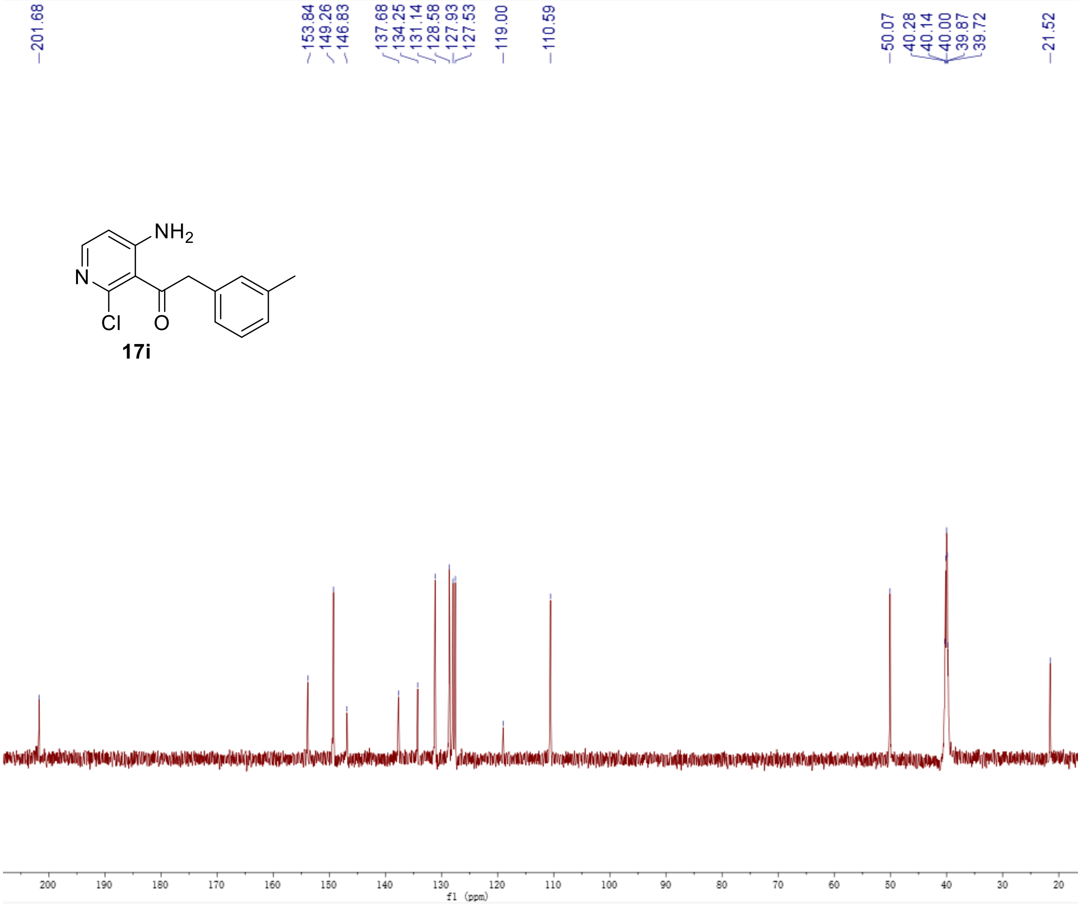

Figure S18: 150 MHz spectrum of ^{13}C-NMR of compound **17i** (DMSO-*d$_6$*)

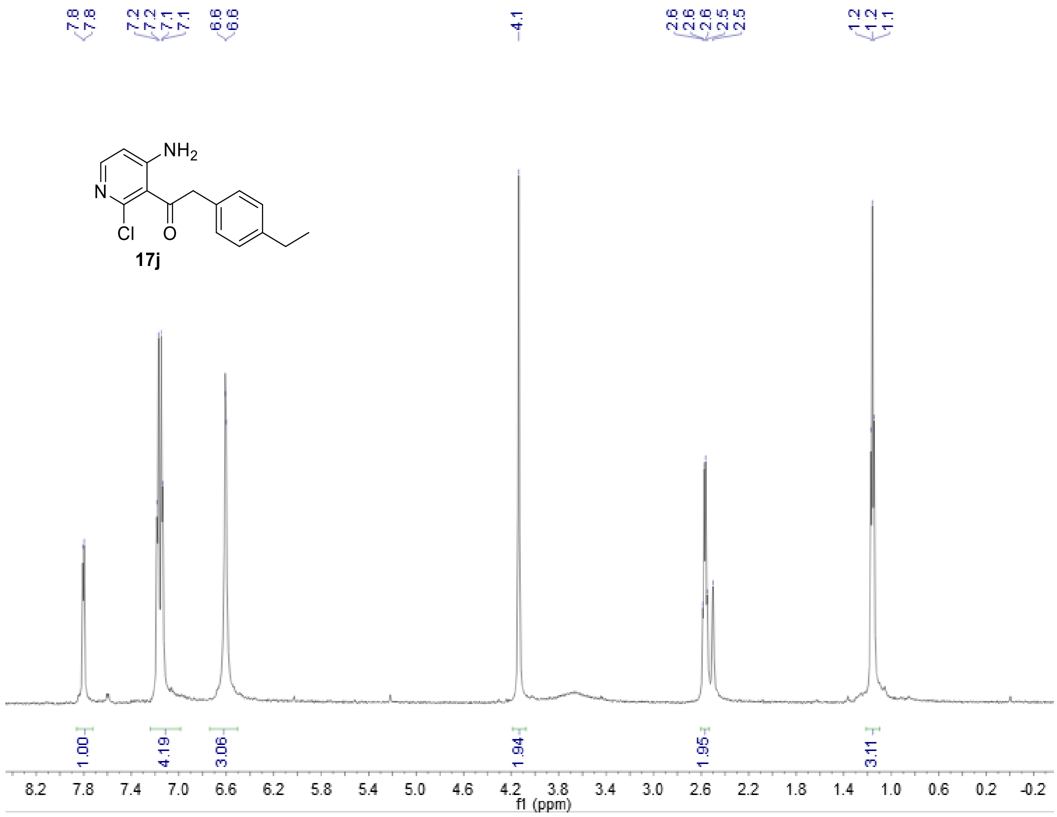

Figure S19: 600 MHz spectrum of ^{1}H-NMR of compound **17j** (DMSO-d_6)

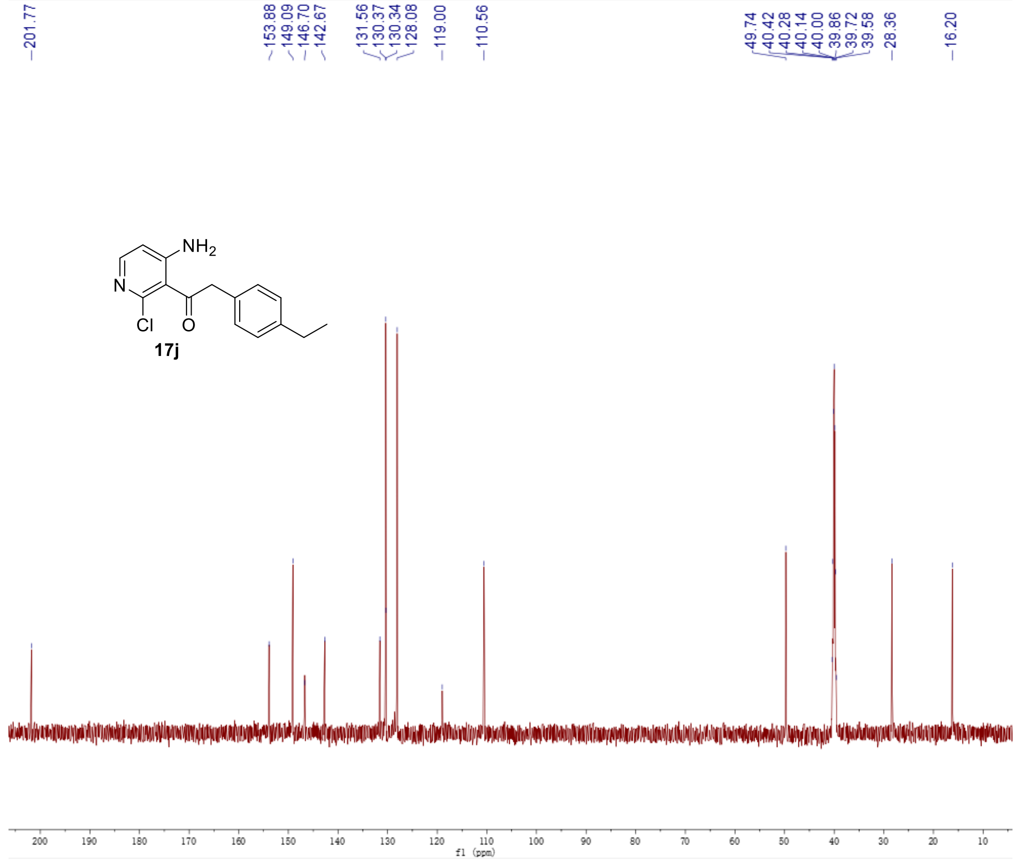

Figure S20: 150 MHz spectrum of ^{13}C-NMR of compound **17j** (DMSO-d_6)

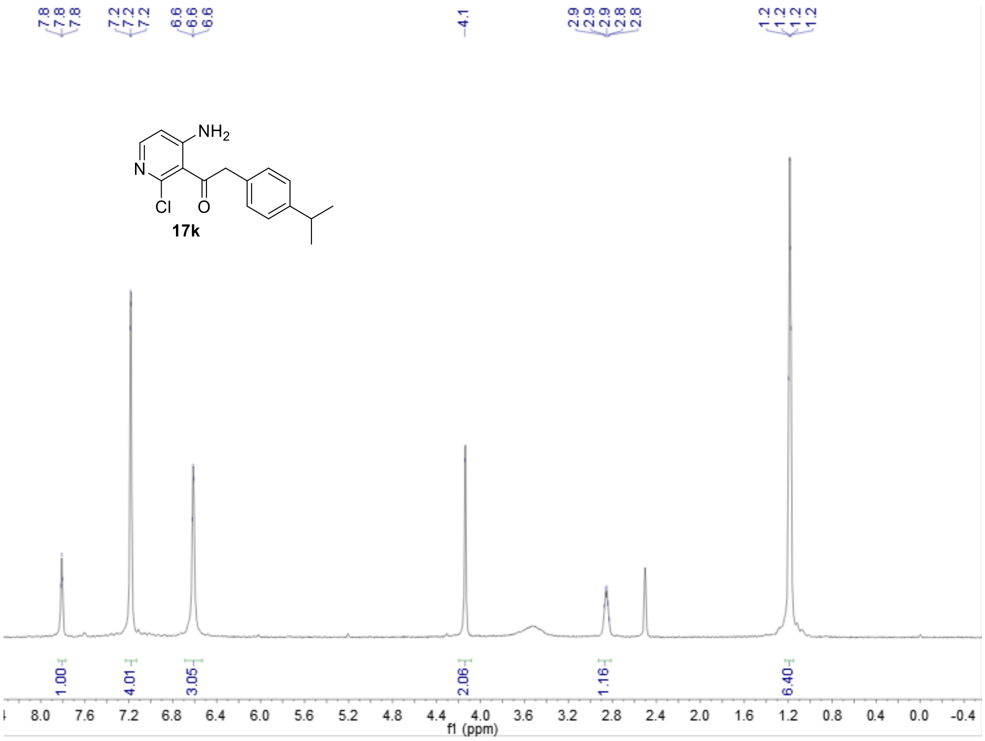

Figure S21: 600 MHz spectrum of ^{1}H-NMR of compound **17k** (DMSO-d_6)

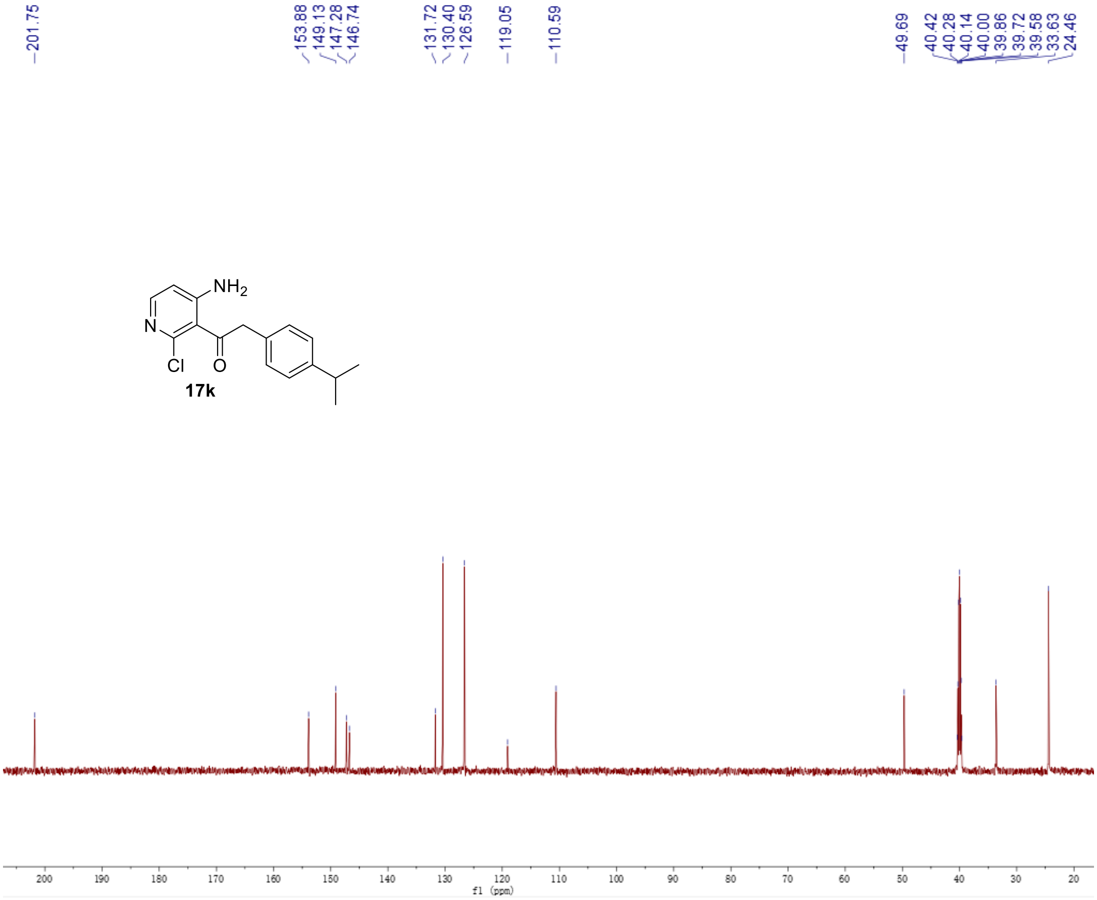

Figure S22: 150 MHz spectrum of ^{13}C-NMR of compound **17k** (DMSO-d_6)

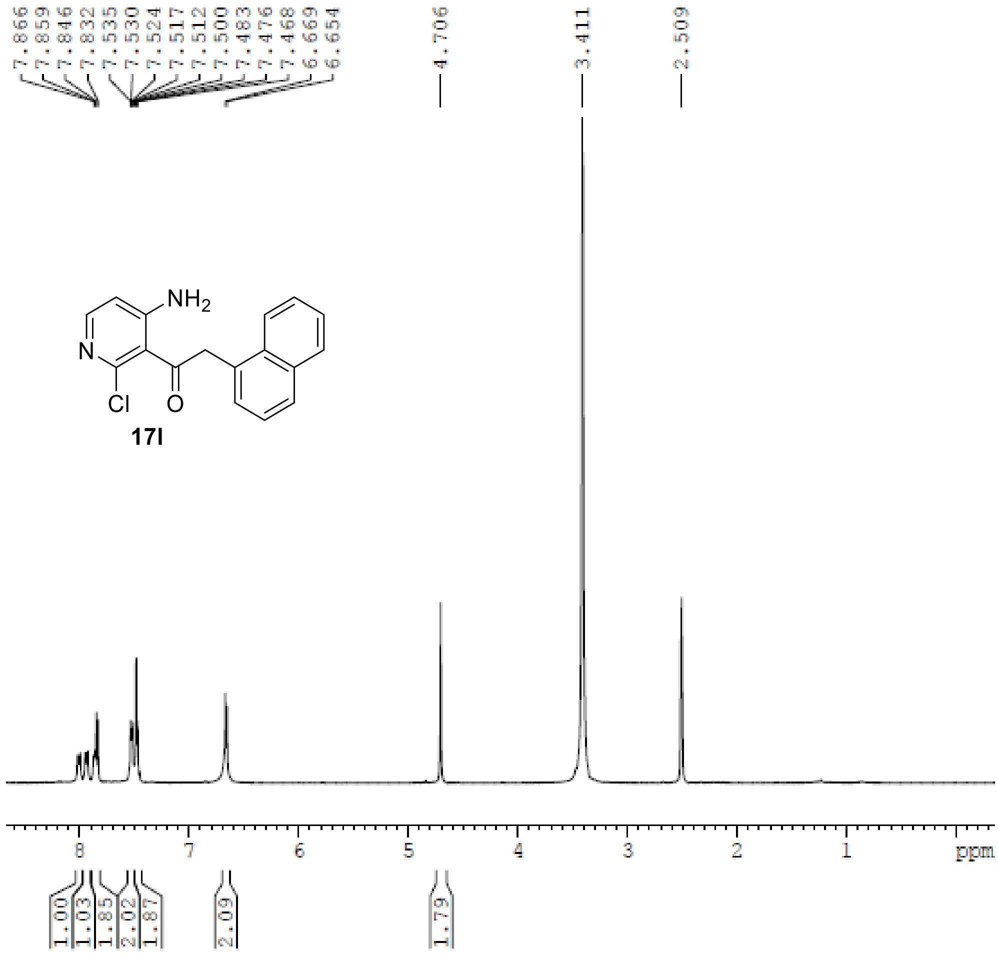

Figure S23: 400 MHz spectrum of ^{1}H-NMR of compound **17l** (DMSO-d_6)

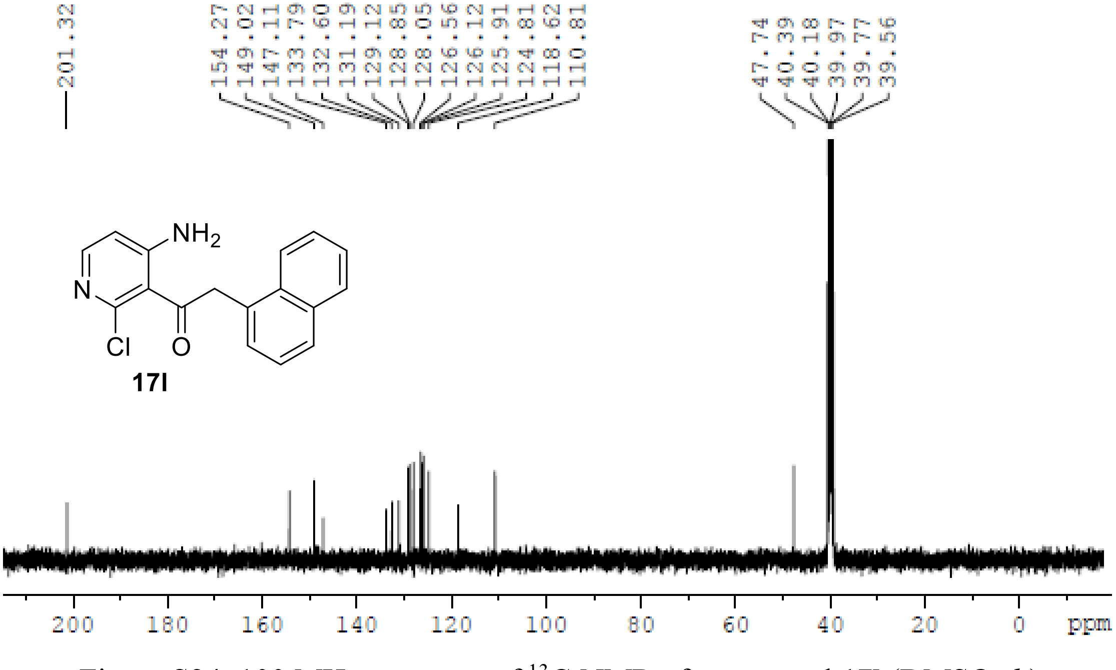

Figure S24: 100 MHz spectrum of ^{13}C-NMR of compound **17l** (DMSO-d_6)

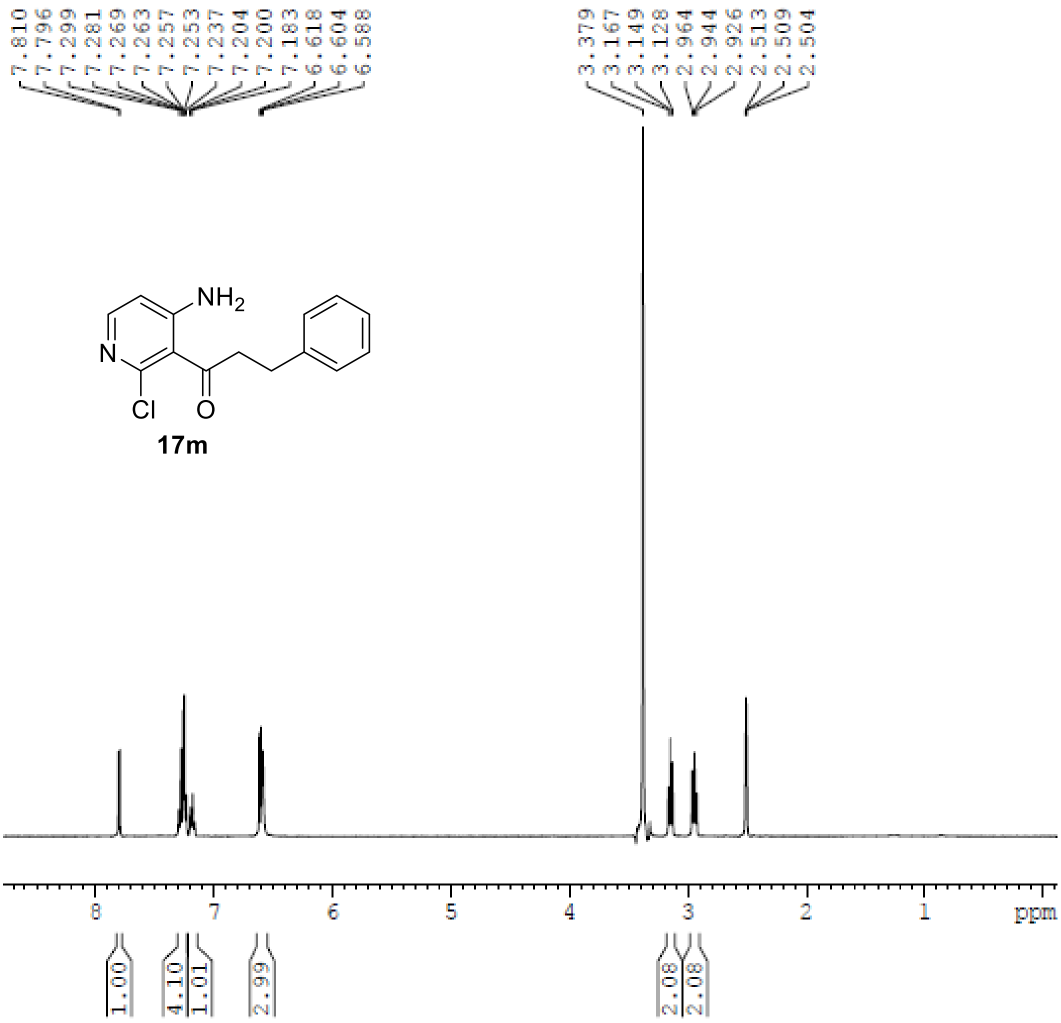

Figure S25: 400 MHz spectrum of ^{1}H-NMR of compound **17m** (DMSO-d_6)

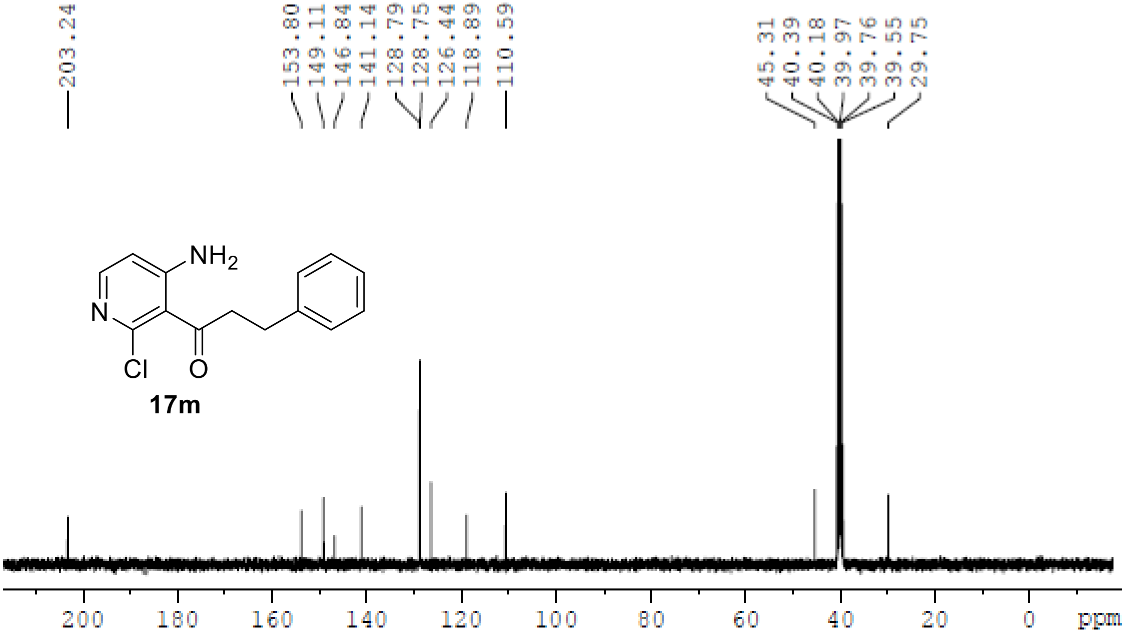

Figure S26: 100 MHz spectrum of ^{13}C-NMR of compound **17m** (DMSO-d_6)

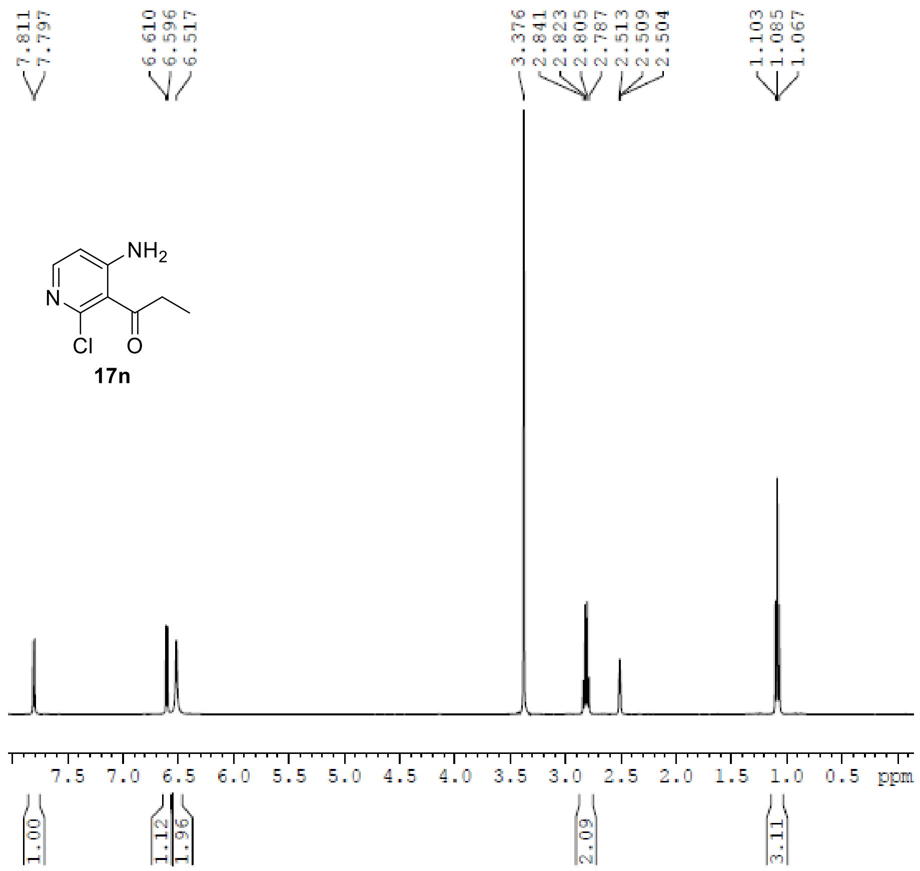

Figure S27: 400 MHz spectrum of ^{1}H-NMR of compound **17n** (DMSO-d_6)

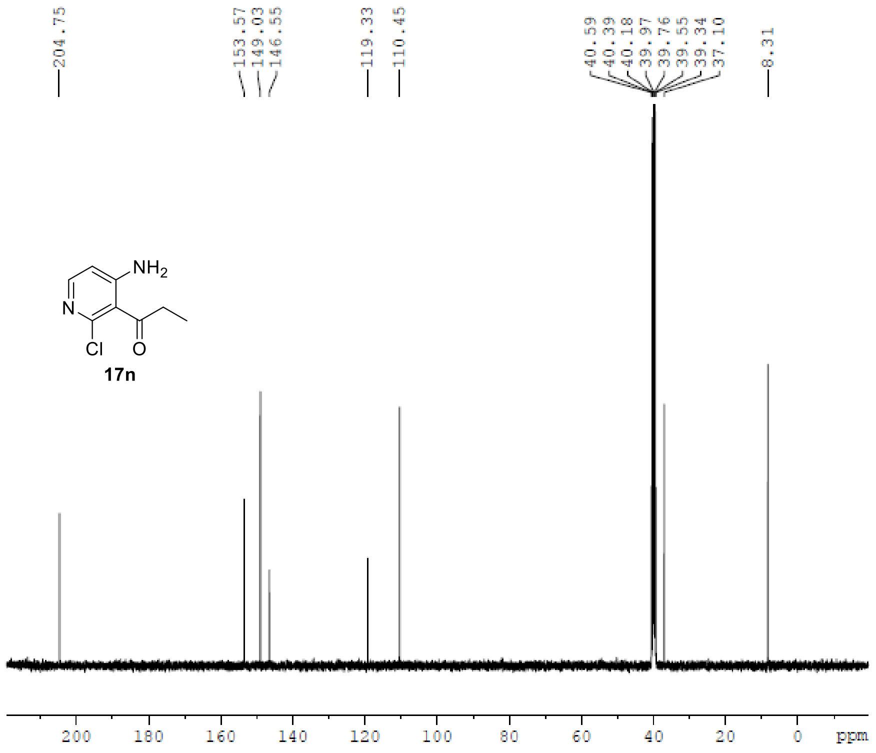

Figure S28: 100 MHz spectrum of ^{13}C-NMR of compound **17n** (DMSO-d_6)

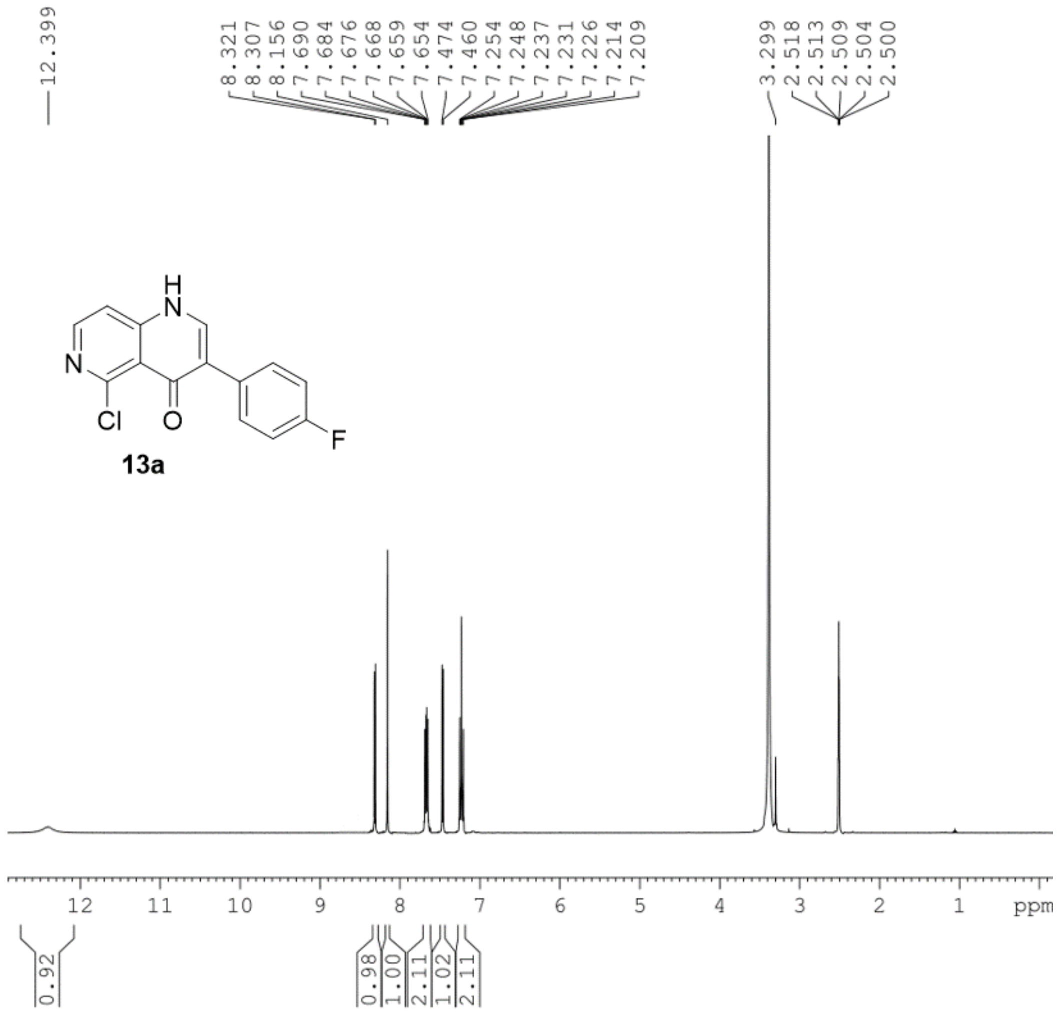

Figure S29: 400 MHz spectrum of ^{1}H-NMR of compound **13a** (DMSO-d_6)

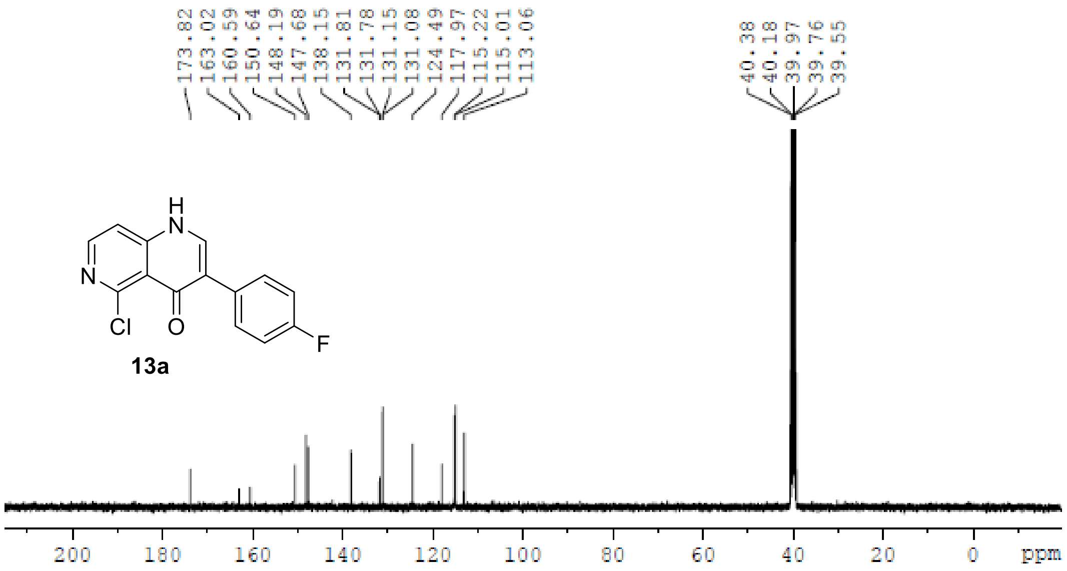

Figure S30: 100 MHz spectrum of ^{13}C-NMR of compound **13a** (DMSO-d_6)

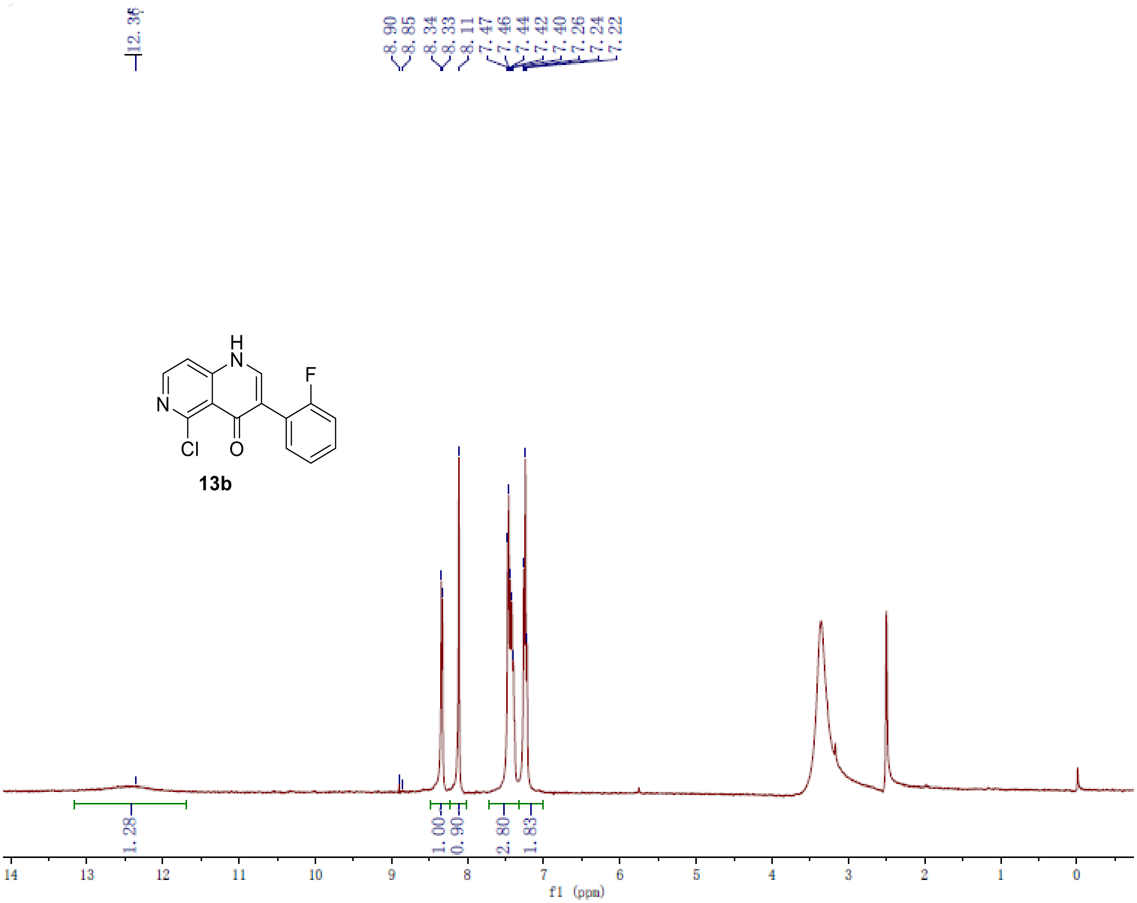

Figure S31: 400 MHz spectrum of ^{1}H-NMR of compound **13b** (DMSO-d_6)

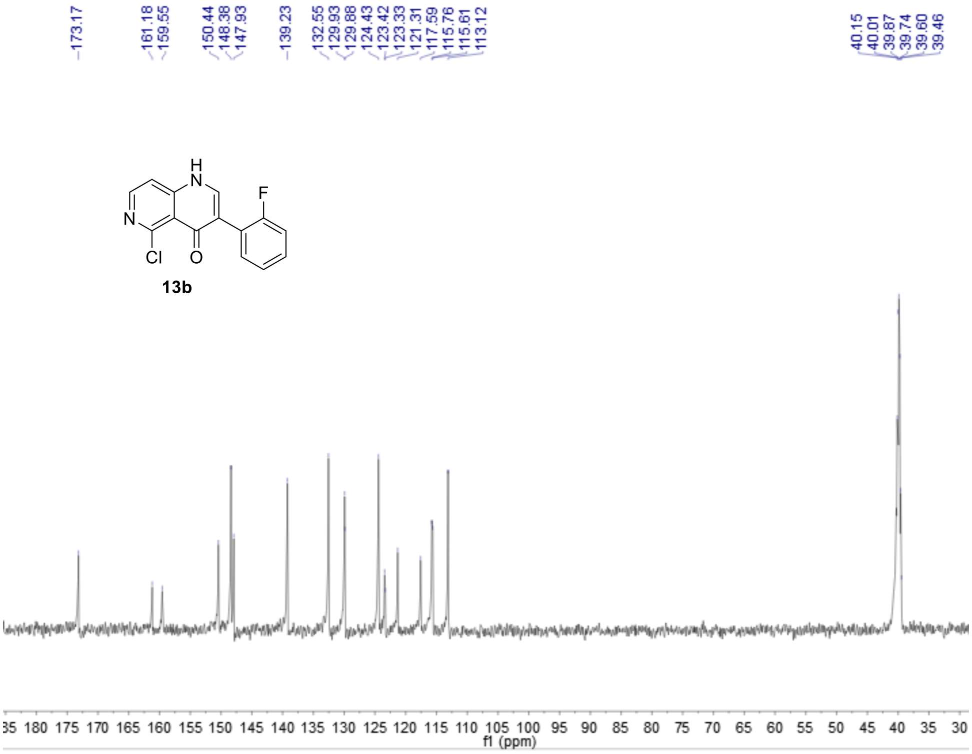

Figure S32: 150 MHz spectrum of ^{13}C-NMR of compound **13b** (DMSO-d_6)

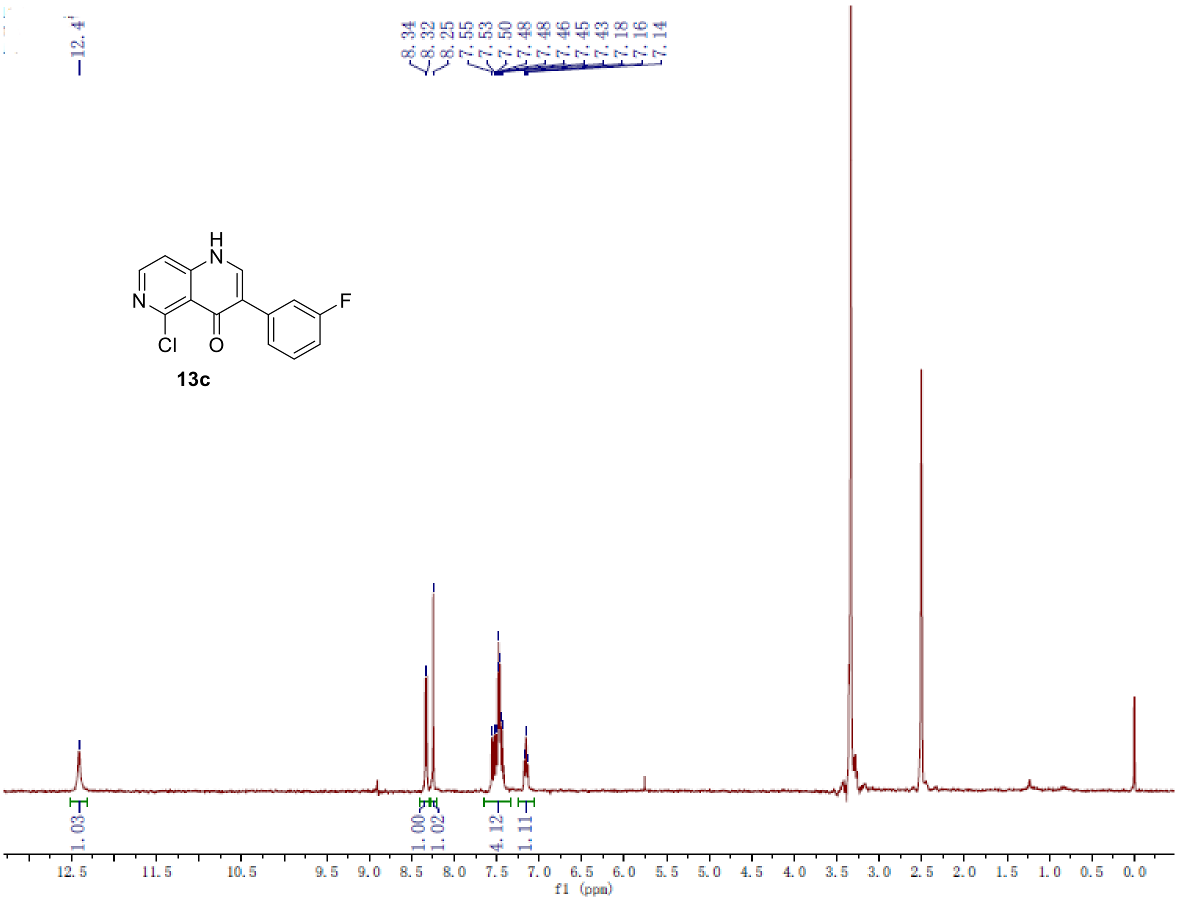

Figure S33: 400 MHz spectrum of ^{1}H-NMR of compound **13c** (DMSO-d_6)

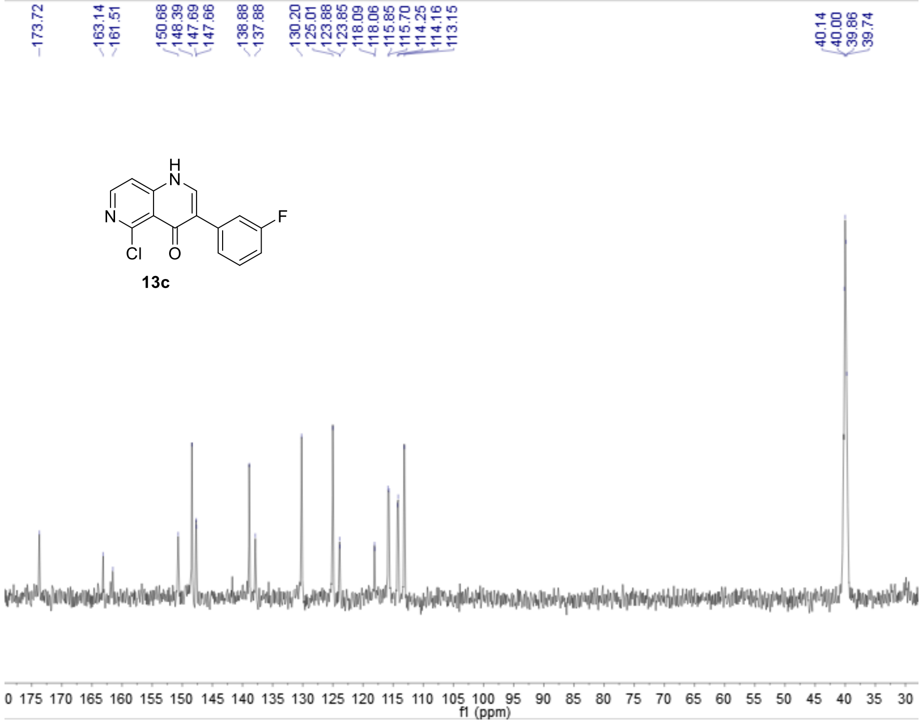

Figure S34: 150 MHz spectrum of ^{13}C-NMR of compound **13c** (DMSO-d_6)

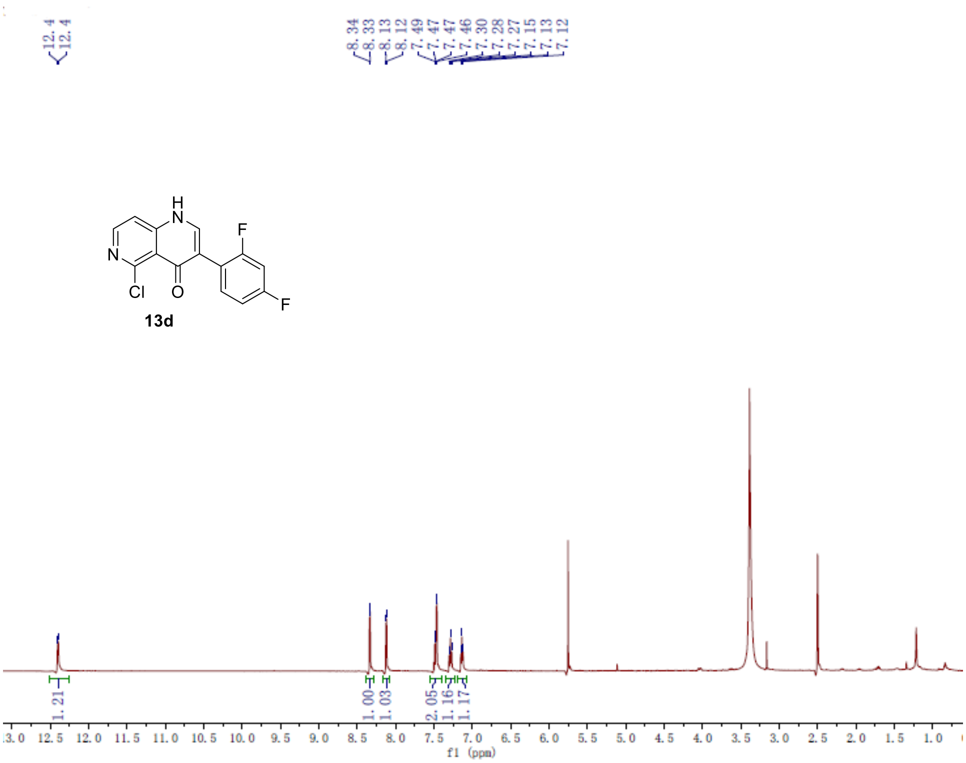

Figure S35: 400 MHz spectrum of ^{1}H-NMR of compound **13d** (DMSO-d_6)

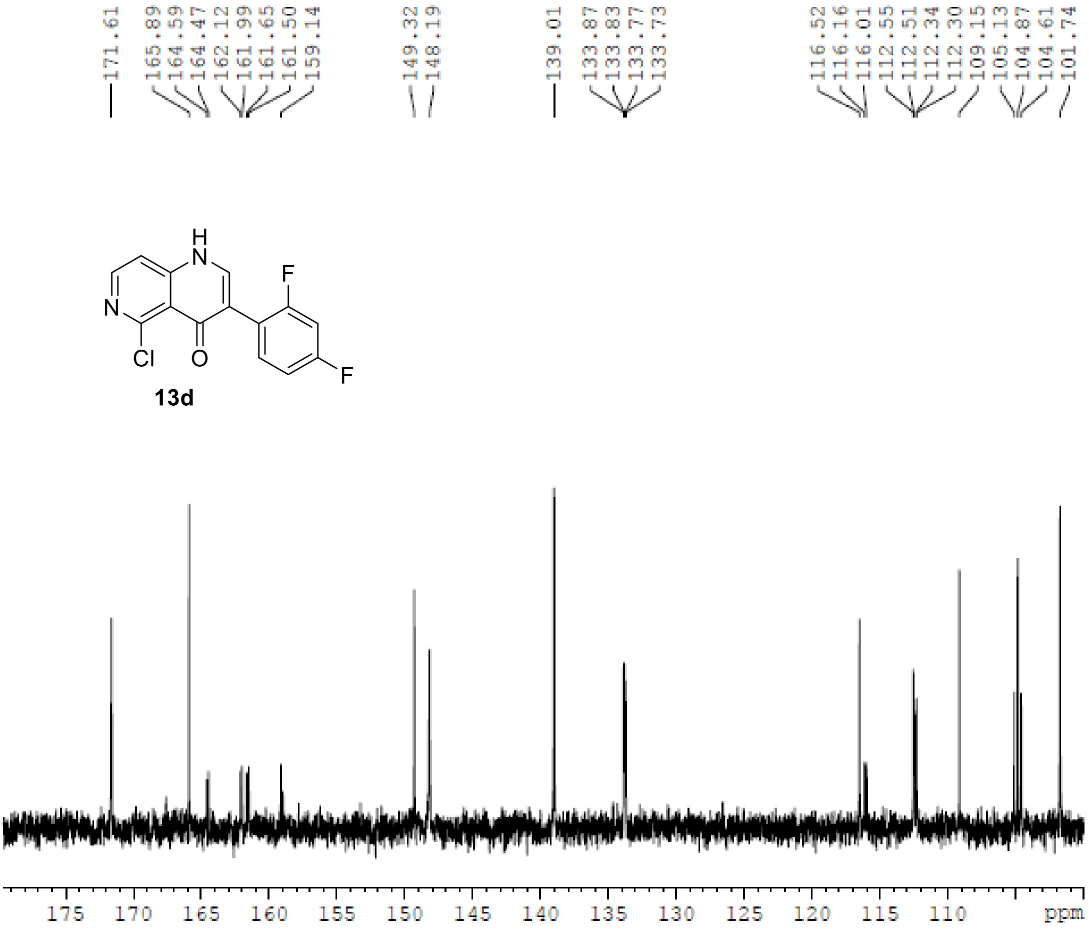

Figure S36: 100 MHz spectrum of ^{13}C-NMR of compound **13d** (DMSO-d_6)

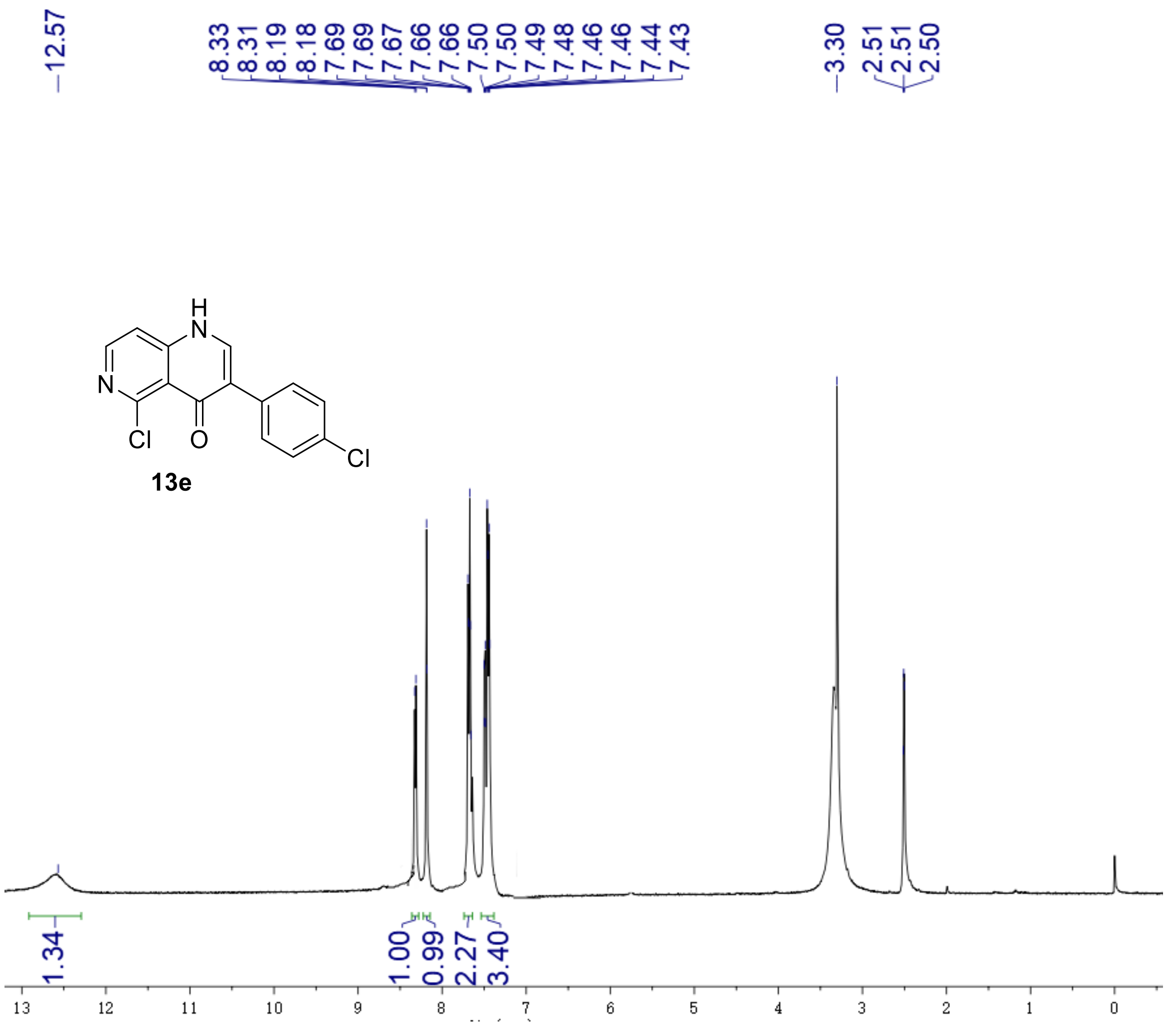

Figure S37: 400 MHz spectrum of ^{1}H-NMR of compound **13e** (DMSO-d_6)

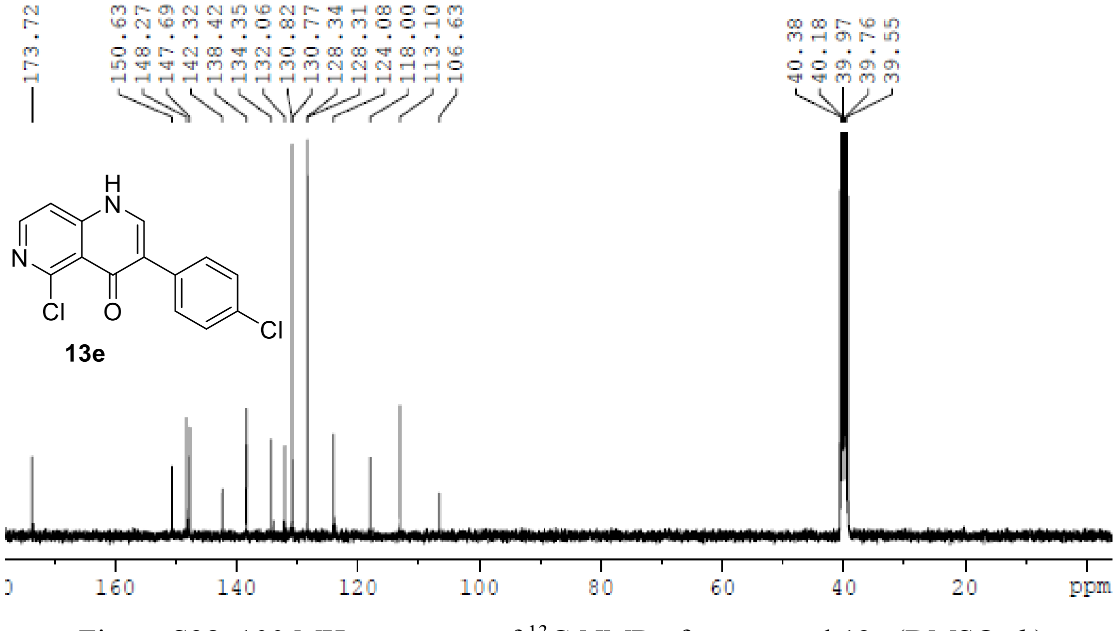

Figure S38: 100 MHz spectrum of ^{13}C-NMR of compound **13e** (DMSO-d_6)

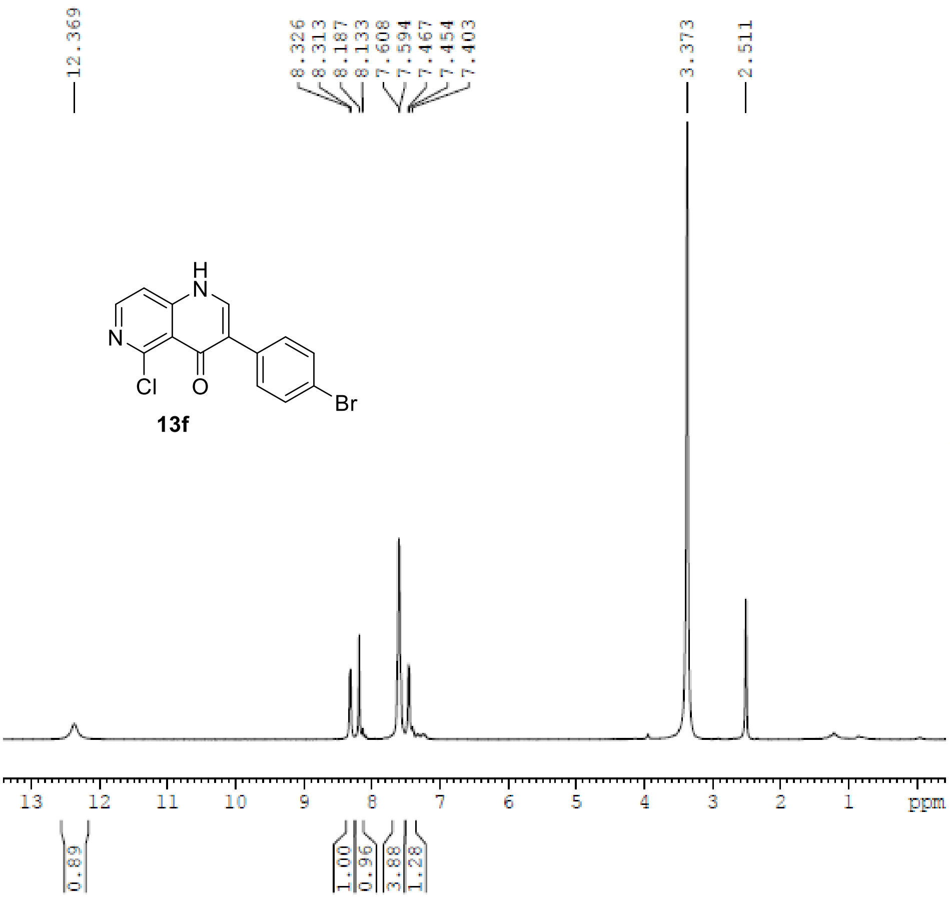

Figure S39: 400 MHz spectrum of ^{1}H-NMR of compound **13f** (DMSO-d_6)

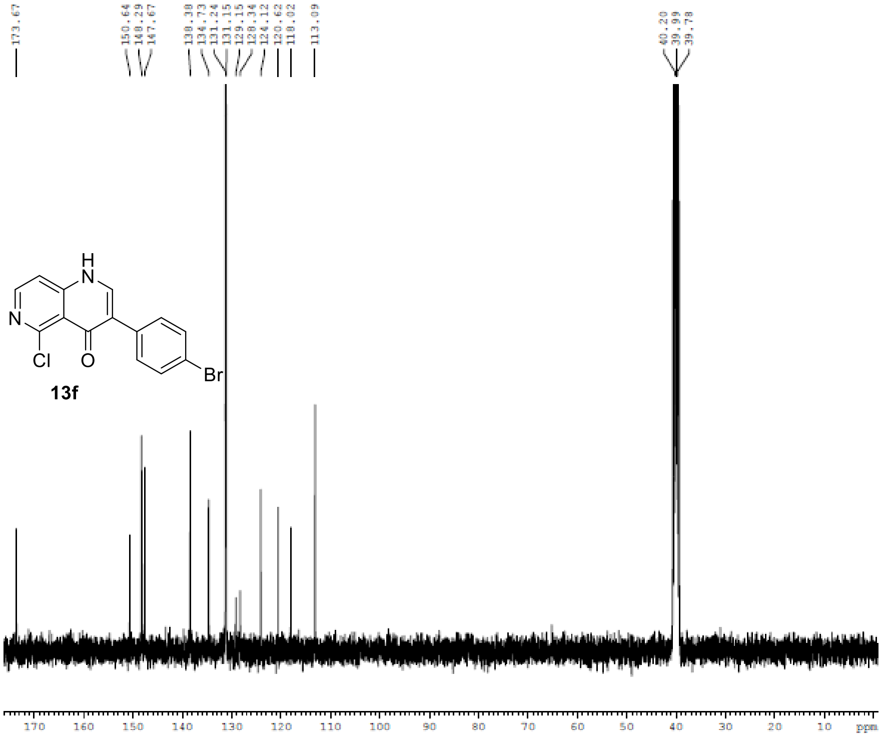

Figure S40: 100 MHz spectrum of ^{13}C-NMR of compound **13f** (DMSO-d_6)

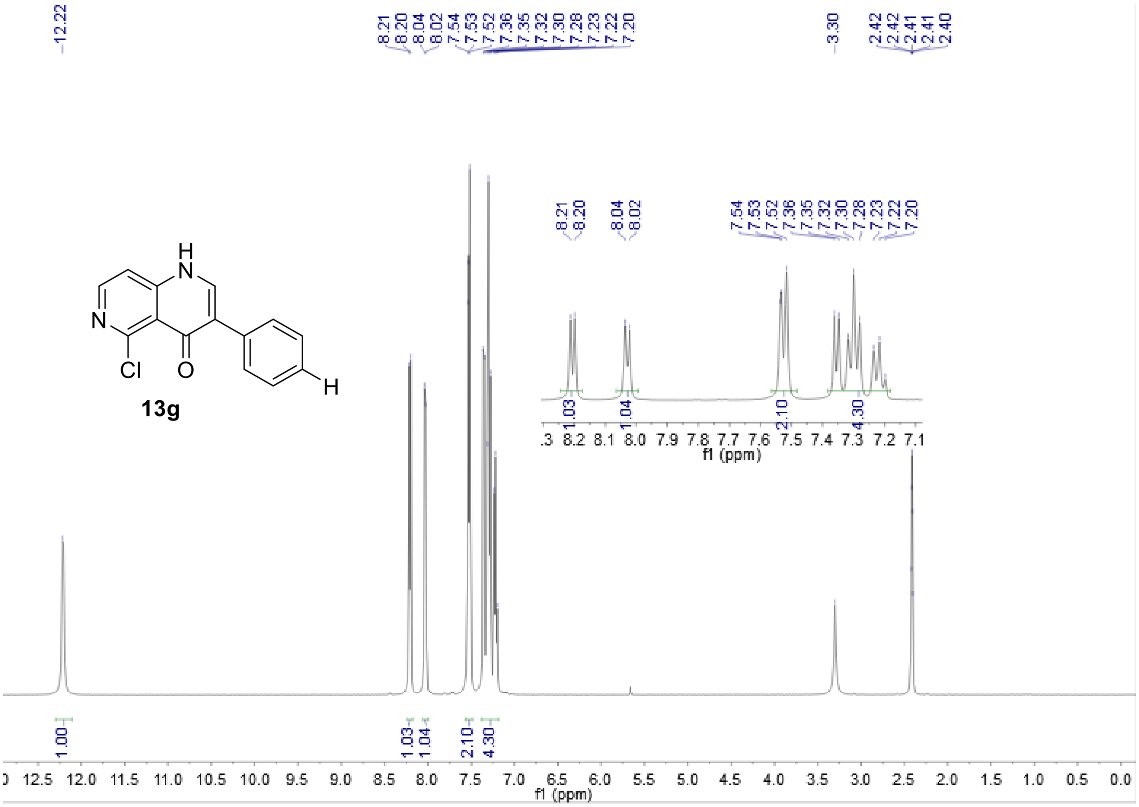

Figure S41: 400 MHz spectrum of ^{1}H-NMR of compound **13g** (DMSO-d_6)

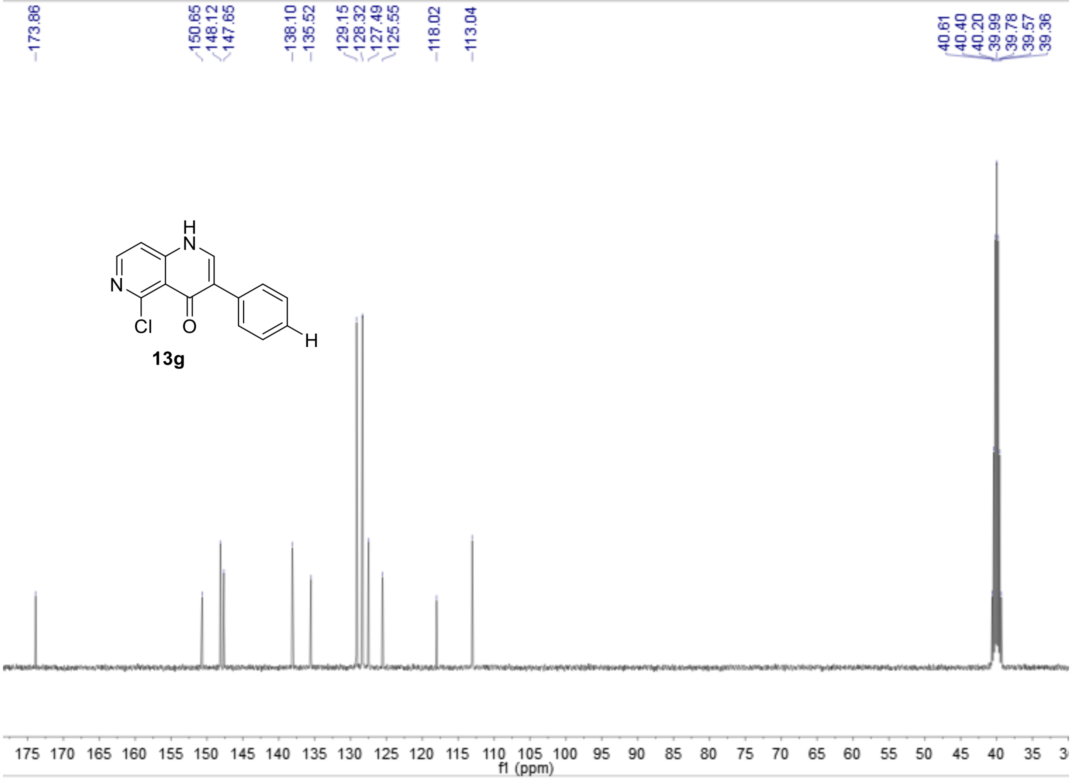

Figure S42: 100 MHz spectrum of ^{13}C-NMR of compound **13g** (DMSO-d_6)

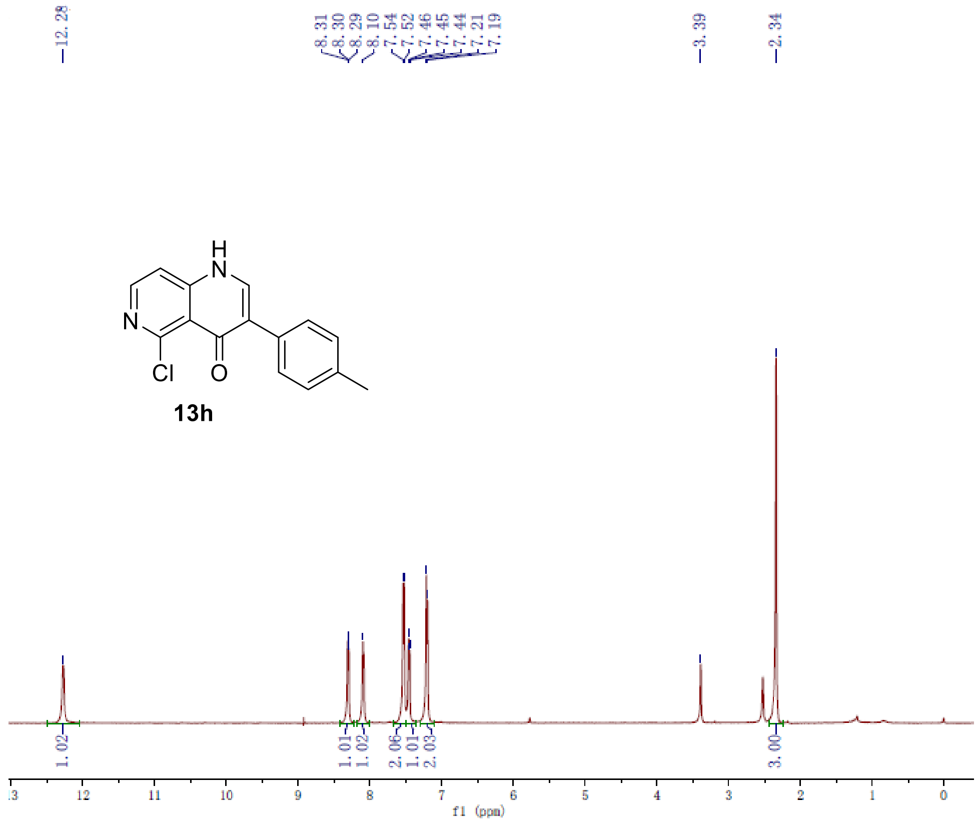

Figure S43: 400 MHz spectrum of ^{1}H-NMR of compound **13h** (DMSO-d_6)

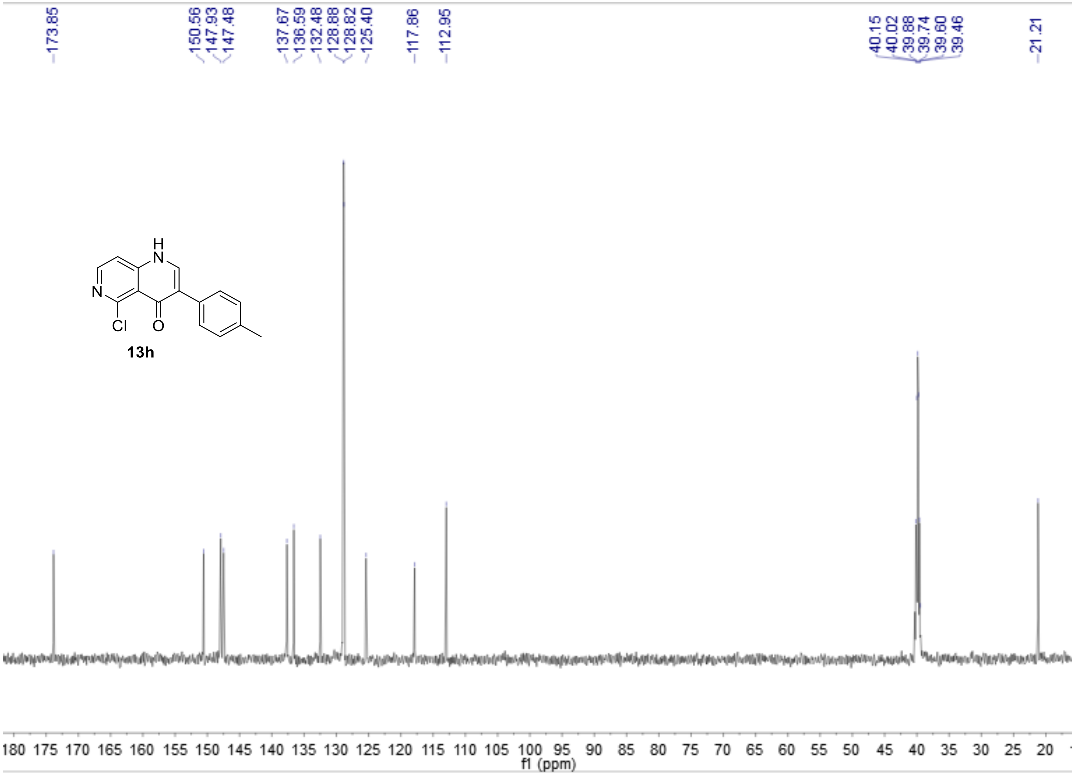

Figure S44: 150 MHz spectrum of ^{13}C-NMR of compound **13h** (DMSO-d_6)

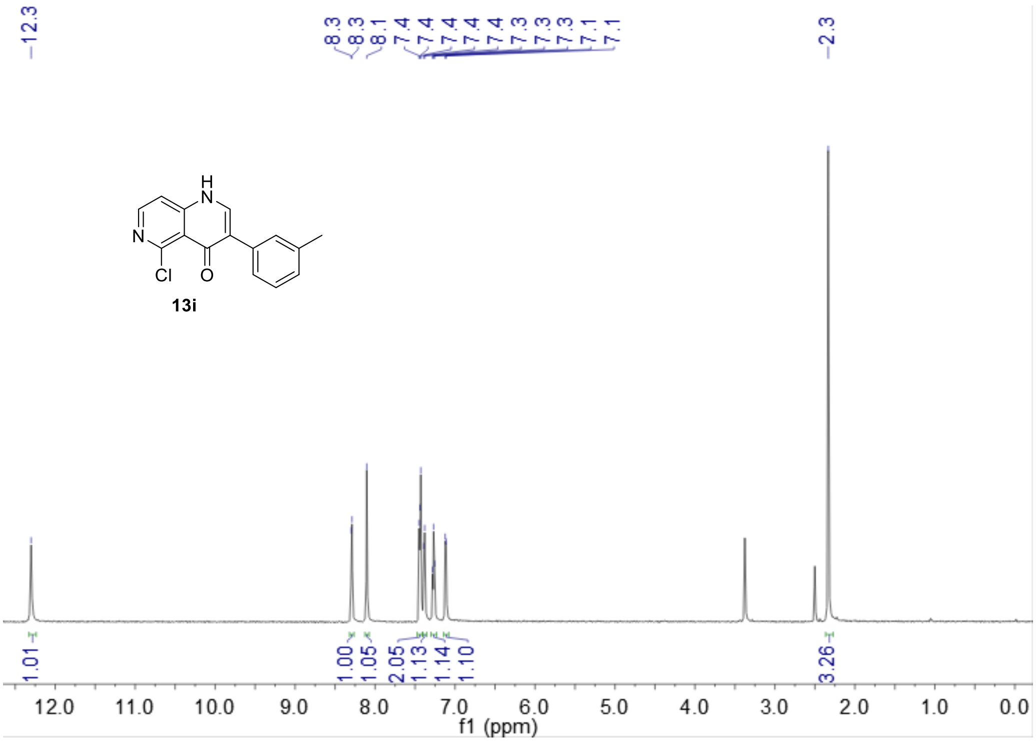

Figure S45: 600 MHz spectrum of ^{1}H-NMR of compound **13i** (DMSO-d_6)

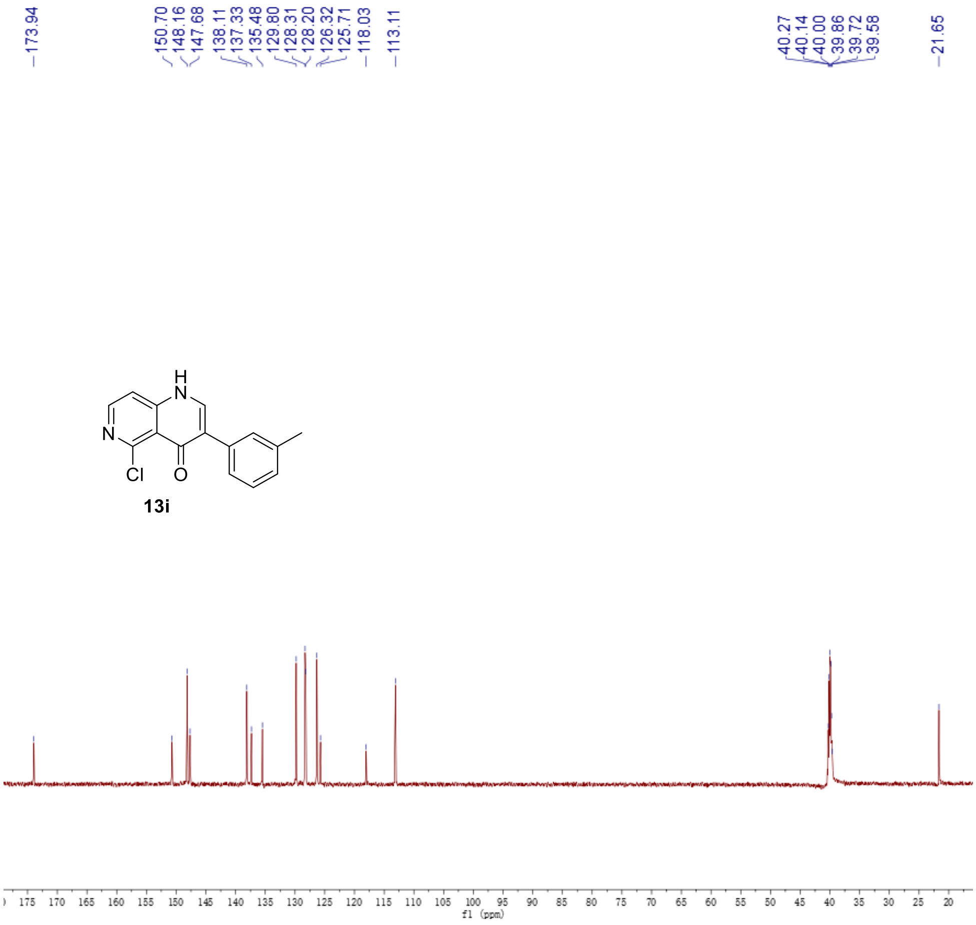

Figure S46: 150 MHz spectrum of ^{13}C-NMR of compound **13i** (DMSO-d_6)

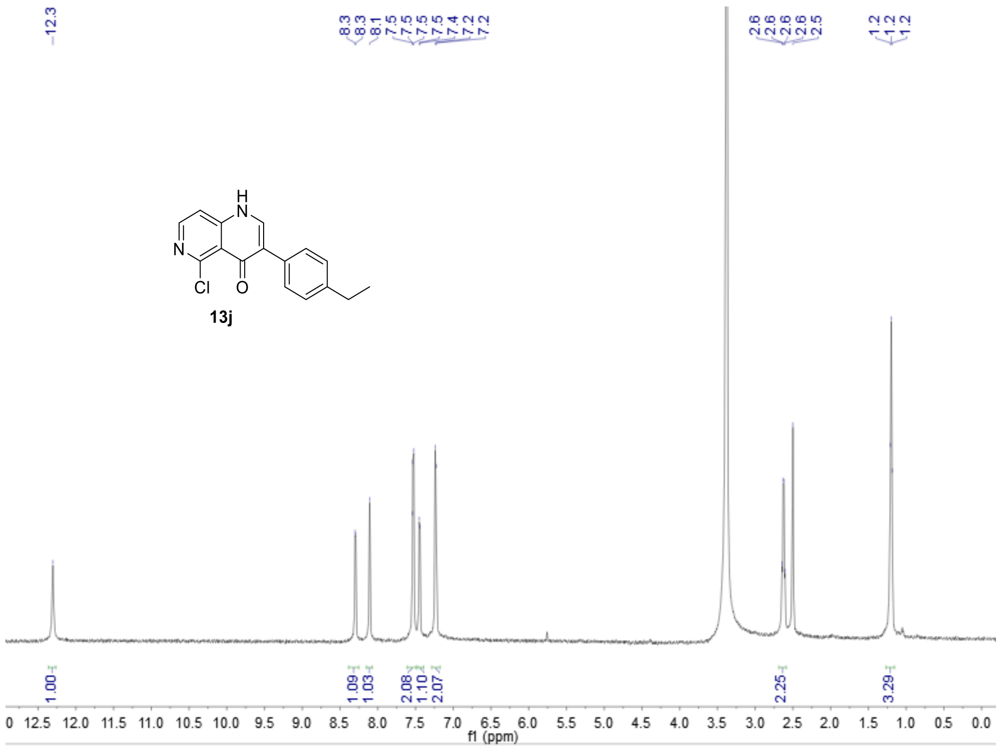

Figure S47: 600 MHz spectrum of ^{1}H-NMR of compound **13j** (DMSO-d_6)

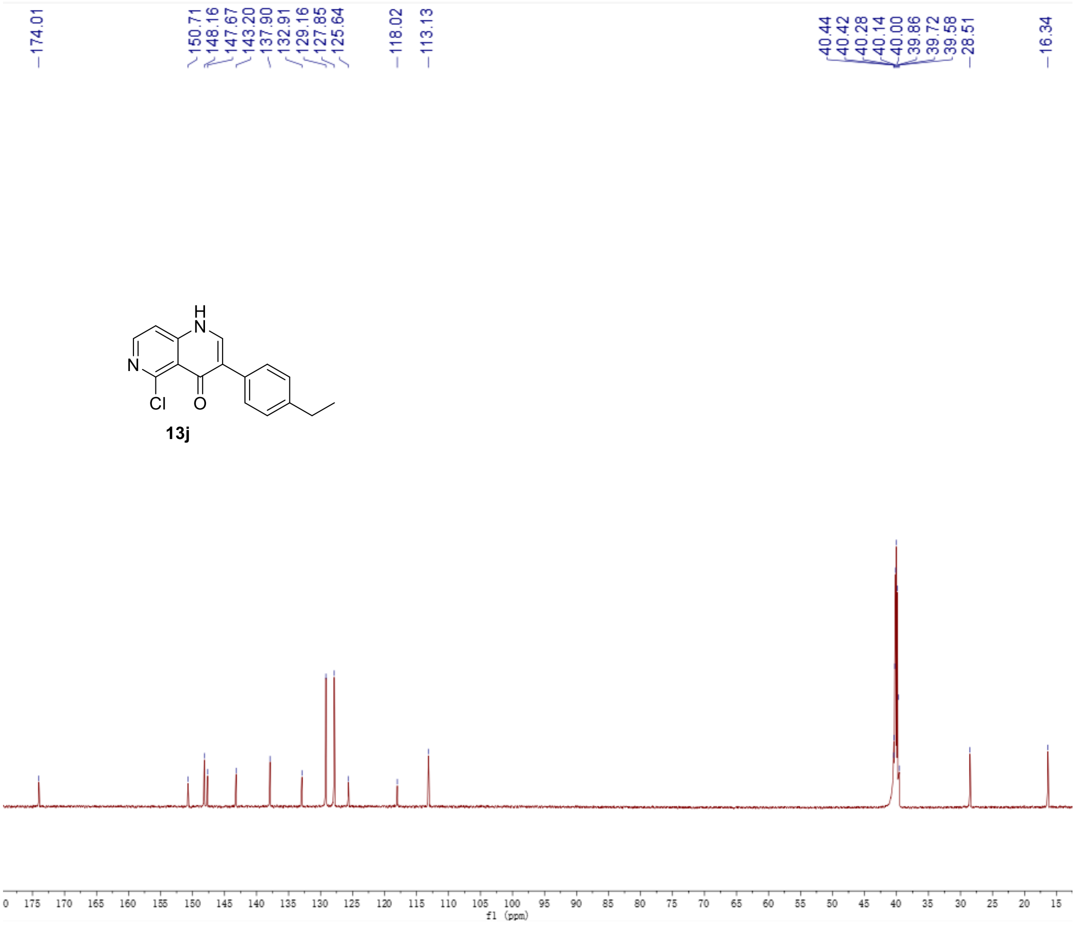

Figure S48: 150 MHz spectrum of ^{13}C-NMR of compound **13j** (DMSO-d_6)

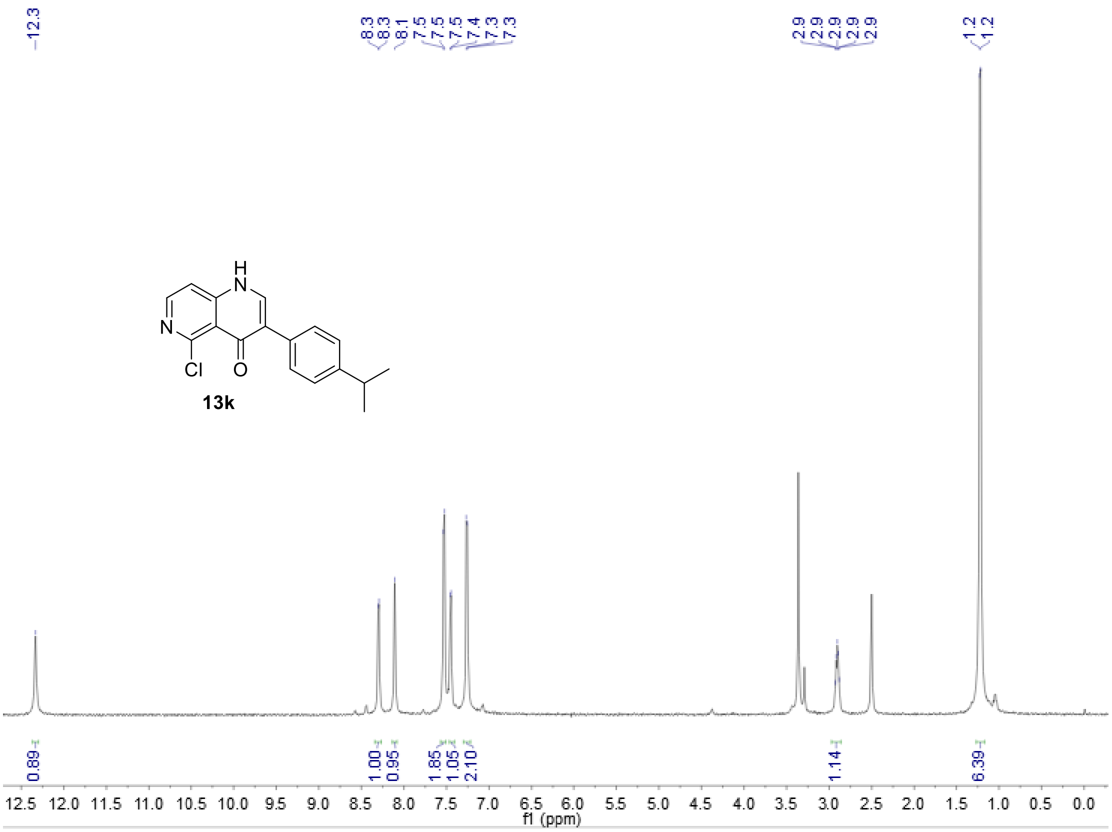

Figure S49: 600 MHz spectrum of ^{1}H-NMR of compound **13k** (DMSO-d_6)

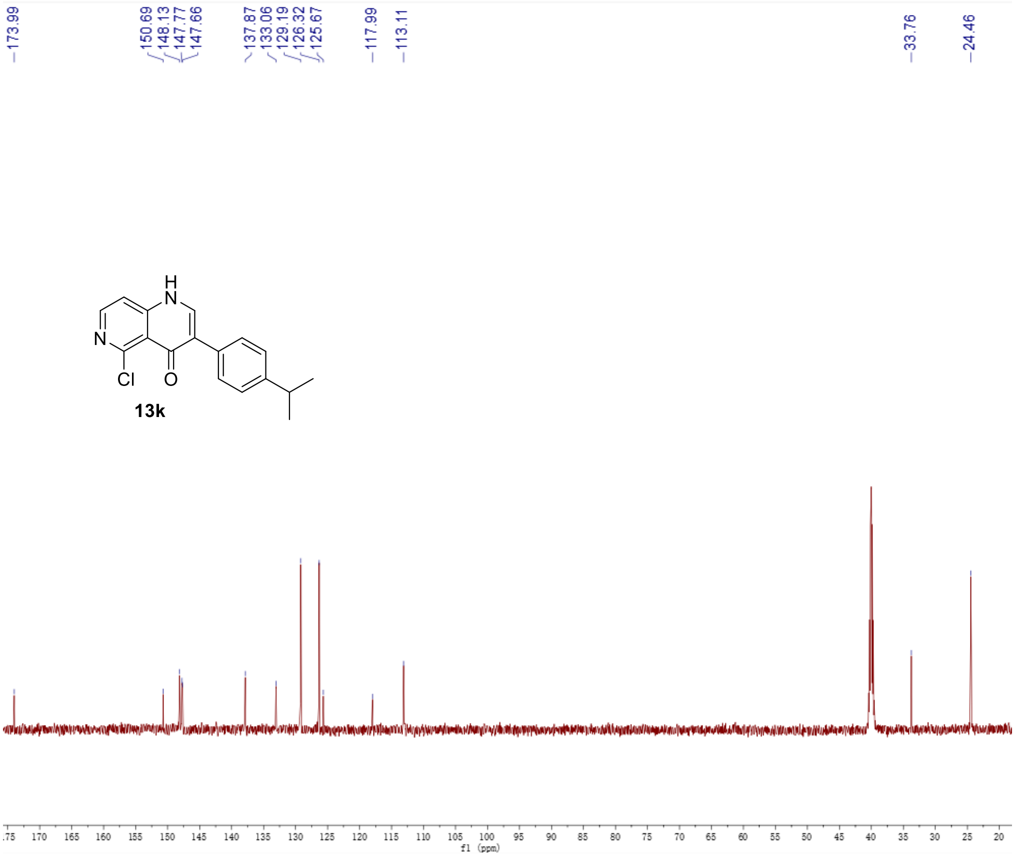

Figure S50: 150 MHz spectrum of ^{13}C-NMR of compound **13k** (DMSO-d_6)

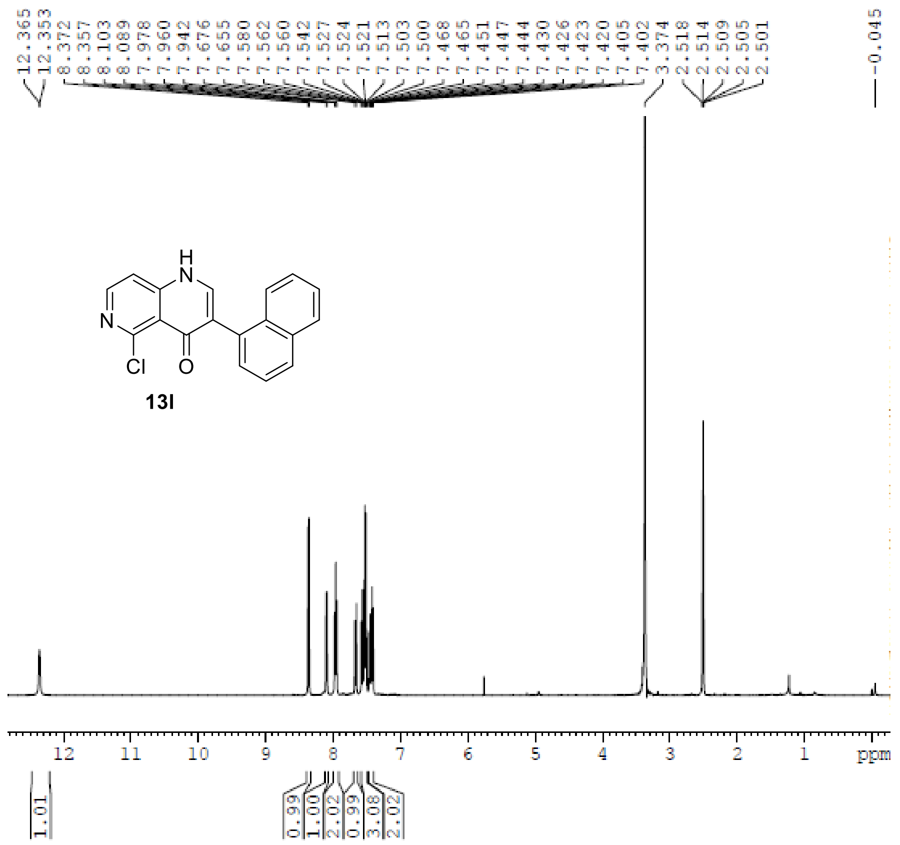

Figure S51: 400 MHz spectrum of ^{1}H-NMR of compound **13l** (DMSO-d_6)

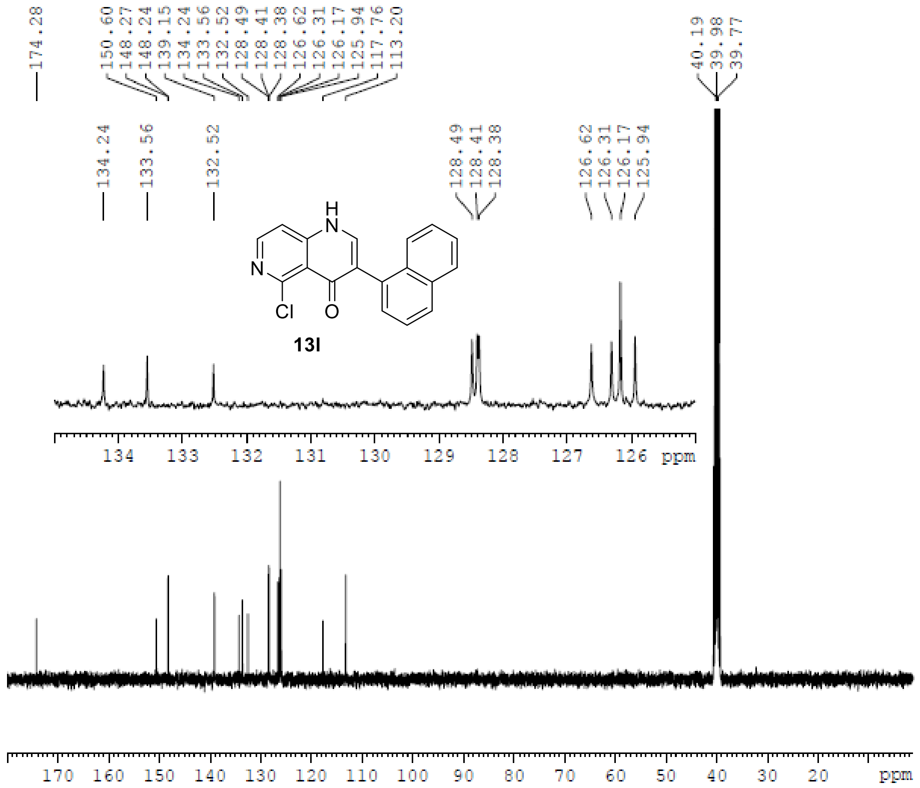

Figure S52: 100 MHz spectrum of ^{13}C-NMR of compound **13l** (DMSO-d_6)

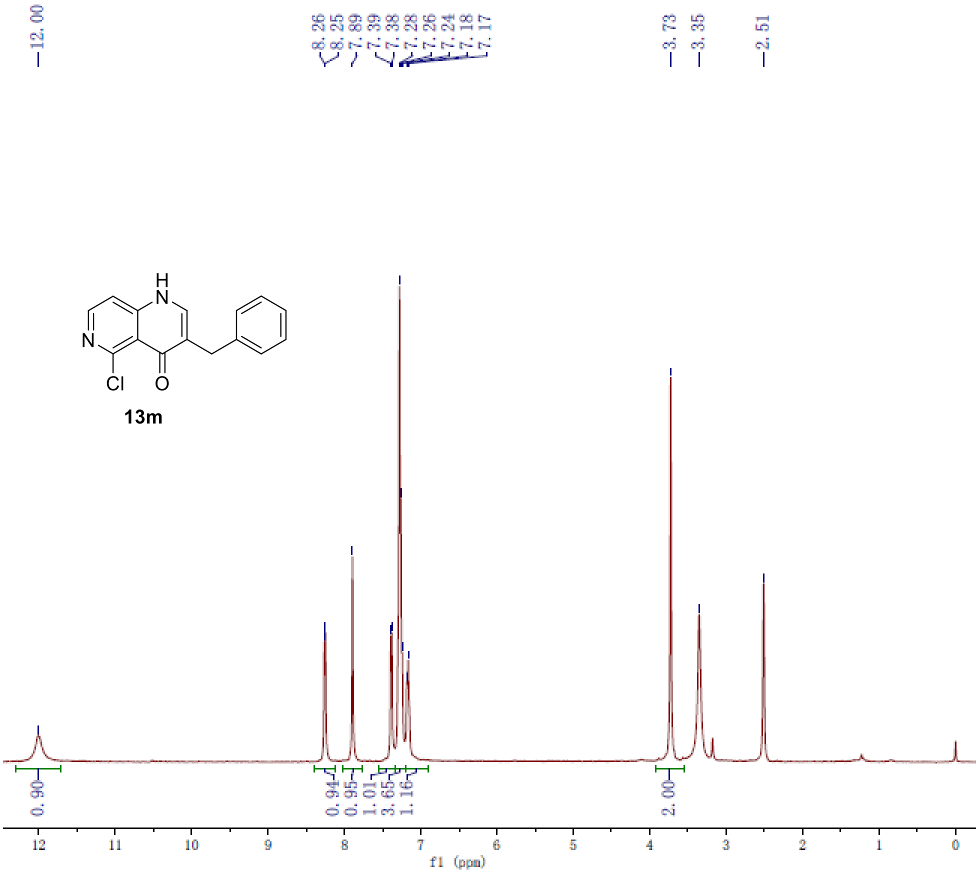

Figure S53: 400 MHz spectrum of ^{1}H-NMR of compound **13m** (DMSO-d_6)

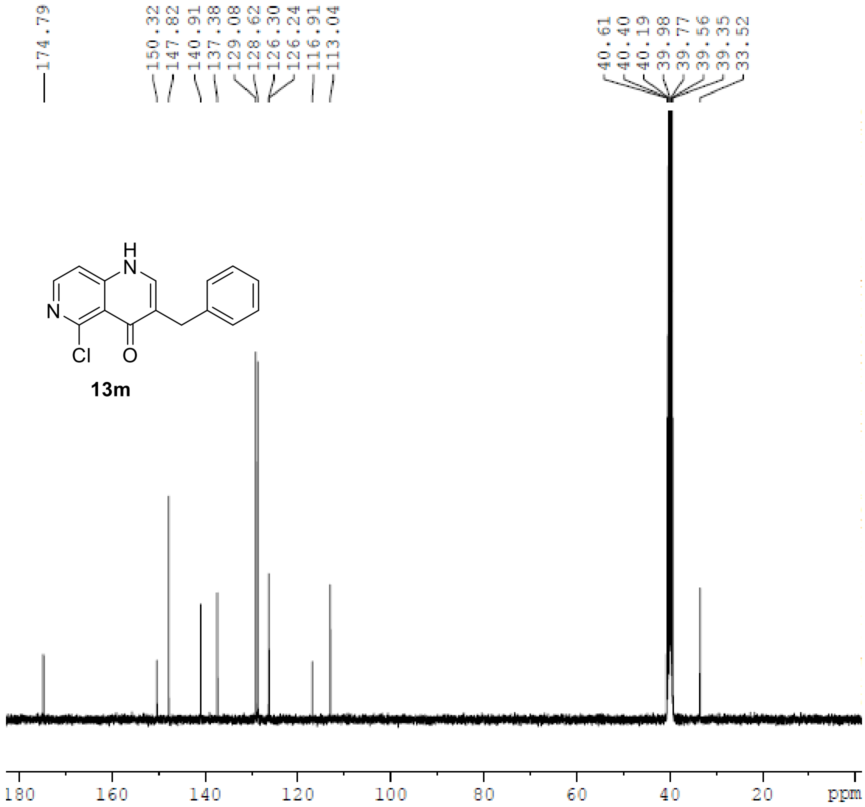

Figure S54: 100 MHz spectrum of ^{13}C-NMR of compound **13m** (DMSO-d_6)

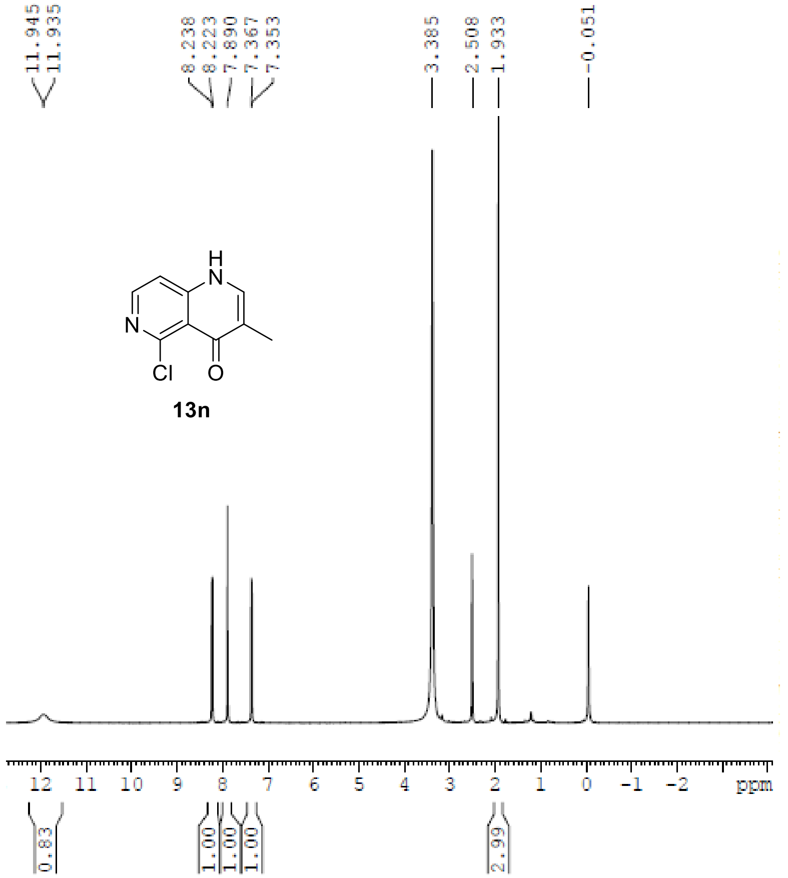

Figure S55: 400 MHz spectrum of ^{1}H-NMR of compound **13n** (DMSO-d_6)

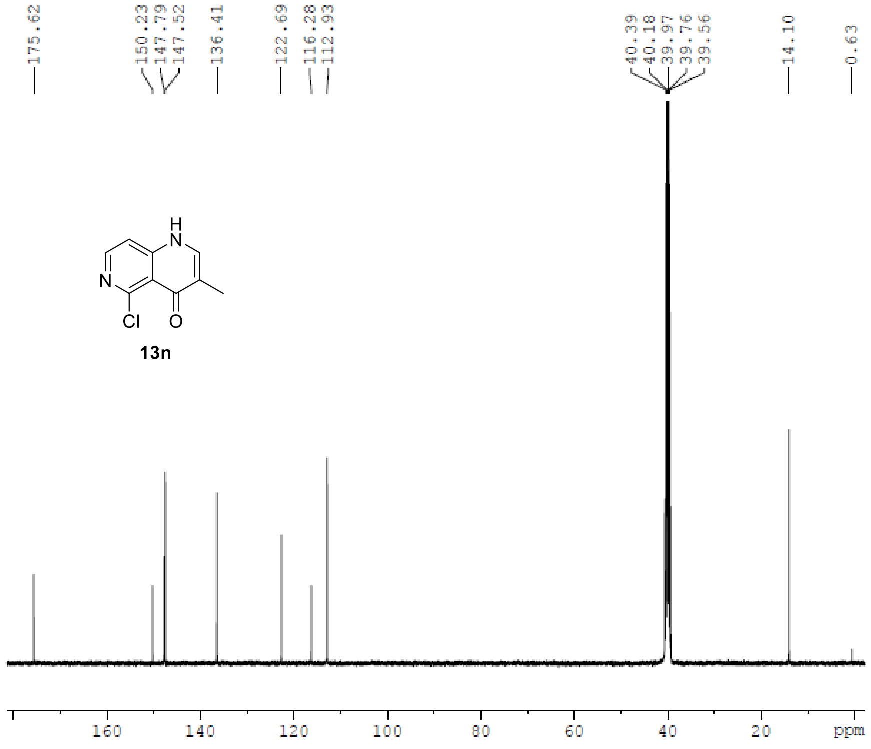

Figure S56: 100 MHz spectrum of ^{13}C-NMR of compound **13n** (DMSO-d_6)

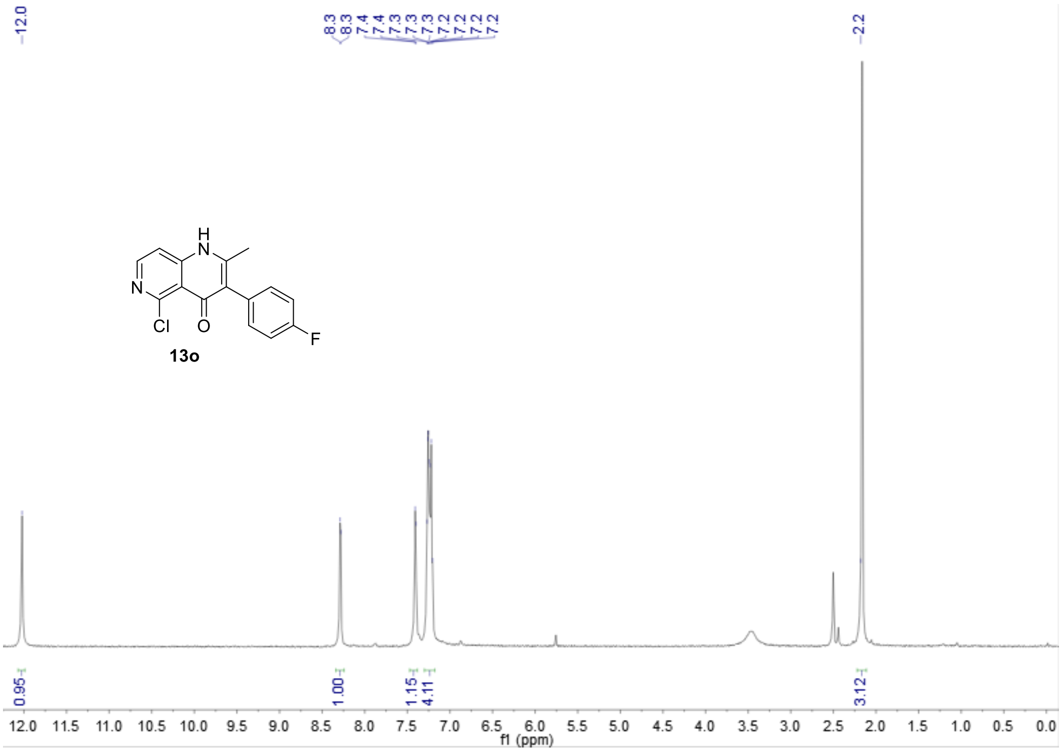

Figure S57: 600 MHz spectrum of ^{1}H-NMR of compound **13o** (DMSO-d_6)

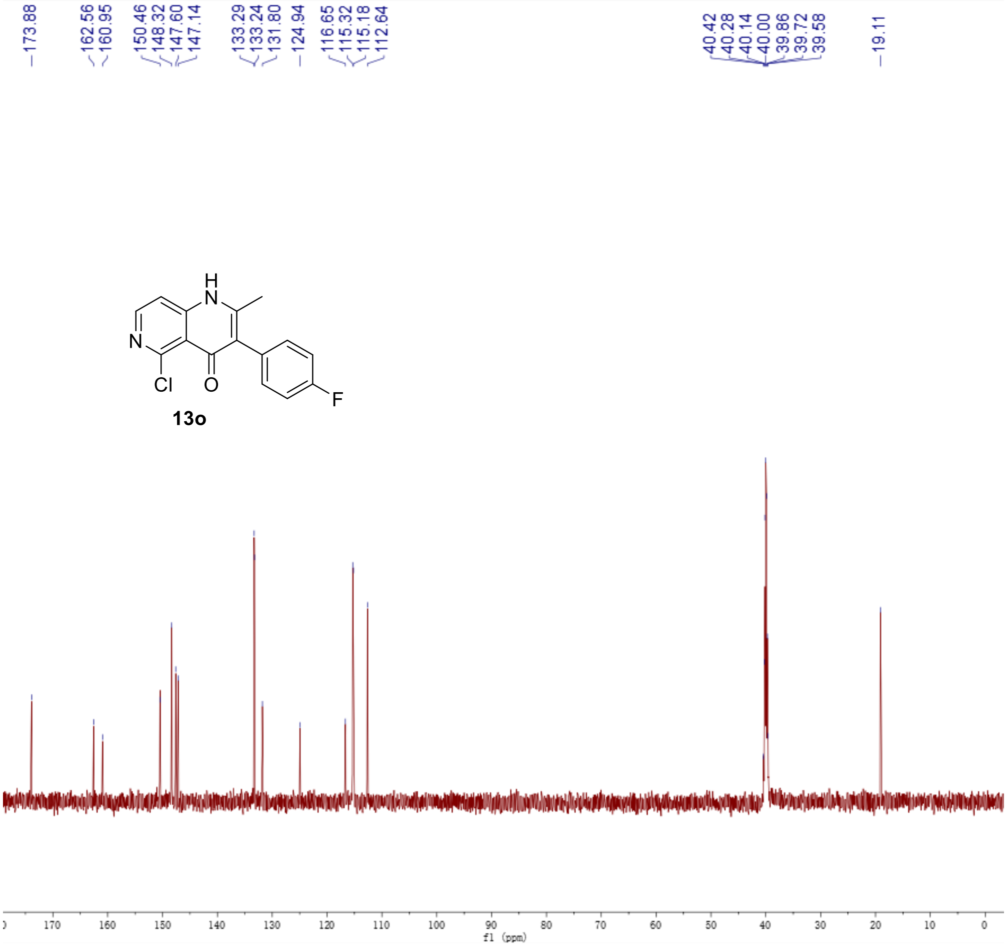

Figure S58: 150 MHz spectrum of ^{13}C-NMR of compound **13o** (DMSO-d_6)
